# Binodal, wireless epidermal electronic systems with in-sensor analytics for neonatal intensive care

**DOI:** 10.1126/science.aau0780

**Published:** 2019-03-01

**Authors:** Ha Uk Chung, Bong Hoon Kim, Jong Yoon Lee, Jungyup Lee, Zhaoqian Xie, ErinM. Ibler, KunHyuck Lee, Anthony Banks, JiYoon Jeong, Jongwon Kim, Christopher Ogle, Dominic Grande, Yongjoon Yu, Hokyung Jang, Pourya Assem, Dennis Ryu, JeanWon Kwak, Myeong Nam koong, Jun Bin Park, Yechan Lee, Do Hoon Kim, Arin Ryu, Jaeseok Jeong, Kevin You, Bowen Ji, Zhuangjian Liu, Qingze Huo, Xue Feng, Yujun Deng, Yeshou Xu, Kyung-In Jang, Jeonghyun Kim, Yihui Zhang, Roozbeh Ghaffari, Casey M. Rand, Molly Schau, Aaron Hamvas, Debra E. Weese-Mayer, Yonggang Huang, SeungMin Lee, ChiHwan Lee, Naresh R. Shanbhag, Amy S. Paller, Shuai Xu, John A. Rogers

**Affiliations:** 1 Simpson Querrey Institute, Northwestern University, Chicago, IL 60611, USA; 2 Department of Electrical Engineering and Computer Science, Northwestern University, Evanston, IL 60208, USA; 3 Department of Materials Science and Engineering, Northwestern University, Evanston, IL 60208, USA; 4 Frederick Seitz Materials Research Laboratory, University of Illinois at Urbana-Champaign, Urbana, IL 61801, USA; 5 Center for Bio-integrated Electronics, Northwestern University, Evanston, IL 60208, USA; 6 Department of Electrical and Computer Engineering, University of Illinois at Urbana-Champaign, Urbana, IL 61801, USA; 7 Department of Civil and Environmental Engineering, Northwestern University, Evanston, IL 60208, USA; 8 Department of Mechanical Engineering, Northwestern University, Evanston, IL 60208, USA; 9 Department of Dermatology, Feinberg School of Medicine, Northwestern University, Chicago, IL 60611, USA; 10 Center for Autonomic Medicine, Department of Pediatrics, Ann & Robert H. Lurie Children’s Hospital of Chicago, Chicago, IL 60611, USA; 11 Loomis Laboratory of Physics, University of Illinois at Urbana-Champaign, Urbana, IL 61801, USA; 12 Department of Mechanical Engineering, Kyung Hee University, Yongin 17104, Republic of Korea; 13 Department of Biomedical Engineering, Northwestern University, Evanston, IL 60208, USA; 14 Department of Micro/Nano Electronics, Shanghai Jiao Tong University, Shanghai 200240, China; 15 Institute of High Performance Computing, A*Star, 138632 Singapore; 16 Applied Mechanics Laboratory, Department of Engineering Mechanics, Center for Mechanics and Materials, Center for Flexible Electronics Technology, Tsinghua University, Beijing 100084, China; 17 State Key Laboratory of Mechanical System and Vibration, Shanghai Jiao Tong University, Shanghai 200240, China; 18 Key Laboratory of C&PC Structures of the Ministry of Education, Southeast University, Nanjing 2100096, China; 19 Department of Robotics Engineering, Daegu Gyeongbuk Institute of Science and Technology (DGIST), Daegu 42988, Republic of Korea; 20 Department of Electronics Convergence Engineering, Kwangwoon University, Seoul 01897, Republic of Korea; 21 Stanley Manne Children’s Research Institute, Ann & Robert H. Lurie Children’s Hospital of Chicago, Chicago, IL 60611, USA; 22 Division of Neonatology, Department of Pediatrics, Ann & Robert H. Lurie Children’s Hospital of Chicago, Chicago, IL 60611, USA; 23 Department of Pediatrics, Ann & Robert H. Lurie Children’s Hospital of Chicago, Chicago, IL 60611, USA; 24 Department of Energy Electronics Convergence, Kookmin University, Seoul 02707, Republic of Korea; 25 Weldon School of Biomedical Engineering, School of Mechanical Engineering, Center for Implantable Devices, and Birck Nanotechnology Center, Purdue University, West Lafayette, IN 47907, USA; 26 Department of Chemistry, Northwestern University, Evanston, IL 60208, USA; 27 Department of Neurological Surgery, Feinberg School of Medicine, Northwestern University, Chicago, IL 60611, USA

## Abstract

Existing vital signmonitoring systems in the neonatal intensive care unit (NICU) requiremultiple wires connected to rigid sensors with strongly adherent interfaces to the skin.We introduce a pair of ultrathin, soft, skin-like electronic devices whose coordinated, wireless operation reproduces the functionality of these traditional technologies but bypasses their intrinsic limitations.The enabling advances in engineering science include designs that support wireless, battery-free operation; real-time, in-sensor data analytics; time-synchronized, continuous data streaming; soft mechanics and gentle adhesive interfaces to the skin; and compatibility with visual inspection and with medical imaging techniques used in the NICU. Preliminary studies on neonates admitted to operating NICUs demonstrate performance comparable to the most advanced clinical-standard monitoring systems.

**INTRODUCTION:**

In neonatal intensive care units (NICUs), continuous monitoring of vital signs is essential, particularly in cases of severe prematurity. Currentmonitoring platforms requiremultiple hard-wired, rigid interfaces to a neonate’s fragile, underdeveloped skin and, in some cases, invasive lines inserted into their delicate arteries. These platforms and theirwired interfaces pose risks for iatrogenic skin injury, create physical barriers for skin-to-skin parental/neonate bonding, and frustrate even basic clinical tasks. Technologies that bypass these limitations and provide additional, advanced physiological monitoring capabilities would directly address an unmet clinical need for a highly vulnerable population.

**RATIONALE:**

It is now possible to fabricate wireless, battery-free vital signs monitoring systems based on ultrathin, “skin-like” measurement modules. These devices can gently and noninvasively interface onto the skin of neonateswith gestational ages down to the edge of viability. Four essential advances in engineering science serve as the foundations for this technology: (i) schemes for wireless power transfer, low-noise sensing, and high-speed data communications via a single radio-frequency linkwith negligible absorption in biological tissues; (ii) efficient algorithms for real-time data analytics, signal processing, and dynamic baseline modulation implemented on the sensor platforms themselves; (iii) strategies for time-synchronized streaming of wireless data from two separate devices; and (iv) designs that enable visual inspection of the skin interface while also allowing magnetic resonance imaging and x-ray imaging of the neonate. The resulting systems can be much smaller in size, lighter in weight, and less traumatic to the skin than any existing alternative.

**RESULTS:**

We report the realization of this class ofNICU monitoring technology, embodied as a pair of devices that, when used in a timesynchronized fashion, can reconstruct full vital signs information with clinical-grade precision. One device mounts on the chest to capture electrocardiograms (ECGs); the other rests on the base of the foot to simultaneously record photoplethysmograms (PPGs). This binodal system captures and continuously transmits ECG, PPG, and (fromeach device) skin temperature data, yieldingmeasurements of heart rate, heart rate variability, respiration rate, blood oxygenation, and pulse arrival time as a surrogate of systolic blood pressure. Successful tests on neonates with gestational ages ranging from 28 weeks to full term demonstrate the full range of functions in two level III NICUs.

The thin, lightweight, low-modulus characteristics of these wireless devices allow for interfaces to the skinmediated by forces that are nearly an order ofmagnitude smaller than those associated with adhesives used for conventional hardware in the NICU. This reduction greatly lowers the potential for iatrogenic injuries.

**CONCLUSION:**

The advances outlined here serve as the basis for a skin-like technology that not only reproduces capabilities currently provided by invasive, wired systems as the standard of care, but also offers multipoint sensing of temperature and continuous tracking of blood pressure, all with substantially safer device-skin interfaces and compatibility with medical imaging. By eliminating wired connections, these platforms also facilitate therapeutic skin-to-skin contact between neonates and parents, which is known to stabilize vital signs, reduce morbidity, and promote parental bonding. Beyond use in advanced hospital settings, these systems also offer costeffective capabilities with potential relevance to global health.

**Figure:**
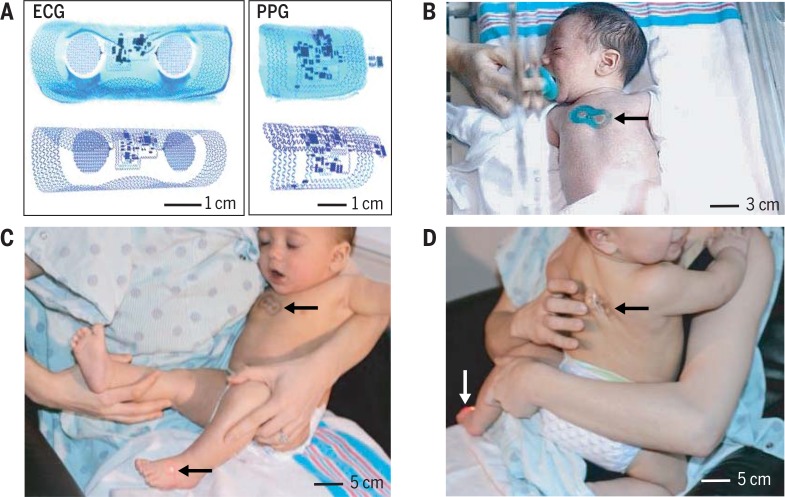
**Wireless, skin-like systems for vital signs monitoring in neonatal intensive care.** (**A**) Images and finite-element modeling results for ECG and PPG devices bent around glass cylinders. (**B**) A neonate with an ECG device on the chest. (**C** and **D**) A mother holding her infant with a PPG device on the foot and an ECG device on the chest (**C**) and on the back (**D**).

Continuous recording and real-time graphical display of vital signs are essential for critical care. Each year in the United States, approximately 300,000 neonates, including a large fraction with exceptionally fragile health due to severe prematurity and very low birth weight (<1500 g), are admitted to neonatal intensive care units (NICUs) (*1*). Existing monitoring systems for the NICU require multiple electrode/sensor interfaces to the skin, with hardwired connections to separately located base units that may be stand-alone or wall-mounted, for heart rate (HR), respiratory rate (RR), temperature, blood oxygenation (SpO_2_), and blood pressure (BP). Although such technologies are essential to clinical care, the associated web of wires complicates even the most basic bedside tasks, such as turning a neonate from prone to supine. This hardware also interferes with emergency clinical interventions and radiological studies, and impedes therapeutic skin-to-skin contact (colloquially known as kangaroo mother care) between parents and their infant. Moreover, the adhesives that couple these wired electrodes to the fragile skin of the neonates are a frequent cause of iatrogenic injuries and subsequent scarring (*2**–**4*).

A fully wireless alternative that eliminates mechanical stresses and potentially reduces injury risk, and that deploys effectively on the full range of gestational ages encountered in the NICU, would represent a substantial advance over the existing standard of care. Although textile-based sensors are of interest, these technologies retain wired connections across the body, and their inability to support an intimate connection to the skin precludes reliable operation at clinical-grade levels of accuracy, particularly with motion (*5**–**7*). Recent advances in materials science and biomedical engineering serve as the basis for devices that have a skin-like form factor. Although such systems can support various types of biophysical measurements of physiological health (*8**–**13*), additional advances are needed to meet the challenging requirements of the NICU, where comprehensive, continuous sensing with wireless functionality, clinical-grade measurement fidelity, and mechanical form factors that eliminate risk of harm to exceptionally fragile neonatal skin are essential.

We have developed a wireless, battery-free vital signs monitoring system that exploits a binodal pair of ultrathin, low-modulus measurement modules, each referred to as an epidermal electronic system (EES), capable of softly and noninvasively interfacing onto neonatal skin. Successful pilot-phase demonstrations on neonates with gestational ages ranging from 28 weeks to full term in two tertiary-level NICUs have established quantitative equivalency to clinical standards.

## Sensor designs, system configurations, and wireless, battery-free modes of operation

**Fig. 1:**
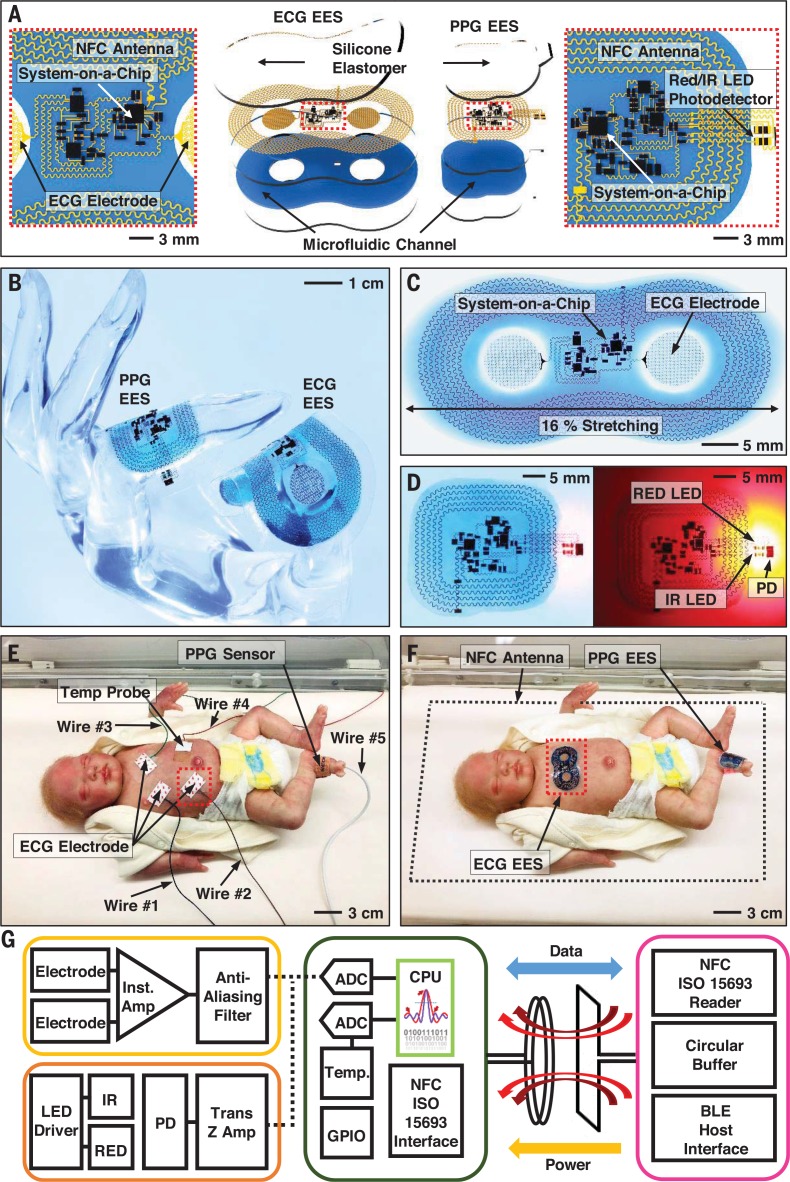
**Schematic illustrations and photographic images of ultrathin, skin-like wireless modules for full vital signs monitoring in the neonatal intensive care unit (NICU) with comparisons to clinical-standard instrumentation**. (**A**) Schematic illustration of wireless, battery-free modules for recording electrocardiogram (ECG) and photoplethysmogram (PPG) data and skin temperature. The ionic liquid in the microfluidic channel contains blue dye for visualization purposes. (**B**) Images of devices draped over the fingers of a life-sized, transparent mannequin hand to illustrate the sizes and physical form factors of these devices. (**C**) Image of an ECG EES stretched uniaxially in the horizontal direction by ~16%. (**D**) Device for capturing PPG data during operation in a lighted and a dark room. PD, photodiode. (**E** and **F**) NICU setting with a life-sized neonate doll configured with conventional measurement hardware (**E**) and with a binodal (chest and foot) deployment of skin-like wireless devices designed to provide the same functionality and measurement fidelity (F). (**G**) Functional block diagram showing analog front end of each EES, components of the NFC SoC including microcontroller, GPIO, and radio interface, with a host reader platform that includes an NFC reader module and a BLE interface with circular buffer.

Figure 1A presents schematic representations of the two wireless EESs. The electronic layer in each EES incorporates a collection of thin, narrow serpentine metal traces (Cu, 50 to 100 mm in width, 5 mm in thickness) that interconnect multiple, chip-scale integrated circuit components. One EES mounts on the chest to record electrocardiograms (ECGs; Fig. 1A, left) through skin-interfaced electrodes that consist of filamentary metal mesh microstructures in fractal geometries; the other mounts on the base of the foot to record photoplethysmograms (PPGs; Fig. 1A, right) by reflection-mode measurements. A microfluidic chamber filled with a nontoxic ionic liquid (1-ethyl-3-methylimidazolium ethyl sulfate) between the electronics and the lower encapsulation layer provides mechanical isolation between the interconnected components and the skin (*14*). A thin film of silicone elastomer encapsulates the top, bottom, and sides to enable operation even when completely immersed in water (fig. S1).

In addition to the electronics, each EES incorporates a magnetic loop antenna (fig. S2) tuned to compliance with near-field communication (NFC) protocols and configured to allow simultaneous wireless data transmission and wireless power delivery through a single link. The low conductivity of the ionic liquid allows stable electrical operation in this radio-frequency (RF) environment (*14*). (See supplementary materials and fig. S3 for details of fabrication methods.) The resulting binodal system captures and continuously transmits ECG, PPG, and skin temperature data from each EES. From these data, HR, heart rate variability (HRV), RR, SpO_2_, and a surrogate of systolic blood pressure (BP) can be extracted.

The images in Fig. 1B show the overall size and ultrathin, soft form factor of these systems. Finite element analysis and experimental results indicate that these devices can bend to radii that aremuch smaller (6.4mmand 5 mm, respectively; fig.S4) thanrequired (>~140mmand>~50mm for the chest and foot, respectively, depending on gestational age) to interface with the chest and the limb of each neonate, without adverse mechanical effects on the device or skin. The electromagnetic properties of both the ECG EES and PPG EES undergo negligible changes when stretched and bent in this manner (figs. S5 and S6). Stretching the ECG EES uniaxially by up to 16% (Fig. 1C) and the PPG EES by up to 13% results in strains in the electronics and antenna structures that remain below the limits for plastic deformation (~0.3%; figs. S7 to S9). Even with 20% stretching, the changes in the inductance, Q factor, and resonant frequency of the antennas are minimal (< 5%) (figs. S10 and S11). Figure 1D shows pictures of a PPG EES with its red LED activated, captured with and without external illumination.

Images in Fig. 1, E and F, compare clinicalstandard technologies to our devices, as deployed on a realistic model of a neonate. Existing systems require a collection of separate electrodes, sensors, and limb-strapped systems paired to base units with hard-wired connections. An ECG requires three adhesive-backed electrodes with adjoining wires to monitor HR, HRV, and RR. Commonly used electrodes for this purpose (e.g., Red Dot, 3M Company) may require additional adhesives that further increase the risk of skin injury. Measurements of SpO_2_ rely on limb-based devices for PPG (e.g., LNCS Neo SpO_2_ sensor, Masimo), typically wrapped around the entire foot, with an additional wired interface. Continuous measurements of skin temperature, necessary to monitor for signs of hypothermia, involve another adhesive-backed sensor (e.g., HNICU-22, DeRoyal) and adjoining wire. Collectively, then, vital signsmonitoring in the conventionalmanner requires at least four electrodes and one limb-deployed device, with five wires for external connection to yield HR, HRV, RR, skin temperature, and SpO_2_.

The block diagram in Fig. 1G summarizes the system architecture and overall wireless operation of our systems. The ECG EES includes two epidermal electrodes, an instrumentation amplifier, analog filters, an inverting amplifier, and a NFC system-on-a-chip (SoC) (fig. S12). The PPG EES includes a pair of small-scale LEDs that emit in the red (640 nm) and infrared (IR) (940 nm), a photodiode, LED drivers, an external power circuit, analog filters, an inverting amplifier, and aNFC SoC (fig. S13). A 14-bit analog-to-digital converter (ADC) operating at a sampling frequency of 200 Hz digitizes the signals captured by each module. The RF loop antennas in both the ECG EES and PPG EES serve dual purposes in power transfer and in data communication.

The standard NFC protocol at 13.56 MHz supports only low-speed, low-fidelity applications such as contactless payments and wireless identification (*15*); thus, substantial modification in both the transponder and host reader systems at ISO15693 was required to support data transfer rates sufficient for NICU monitoring (hundreds of Hz). The results enable continuous streaming of data at rates of up to 800 bytes/s with dual channels, which is orders of magnitude larger than those previously achieved in NFC sensors (*15**–**17*). A key to realizing such high rates is in minimizing the overhead associated with transfer by packaging data into six blocks (24 bytes) in a circular buffer. Reading occurs with a NFC host interfaced to a microcontroller in a Bluetooth Low Energy (BLE) system configured with the customized circular buffer decoding routine (fig. S14). The primary antenna connects to the host system for simultaneous transfer of RF power to the ECG EES and the PPG EES. Operation is possible at vertical distances of up to 25 cm, through biological tissues, bedding, blankets, padded mattresses,wires, sensors, and other materials found in NICU incubators, for full-coverage wireless operation in a typical scenario (fig. S15). BLE radio transmission then allows transfer of data to a personal computer, tablet computer, or smartphone with a range of up to 20 m. Connections to central monitoring systems in the hospital can then be established in a straightforward manner.

## Low-modulus mechanics, soft interface adhesion, and implications for neonatal skin safety

The essential mechanics of these systems decrease risks for skin injury relative to existing clinical standards. The global incidence of skin breakdown in hospitalized neonates ranges between 31 and 45%, with medical devices and associated adhesives being a major iatrogenic cause (*3* *,* *7*). Additionally, pressure-related skin injuries occur in 26% of hospitalized infants less than 3 months of age (*7*) with 80% directly related to medical devices, where PPG modules are the most common culprit (*18*). By age 7,more than 90% of children born preterm (<30 weeks gestation) and previously cared for in the NICU exhibit residual scars secondary to monitoring probes, adhesives, and invasive medical interventions (*4*). Premature neonates are particularly high-risk given that their epidermis and dermis are only 40 to 60% as thick as adult skin, with incomplete cornification, decreased mechanical strength, and greater propensity to scar (*19*). Although all neonatal skin is susceptible to iatrogenic injury, premature neonates are especially vulnerable. At 24 to 30 weeks gestation, the epidermis is 60% as thick as it is at 36 to 40 weeks (*20*), and it is considerably more fragile. As a result, removal of adhesives necessary for securing medical equipment poses a greater risk with greater prematurity, where up to 15% of a neonate’s total skin surface area can be traumatized daily (*21*).

**Fig. 2:**
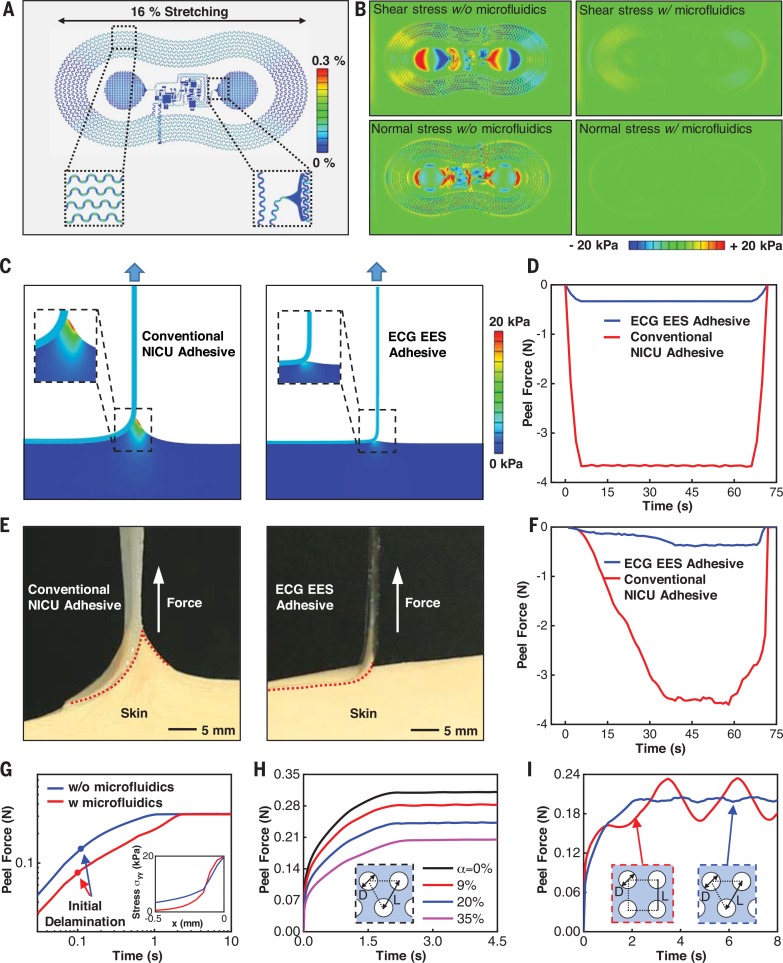
**Fundamental aspects of mechanical stresses and soft adhesion at the interface with the skin. (A)** Simulation results for the deformed geometry and distribution of strain in the copper layer of an ECG EES during uniaxial stretch (~16%). **(B)** Simulation results for the distribution of shear and normal stresses at the interface between an ECG EES and underlying skin during deformation for devices without (left) and with (right) the microfluidic channel. Stresses in the latter case are less than ~20 kPa, the threshold of skin sensation. **(C)** Simulation results for the distribution of von Mises stress on the skin due to peeling of a conventional NICU adhesive (left) and the ECG EES adhesive (right). **(D)** Simulation result for the time dependence of the peel force during removal of a conventional NICU adhesive and the ECG EES adhesive from the skin. **(E)** Images that highlight experimental studies of peeling of a conventional NICU adhesive (left) and the ECG EES adhesive (right) from the skin of a healthy adult. **(F)** Experimental measurement of the time dependence of the peel force during removal of a conventional NICU adhesive and the ECG EES adhesive from the skin. **(G)** Simulation results that highlight the role of the microfluidic channel in the peel force associated with removal of an ECG EES from the skin, with emphasis on the initial, non–steady-state regime during peel initiation. The circles denote the instants of initial delamination, when the interfacial cohesive strength is reached. The inset shows the normal stress distribution, ᵟ_yy_, along the interface at the instant of initial delamination, where its peak is the cohesive strength. **(H)** The computed peel force as a function of time for an EES adhesive with a triangular pattern of small holes (diameter *D* = 200 µm) on the skin.The hole area fraction $\sqrt{3\text{π}D^{2}/6L^{2}}$.**(I)** The computed peel force as a function of time for triangular and square patterns of large holes (diameter *D* = 1 mm) with the hole area fraction a = 35%, where α = πD_2_ /4L^2^ and $\sqrt{3\text{π}D^{2}/6L^{2}}$ for square and triangular patterns, respectively.

The inherently thin, soft mechanical properties of the sensors (Fig. 1A) reported here allow for adhesion via van derWaals forces alone. Effective moduli in the range of 200 to 300 kPa (Fig. 2A) lead to minimal normal and shear stresses at the skin interface associated with natural motions of the neonate. The mechanical decoupling afforded by the microfluidic channel decreases these stresses by up to a factor of 2.5 (Fig. 2B) relative to otherwise similar designs without the microfluidics. Experimental and theoretical studies reveal additional fundamental aspects of the soft mechanics and adhesion in these systems. Simulations that use the cohesive zone model (fig. S16) allow quantitative examination of the physics associated with removal of conventional adhesives (e.g., Argyle Hydrogel Adhesive Baby Tape Strips, Covidien) and EES devices (modeled as an effective medium; Fig. 2, C and D) from surfaces with mechanical properties reflective of neonatal skin. The differences between the magnitudes of deformations induced in the skin, at identical peel forces, are notable (Fig. 2C). The forces at steady-state peeling rates are different by approximately a factor of ~10 (Fig. 2D), with reduction in the maximum von Mises stress on the skin by a factor of 4.3. Experimental testing on adult skin (Fig. 2, E and F) shows similar behavior, including a substantial reduction in peel force (~1000%; fig. S17) of an EES relative to that of a traditional adhesive. Analysis of these experimental results defines the adhesion energy at the interface between the EES and skin: *G* = 16 N/m.

The presence of themicrofluidic channel (fig. S18) serves an important role in determining the adhesion properties of the EES, as shown in Fig. 2G. At steady state (>2 s), the peel forces (*F*) with and without the microfluidics are approximately the same, consistent with a scaling relationship that depends only on G and the width of the device, *W*, as *F* = *G* × *W*(*[Bibr cit0022]22*). In other words, the adhesion energy defines the steady-state peeling force. At the initiation of peeling, however, in the non–steady-state regime when the forces on the skin are most important, the cohesive strength determines the force. Specifically, the interface starts to delaminate when the normal stress reaches ~20 kPa (Fig. 2G, inset). Themicrofluidic channel reduces the effectivemodulus of the EES and, as a consequence, increases the ability of the device to deform under applied force. The consequent reduction in the size of the cohesive zone at the delamination front (fig. S19) decreases the peel force for the same peak stress (cohesive strength).

Further reductions can be achieved by the addition of perforations through the open regions of the EES platform, as shown in fig. S20 for different patterns of holes. Figure 2H highlights the peel force, the primary driver of epidermal stripping in fragile neonatal skin (*3*), as a function of time during peeling for a regular triangular pattern of holes (diameter *D* = 200 μm). The force scales with 1 – α (fig. S21)—that is, the area of contact between the EES and the skin. This scaling also applies to other patterns of holes (e.g., figs. S22 and S23 for square patterns without and with 45° rotation, respectively). For sufficiently small holes (e.g., 200 mm; see figs. S24 to S28), the relation between the peel force and time depends only on 1 – α and is approximately independent of pattern. Oscillations in the force only appear for holes larger than the characteristic size of the cohesive zone (~500 mm, as in Fig. 2I). An optimized approach to reducing interface stresses and peel forces, therefore, combines microfluidic channel structures with small perforation holes, the latter of which can be naturally accommodated within the open network designs characteristic of epidermal electronics (Fig. 1A).

## Compatibility with medical imaging techniques used in the NICU

**Fig. 3:**
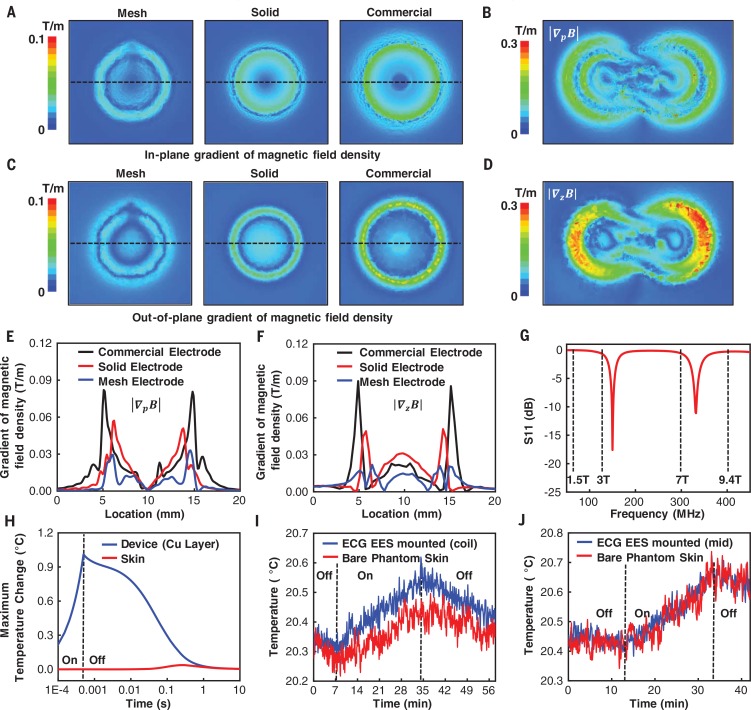
**Theoretical and experimental aspects of radiolucency. (A)** Computational results for the distributions of the in-plane gradient of the magnetic field density associated with a mesh electrode as in Fig. 1A (left), a solid electrode (no mesh; center), and a commercial NICU electrode (right) for conditions associated with an MRI scan at 128 MHz. **(B)** Calculated in-plane gradients of the magnetic field density associated with a complete ECG EES at 128 MHz. **(C)** Distributions of the out-ofplane gradient of the magnetic field density associated with a mesh electrode, a solid electrode, and a commercial NICU electrode for conditions associated with an MRI scan at 128 MHz. **(D)** The out-of-plane gradients of magnetic field density induced on the ECG EES at 128 MHz. **(E)** The in-plane gradient of the magnetic field density evaluated along the horizontal dashed lines in (A). **(F)** The out-of-plane gradient of the magnetic field density along the horizontal dashed lines in (C). **(G)** S11 parameter of the ECG EEG as a function of frequency. The vertical dashed lines indicate operating frequencies of 1.5-T, 3-T, 7-T, and 9.4-T MRI scanners at 64 MHz, 128 MHz, 298 MHz, and 400 MHz, respectively. **(H)** Computational results for the maximum change in temperature of an ECG EES on skin during an MRI scan. **(I)** Temperature changes collected using two fiber-optic thermometers located at the interface between an ECG EES (at the loop antenna, coil) and a piece of phantom skin (blue) and on the surface of the phantom skin (red) during MRI scanning (3-T MRI). **(J)** Temperature changes collected by two fiber-optic thermometers at the interface between an ECG EES (at one of the mesh electrodes) and a piece of phantom skin (blue) and on the surface of the phantom skin (red) during MRI scanning (3-T MRI).

Magnetic resonance imaging (MRI) is essential in the NICU because of its ability to deliver precise assessment ofwhitematter, graymatter, and posterior fossa abnormalities with functional capabilities that exceed those of ultrasound (*23* *,* *24*). The EES platforms exploit designs that minimize disturbances in the time-dependent magnetic fields associated with MRI scanning, thereby reducing distortions and shadowing artifacts in the final images and eliminating any parasitic heating from magnetically induced eddy currents. Calculations of the gradients of the magnetic field density near electrodes with different structures (mesh, solid, and commercial electrodes, see fig. S29) on biological tissues in a 3-T MRI scanner reveal the underlying effects. The results show that mesh electrodes induce the weakest disturbance to the magnetic field among mesh (layout of Fig. 1A), solid (i.e., no mesh), and commercial electrodes with similar overall sizes and geometries (Fig. 3, A and C) for both the in-plane |∇_p_B| and out-of-plane |∇_z_B| gradient of the magnetic field density. The maximum value of |∇_p_B| for the mesh electrode is smaller than that of the commercial electrode by a factor of ~3 (Fig. 3E), whereas |∇_z_B| is smaller by a factor of 4 (Fig. 3F). The mesh design also has advantages in its soft, flexible mechanics and associated benefits in interfacial stresses and adhesion, as described previously. Additional simulations guide selection of designs that ensure that the resonant frequencies of the EES have no overlap with the working frequencies of typical MRI scanners (64 MHz, 128 MHz, 298 MHz, and 400 MHz for 1.5-T, 3-T, 7-T, and 9.4-T MRI scanners, respectively; Fig. 3G), thereby avoiding large gradients of the magnetic field density (Fig. 3, B and D, and figs. S30 and S31). Similar simulations for the PPG EES indicate gradients of the magnetic field density that are smaller than those for the ECG EES (fig. S32). These features allow the devices to remain in place on neonates undergoing MRI imaging to mitigate the risks of injury and complications with removal and re-adhesion.

**Fig. 4:**
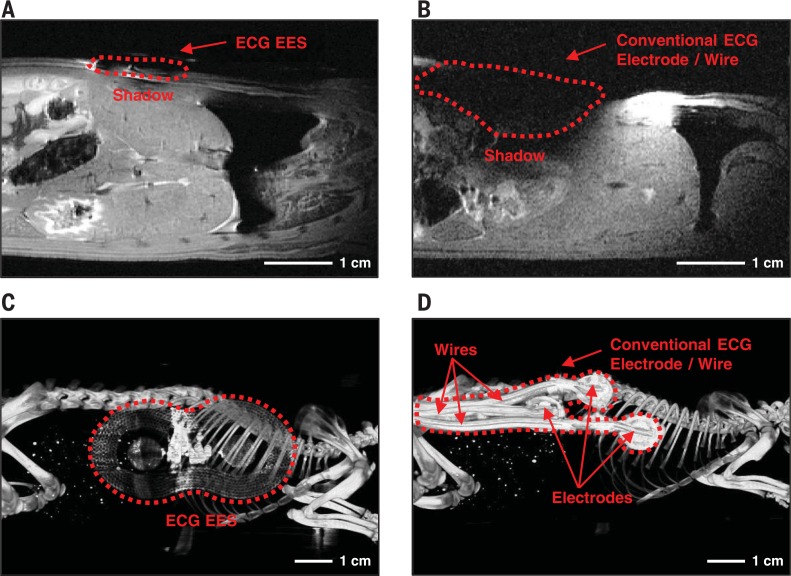
**Visualization of radiolucent properties through medical imaging. (A)** A coronal MRI image collected from the mid-dorsum of a rat cadaver with an ECG EES mounted on the skin. **(B)** A coronal MRI image collected from the mid-dorsum of a rat cadaver with conventional ECG leads mounted on the skin. **(C)** An x-ray image collected from the right flank of a rat cadaver with an ECG EES mounted on the skin. **(D)** An x-ray image collected from the right flank of a rat cadaver with conventional ECG leads mounted on the skin.

Experiment and simulation results also yield information on parasitic heating during an MRI scan. Full three-dimensional multi-physicsmodeling shows that, at the end of a single scan for 0.5 ms, the copper layer of an ECG EES undergoes heating by only 1°C (Fig. 3H). The resultant maximum temperature change at the skin interface is 0.04°C, far below the threshold for sensation, due to the insulating effects of the polydimethylsiloxane (PDMS) and the microfluidic channel. The maximum change in temperature occurs ~0.24 s after initiating the scan (Fig. 3H and fig. S33). This time scale is on the same order as that for heat conduction (0.1 s) in the microfluidic channel (fig. S33). Experimental measurements support these findings. Figure 3I shows the change of temperature during an MRI scan (3-T MAGNETOM Prisma, Siemens Healthineers),measured on a sample of phantom skin (designed to match the conductivity and dielectric constant of tissue at 33 MHz) at a location underneath the ECG EES near the loop antenna and adjacent to the device. The results show a temperature difference of ~0.1°C. Figure 3J presents measurements in the middle region of the ECG EES where the values of |∇_z_B| and |∇_p_B| are comparable to those for bare phantom skin. Simulation results for the PPG EES suggest even smaller changes in temperature than those for the ECG EES (fig. S34). Additional testing with another MRI system (9.4-T Bruker Biospec MRI system, Bruker BioSpin Corporation) and an ECG EES placed over cadaveric rat tissue (Fig. 4A) shows no observable magnetically induced displacement forces or torques and no measurable changes in temperature. The results meet FDA requirements for an “MRI-safe” label for medical devices (*25*). Imaging results indicate that the ECG EES causes less shadowing and image distortion compared to a conventional NICU ECG electrode (Fig. 4B).

The EES eliminates radio-opaque wires, thereby improving evaluation by x-ray imaging, a modality required for 90% of low–birth weight neonates (*26*). Experimental results show that an ECG EES placed over the same tissue in a rodent model imaged using a computed tomography/x-ray system (nanoScan PET/CT, Mediso) exhibits improved radiolucency in comparison to standard ECG electrodes and wires (Fig. 4, C and D). The optical transparency of the silicone and the open mesh designs of the electronics and antenna structures also provide direct visual access to the skin and tissue beneath the sensor (fig. S35), thereby obviating the need to remove the sensor tomonitor the underlying skin for signs of infection or irritation.

## Real-time measurements, in-sensor analytics, and data transmission

**Fig. 5:**
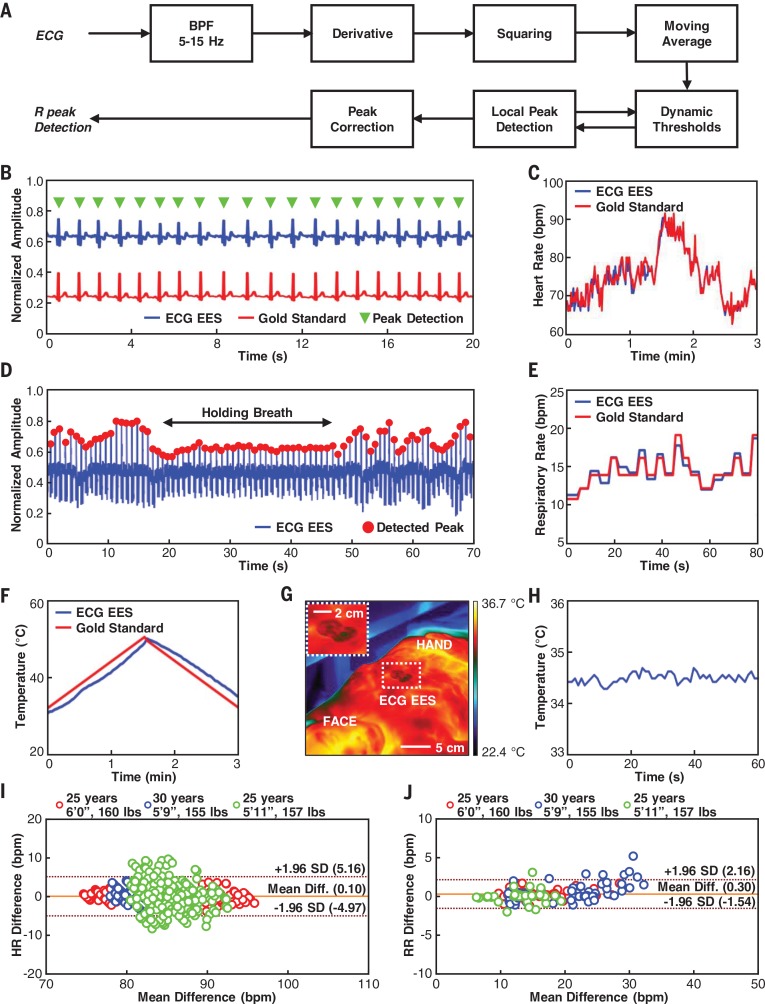
**Operational characteristics of the ECG EES. (A)** Block diagram of in-sensor analytics for peak detection from ECG waveforms. **(B)** ECG signals acquired simultaneously from an ECG EES (blue) and a gold standard (red), with detected peaks (green). **(C)** Comparison of heart rate determined using data from the ECG EES and a gold standard. **(D)** Respiration rate extracted from oscillations of the amplitudes of peaks extracted from the ECG waveforms. **(E)** Comparison of respiration rate determined using data from the ECG EES and manual count by a physician. **(F)** Comparison of skin temperature determined by the ECG EES and a gold-standard thermometer. **(G)** Thermal image of the chest collected using an IR camera. **(H)** Temperature wirelessly measured using an ECG EES. **(I)** Bland-Altman plot for heart rate collected from three healthy adults using an ECG EES and a clinical-standard system. **(J)** Bland-Altman plot for respiratory rate collected from three healthy adults using an ECG EES and a clinical-standard system.

Exploiting this collection of attractive electronic, mechanical, and radiolucent properties for practical use in a NICU environment requires insensor processing and data analytics to reduce bandwidth requirements on wireless transmission and to ensure operational robustness. For example, computational facilities on the NFC SoC of the ECG EES can support a streamlined version of the Pan-Tompkins algorithm (*27*) for accurate, on-board analysis of the QRS complex of ECG signals in real-time to yield HR and HRV on a beat-to-beat basis. Figure 5A summarizes an approach that starts with digital bandpass filtering (*f*_c1_ = 5 Hz, *f*_c2_ = 15 Hz) to attenuate the noise. Differentiating and squaring the resulting data yields the slope of QRS peaks and prevents false peak detection associated with the T wave. Applying a moving average and a dynamic threshold identifies a running estimate of the R peak and the magnitude of the noise. Automatic adjustments of the threshold rely on these estimates for the preceding beat cycle (fig. S36). The R-to-R intervals determined in this way yield the instantaneous HR. Simultaneous recordings obtained using a clinical-standard system, henceforth referred to as “gold standard” data, validate the ECGEESmodule hardware and in-sensor analytics via measurements on a healthy adult volunteer (Fig. 5, B and C). The ECG signals and computed HR values from these two platforms show nomeasurable differences. Periodic modulations of the amplitude of the R peak define the RR (Fig. 5D), which also agrees with the gold standard (visual counting by a physician in this case; Fig. 5E).

Measurements of skin temperature rely on sensors internal to the NFC SoC in each EES, where transmission at a sampling frequency of 1 Hz is sufficient for monitoring purposes. The low thermal mass of the EES and the small thickness of the substrate layer (PDMS; 50 mm in thickness) that separates the SoC from the skin ensure fast thermal response times and excellent thermal coupling, respectively. Comparisons against readings from a thermometer (Fisherbrand 13202376, Fisher Scientific) serve as means to calibrate the sensor (Fig. 5F) via testing in a water bath (fig. S37). Thermal images captured with an IR camera (FLIR A325SC, FLIR Systems) during operation indicate negligible heating associated with the electronics or the antenna structures (Fig. 5G). Figure 5H shows temperature readings from the ECG EES for 60 s. Comparison tests of this system against FDAcleared monitoring equipment (Dash 3000, GE Healthcare) on healthy adult volunteers (*n* = 3) show excellent agreement for HR (mean difference = 0.1 bpm, SD = 2.55 bpm) and RR (mean difference = 0.3 bpm, SD = 0.95 bpm) as shown in Fig. 5, I and J, respectively.

**Fig. 6:**
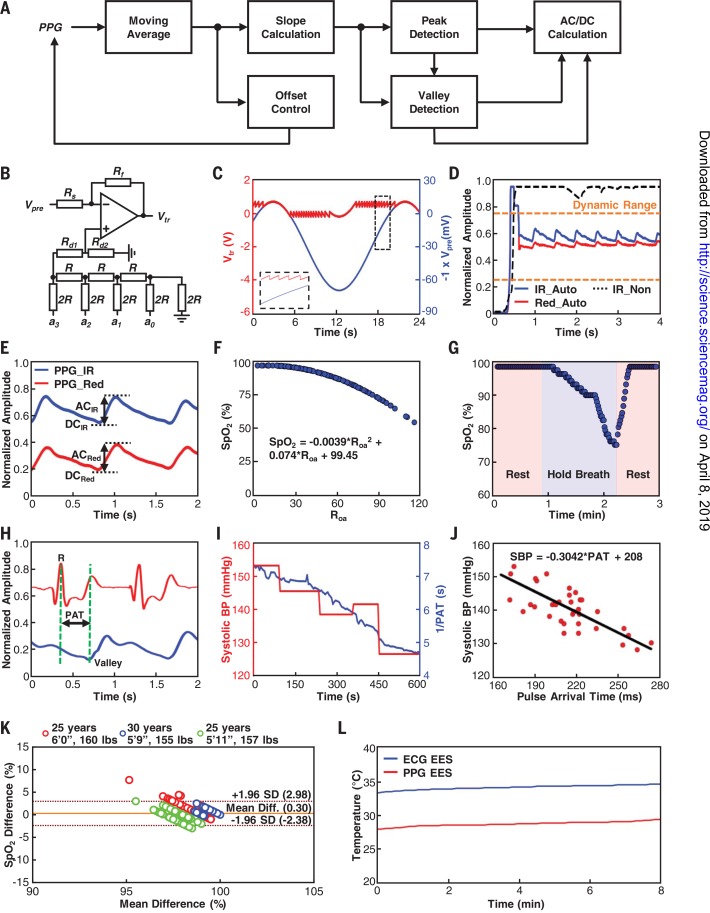
**Operational characteristics of the PPG EES. (A)** Block diagram of in-sensor analytics for detection of peaks and valleys from PPG waveforms and for dynamic baseline control. **(B)** A circuit diagram with GPIO-enabled baseline control scheme. **(C)** Demonstration of dynamic baseline level control with a sinusoidal input (blue) and corresponding output changes (red). **(D)** Demonstration of operation of a PPG EES with (blue and red) and without (black dashed line) dynamic baseline control. Analytics on baseline level serves as an input to a control system that combines a GPIO port on the NFC SoC with an offset to ensure that the signal input to the ADC lies within its dynamic range (orange dashed lines). **(E)** Convention for calculating direct and alternating components of PPG waveforms collected in the red and IR, for purposes of calculating SpO_2_. **(F)** Empirical formula for SpO_2_ calculation using R_oa_ based on comparison to a commercial pulse oximeter. **(G)** SpO_2_ determined using in-sensor analytics during a period of rest followed by a breath hold and then another period of rest. **(H)** Convention for measuring pulse arrival time (PAT) from R-peaks in the ECG waveforms and valleys in the PPG waveforms. **(I)** Values of 1/PAT acquired using an ECG EES and a PPG EES versus systolic BP data acquired using a cuff monitor. **(J)** Correlation curve between PAT and systolic BP with linear fit. **(K)** Bland-Altman plot for SpO_2_ collected from three adults using a PPG EES and a clinicalstandard system. **(L)** Temperature plot showing the capability for measuring differential skin temperatures between the torso and the foot using an ECG EES and a PPG EES.

The PPG EES relies on similar NFC protocols, but with in-sensor analytic methods that not only reduce requirements on transmission bandwidth but also provide, when used in conjunction with adaptive circuits, crucial functionality for stable operation. Specifically, the processing in this case enables (i) dynamic baseline control to ensure that the input to the ADC on the NFC SoC lies within the linear response range and (ii) real-time calculation of SpO_2_ from the PPG traces (Fig. 6A). Here, the processing begins with application of a moving-average filter to the photodetector response from the red and IR LEDs. When the larger of these two averaged PPG amplitudes (typically that associated with the IR response) lies outside of a range that is optimal for the ADC (0.25 to 0.7 V), a programmable difference amplifier with voltage dividers at V+ dynamically adjusts the baseline level. The circuit shown in Fig. 6B demonstrates the operation where the governing equation is

$$V\text{tr}=-\frac{R_{\text{f}}}{R_{\text{s}}}V\text{pre+}\left(\frac{1+R_{\text{f}}}{R_{\text{s}}}\right)V+$$ 1

where *V*_tr_ is the voltage output of the amplifier, *V*_pre_ is the voltage of the input signal, *R*_s_ is the input resistance, and *R*_f_ is the feedback resistance. The voltage divider at V+ with resistors *R*_d1_ and Rd2 is governed by the following equation with *V*_ref_ = 1.8 V:

$$V+=\frac{R_{\text{d2}}V_{\text{ref}}}{R+R_{\text{d2}}+R_{\text{d}1}}\left(\frac{a_{0}}{16}+\frac{a_{1}}{8}+\frac{a_{2}}{4}+\frac{a_{3}}{2}\right)$$ 2

Sixteen different baseline states can be accessed via activation of binary values from four generalpurpose input-output pins (GPIOs; *a*_0_, *a*_1_, *a*_2_, *a*_3_) on the SoC (fig. S38), applied through an R-2R resistor ladder. Figure 6C shows dynamic control of the output voltage Vtr of a sinusoidal input signal (frequency = 50mHz, amplitude = 40mV, *V*_offset_ = –30 mV). Starting with the default setting of the GPIO ports (*a*_0_, *a*_1_, *a*_2_, *a*_3_; all high, or 1111), the baseline level automatically adjusts to lower levels associated as the value of *V*_tr_ drifts above the upper boundary of the specified voltage range, and vice versa as *V*_tr_ falls below the lower boundary. The result maintains *V*_tr_ in the allowed range. Figure 6D summarizes the operation in an actual PPG recording. Without this type of real-time, in-sensor processing (IR_Non in Fig. 6D), robust operation would be impossible: PPG signals would quickly drift outside of the narrow operating range of the ADC as a result of patient-to-patient variations in skin pigmentation and unavoidable, time-dependent fluctuations in optical scattering that result from micromotions relative to underlying blood vessels and subdermal structures (*28*).

Calculating SpO_2_ involves determining the ratio (*R*_oa_) between the alternating and direct components of the PPG signals according to

$$R_{\text{oa}}=\frac{{\text{AC}}_{\text{RED}}/{\text{DC}}_{\text{RED}}}{{\text{AC}}_{\text{IR}}/{\text{DC}}_{\text{IR}}}$$ 3

for data from the red and IR LEDs (Fig. 6E). An empirical calibration formula determined by comparison to an FDA-cleared fingertip oximeter measurement (MightySat Fingertip Oximeter, Masimo) converts the *R*_oa_ to SpO_2_ (Fig. 6F). Timedependent variations of SpO_2_ determined in this manner appear in Fig. 6G with demonstration in a decreasewith a breath hold in an adult volunteer.

The time-synchronized outputs from the ECG EES and the PPG EES allow for determination of advanced physiological parameters that are of high clinical value but not regularly collected in routine practice inNICUs. A key example is the measurement of pulse arrival time (PAT), defined by the time lapse between the maximum fiducial point in the ECG signal (R peak) and the corresponding minimal fiducial point in the PPG signal at valley as in Fig. 6H, as a direct correlate to systolic blood pressure (*29* *,* *30*). Blood pressure is an essential physiological marker of perfusion, autonomic function, and vascular tone for critically ill newborns (*31*). Cuff-based blood pressure measurements with sphygmomanometers fail to provide continuous measurement, overestimate blood pressure in premature neonates (*32*), and pose a direct risk for pressure-related injuries (*33*). Although arterial lines offer a continuous measurement of blood pressure in neonates, these invasive interventions can cause thrombosis, hematomas, infection, and even death (*34*). Thus, the ability to capture PAT noninvasively and continuously would be of high clinical value in the NICU, with prior reports providing evidence that PAT correlates with blood pressure in infants (*35**–**37*).

The Moens-Korteweg equation provides a linear relationship between PAT and BP (*38* *,* *39*). Measurements of 1/PAT performed in processing of ECG and PPG data in the host (fig. S39), together with corresponding values of systolic BP captured using a sphygmomanometer on a healthy adult during a period of rest after exercising (running at 6 miles per hour for 15 min), exhibit the expected linear relationship (Fig. 6I). A calibration plot with a linear fit is shown in Fig. 6J. The binodal configuration of the system naturally yields not only a surrogate marker of BP but also temperatures at two different locations (trunk and limb), to improve monitoring for hypothermia and provide a noninvasive method to track peripheral perfusion. In current clinical practice, measurements of skin temperature are typically limited to a single body location because of the need to minimize wired connections and adhesive interfaces to the skin. Comparison tests of this system against FDA-cleared monitoring equipment (Dash 3000, GE Healthcare) on healthy adult volunteers (*n* = 3) show excellent agreement for SpO_2_ (mean difference = 0.3%, SD = 1.37%) as shown in Fig. 6K. Figure 6L illustrates the ability of an ECG EES and a PPG EES to capture differential skin temperature between the torso and peripheral limbs.

## Pilot studies in neonatal intensive care units and validation against clinical standards

**Fig. 7:**
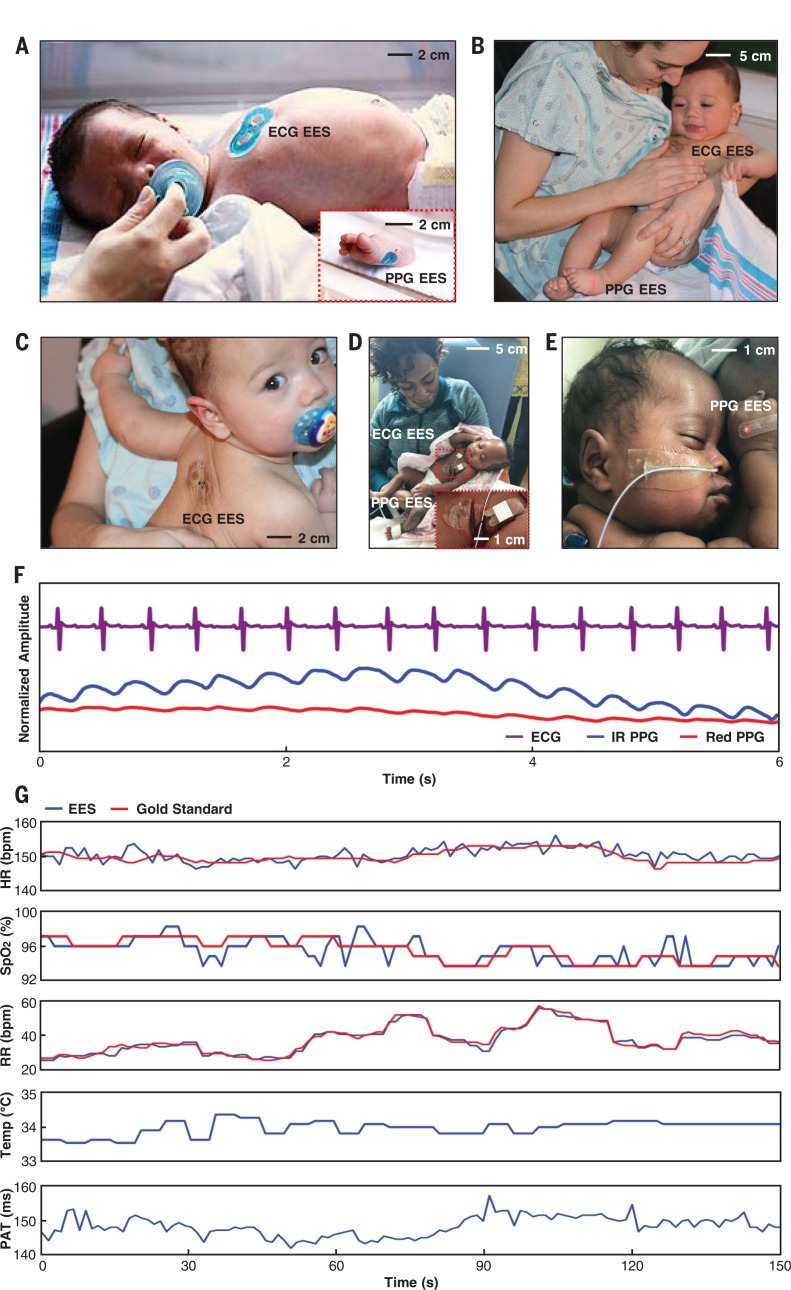
**Data collection from neonates in clinical and home settings. (A)** A healthy term neonate with an ECG EES and a PPG EES on the chest and the bottom of the foot, respectively. (**B** and **C**) A mother holding a healthy term neonate showing skin-to-skin interaction with an ECG EES mounted on the chest (B) and an ECG EES mounted on the back (C). **(D)** A mother holding her neonate in the NICU; the inset is a magnified view of the ECG EES. **(E)** A neonate in the NICU with a PPG EES mounted on an alternative location on the hand. **(F)** Representative ECG and PPG waveforms acquired in this manner from a healthy term neonate. **(G)** Comparison of vital signs calculated from the ECG EES and a gold standard. Temperature and PAT data are displayed without reference data because these measurements are only periodically acquired with conventional devices.

Preliminary testing of the EES system in both healthy neonates and premature infants in two tertiary-level NICUs demonstrates feasibility and measurement validity. Shown in Fig. 7, A to C, is a healthy term neonate with an ECG EES and a PPG EES mounted on the chest and the foot, respectively, where van der Waals forces govern themechanical interface to the skin, with minimal mechanical, mass, or thermal load (Fig. 7A; gestational age, 38 weeks 3 days; birth weight 2.75 kg). The silicone encapsulation also enables reliable operation of the systems when completely immersed in water (fig. S1), thereby supporting compatibility with NICU incubators commonly set at humidity above 80% to maintain temperature homeostasis and prevent dehydration in premature neonates (*40*).

Figure 7B illustrates the use in a mode that facilitates physical contact between parent and neonate, which is difficult to replicate with hardwired conventional systems. Figure 7C shows an alternative mounting location, where the ECG EES resides on the back of the neonate to facilitate chest-to-chest skin interaction, while highlighting the intimate contact with the skin, even while naturally deformed and wrinkled. In Fig. 7, D and E, the sensor system is on a neonate admitted in the NICU, highlighting intimate contact of the ECG EES to the skin, even with motion and position adjustment. An additional example of skin-to-skin contact in a chest-tochest position is shown in Fig. 7E, with the PPG EES on the upper limb to illustrate another option for placement. Representative results of continuous recordings are shown in Fig. 7F for the neonate in Fig. 7A. Calculated HR, SpO_2_, and RR from the experimental system are consistent with measurements obtained from gold-standard equipment operating concomitantly (Intellivue MX800, Philips). The temperature and PAT data appear alone because of the absence of a comparator system (Fig. 7G).

**Fig. 8:**
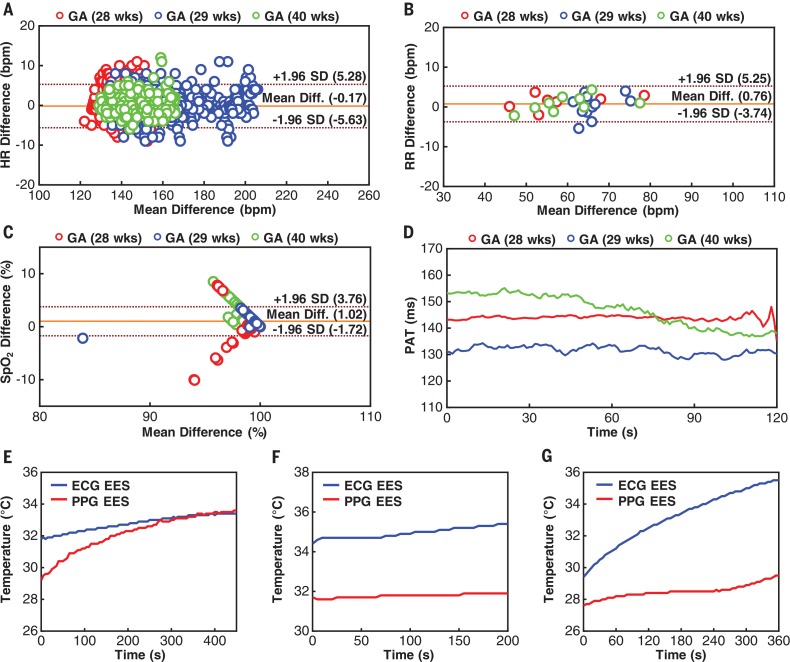
**Data collection from neonates in operating neonatal intensive care units. (A)** Bland-Altman plot for HR using data from an ECG EES and a clinical standard. **(B)** Bland-Altman plot for RR using data from an ECG EES and a clinical standard. **(C)** Bland-Altman plot for SpO_2_ using data from a PPG EES and a clinical standard. **(D)** Representative results for PAT determined using combined data from an ECG EES and a PPG EES. (**E** to **G**) Differential temperature data collected from an ECG EES and a PPG EES for three recruited neonates with gestational ages of 28 weeks (E), 29 weeks **(**F**)**, and 40 weeks (G). The other data presented here were collected from this same set of neonates. See fig. S40 for additional data.

Further validation studies involve deployment on neonates (*n* = 3) ranging in gestational age from 28 to 40 weeks admitted to the NICU with synchronous, concomitant measurements from standard-of-care monitors (Intellivue MX800, Philips; table S1). The resultant data show strong agreement in HR, RR, and SpO_2_ (Fig. 8, A to C). Themeandifference is –0.17 beats per minute for HR, 0.76 breaths per minute for respiratory rate, and 1.02% for SpO_2_. Advanced physiological parameters such as PAT and continuous differential skin temperature are also shown (Fig. 8, D to G). Additional studies in *n* = 18 neonates admitted to the NICU with gestational ages as low as 28 weeks and weights as low as 1470 g, using related device platforms with onboard power supplies to facilitate testing, further validate the operation and applicability across a larger cohort of subjects (fig. S40 and table S1) with similar performance relative to gold-standard monitoring equipment (Intellivue MX800, Philips).

Beyond efficacy and safety, the eventual diffusion of medical technologies depends on economic considerations. Table S2 outlines the cost structures associated with all aspects of device construction, including components, fabrication processing fees, and encapsulation materials. The results suggest costs (ECG EES or PPG EES) of less than $20 USD per unit at scaled production. Full compatibility with autoclave sterilization (2540E,Heidolph) enables safe reuse (fig. S41) and further improved economics, with potential to facilitate deployment in low- and middle-income countries in the context of global health.

## Conclusion

The results presented here represent preliminary feasibility testing and validation of this system for use in NICUs. Comprehensive clinical studies, which are ongoing, will yield additional supporting data to verify the measurements across an increased range of age groups and ethnic backgrounds. These findings will also accelerate efforts to address any remaining challenges, including those potentially related to nursing acceptance, compatibility with legacy monitoring systems, and device sterilization for reuse. For clinical work, additional testing will assess the utility of these platforms in other clinical indications, including those associatedwith subjects who have altered skin barrier function (e.g., burn victims or patients with epidermolysis bullosa).

The results reported here follow from a collection of advances in engineering science to establish the basis for a wireless, skin-like technology that not only reproduces comprehensive vital signsmonitoring capabilities currently provided by invasive, wired systems but also adds multipoint sensing of temperature and continuous tracking of blood pressure. These sensors explicitly address the needs of the NICU because of their high mechanical compliance and noninvasive skin adhesive interface, their water resistance, and their compatibility with essential medical imaging and inspection. In addition to advanced capabilities in monitoring, the skin-like profiles and fully wireless operational modes offer direct therapeutic value by reducing the barriers for skin-to-skin contact between parent and child. Further clinical validation and testing may lead to broad adoption in both high-resource and lowresource settings.

## Methods

### Fabrication

The fabrication involved a combination of semiconductor processing steps, lamination procedures, transfer printing processes, and chip placement and solder bonding. Addition of a thin PDMS layer bonded around the perimeter of the device and the electrodes allowed filling with an ionic liquid using a syringe to form the microfluidic channel. A coating of a soft silicone material on the bottom layer provides a light adhesive surface. Further details are in fig. S3.

### Sensor assessment

A primary antenna (32 cm × 34 cm; fig. S15) is connected to the host system, allowing for simultaneous transfer of RF power to the ECG EES and the PPG EES. The low current consumption of these platforms (up to 450 µA and 5 mA as peak current, respectively) can be satisfied by RF power [4 W; compliant to the Federal Communications Commission (FCC) 47 CFR Part 15 and EN 50364 standard for human exposure] at vertical distances of up to 25 cm through biological tissues, bedding, blankets, padded mattresses, wires, sensors, and other materials found in NICU incubators, and across lateral areas of 32 cm × 34 cm, for full-coverage wireless operation in a typical incubator. Computational work verifies that operation falls within guidelines outlined by the FCC (47 CFR Part 1.1310 and 15) and the FDA in terms of both the specific absorbed radiation and the maximum permissible exposure, with values that are lower than limits for various cases considered by roughly a factor of 10 (figs. S43 to S48).

### Clinical testing

All subject participation was fully voluntary with informed consent obtained from at least one parent. The research protocol was approved by Northwestern University’s Institutional Review Board and the Ann & Robert H. Lurie Children’s Hospital of Chicago (STU00202449/IRB 2016-2) andregisteredonClinicalTrials.gov (NCT02865070). After required initial testing in healthy newborns, the protocol stipulated testing to a limit of 5 min in synchronywith existingNICUmonitoring equipment and only in neonates of adjusted gestational ages above 30weeks with our experimental sensors. Upon successful demonstration of sensor operation and the absence of adverse events, the institutional review board enabled testing in lower gestational age ranges of 30 weeks or less. The placement of the sensors was performed by research staff and/ or NICU-trained nurses. The antenna was preembedded within existing NICU incubators. Sensors were placed on the skin without skin preparation for the neonate thereafter. Data were transmitted, collected, and stored for further data analysis on a tablet PC (Surface Pro 4, Microsoft).

## Data and materials availability

All data are available in the manuscript or the supplementary materials.

## SUPPLEMENTARY MATERIALS

www.sciencemag.org/content/363/6430/aau0780/suppl/DC1

Tables S1 to S4

References (*41–48*)
