# Supplementary material for: Binodal, wireless epidermal electronic systems with in-sensor analytics for neonatal intensive care
Source: Science. 2019 Mar 1;363(6430):eaau0780. doi: 10.1126/science.aau0780 (PMC6510306; doi:10.1126/science.aau0780)
Supplement: Supplementary file 1 [file Science-363-eaau0780_S1.pdf]

www.sciencemag.org/content/363/6430/eaau0780/suppl/DC1

Supplementary Materials for

Binodal, wireless epidermal electronic systems with in-sensor analytics for neonatal intensive care

Ha Uk Chung, Bong Hoon Kim, Jong Yoon Lee, Jungyup Lee, Zhaoqian Xie, Erin M. Ibler, KunHyuck Lee, Anthony Banks, Ji Yoon Jeong, Jongwon Kim, Christopher Ogle, Dominic Grande, Yongjoon Yu, Hokyung Jang, Pourya Assem, Dennis Ryu, Jean Won Kwak, Myeong Namkoong, Jun Bin Park, Yechan Lee, Do Hoon Kim, Arin Ryu, Jaeseok Jeong, Kevin You, Bowen Ji, Zhuangjian Liu, Qingze Huo, Xue Feng, Yujun Deng, Yeshou Xu, Kyung-In Jang, Jeonghyun Kim, Yihui Zhang, Roozbeh Ghaffari, Casey M. Rand, Molly Schau, Aaron Hamvas, Debra E. Weese-Mayer, Yonggang Huang, Seung Min Lee, Chi Hwan Lee, Naresh R. Shanbhag, Amy S. Paller*, Shuai Xu*, John A. Rogers*

*Corresponding author. Email: apaller@northwestern.edu (A.S.P.); stevexu@northwestern.edu (S.X.); jrogers@northwestern.edu (J.A.R.)

Published 1 March 2019, *Science* **363**, eaau0780 (2019)
DOI: 10.1126/science.aau0780

This PDF file includes:

Materials and Methods
Figs. S1 to S48
Tables S1 and S2
Captions for tables S3 and S4
References

Other supplementary material for this manuscript includes:

Tables S3 and S4 (Excel format)

Materials and Methods

Fabrication of the Metal Coil & Interconnectors and the Microfluidic Channel

A double layered copper (Cu) foil (18/5 μm thick, Oak Mitsui MicroThin Series) provided the material for the near field communication (NFC) coil and interconnectors (fig. S3). After lamination of this Cu foil onto the polydimethylsiloxane (PDMS, Sylgard 184, Dow-Corning; 10:1 weight ratio) coated glass slide, with the 5 μm thick Cu side down and 18 μm side up, the Cu foil was peeled off by hands. The NFC coil and interconnectors were micro-patterned by photolithography (photoresist AZ P4620, AZ Electronic Materials; spin-casting at 3000 rpm for 30 sec, soft baking on a hot plate at 110 °C for 3 min, UV irradiance for 500 mJ/cm^2, and development for ~50 sec with developer AZ 400K/ deionized (DI) water solution of 1:3 volume ratio), and wet etching (CE-100 copper etchant, Transense; ~2 min with frequent rinsing by DI water). Photoresist was removed with acetone (CAS number: 67-64-1, Extra Pure, SLR, Fisher Chemical), isopropyl alcohol (IPA; CAS number: 67-63-0, Extra Pure, SLR, Fisher Chemical), and DI water rinse. After the native Cu oxide on the surface was eliminated by using oxide remover (Flux, Worthington), electrical circuit components were assembled with indium/silver soldering paste (~130 °C, 1 min). The whole area of the electrocardiogram (ECG) epidermal electronic system (EES) and photoplethysmogram (PPG) EES was encapsulated by a low modulus silicone elastomer after the second metal layer (e.g. bridge and red/infrared (IR) light emitting diode (LED) part), the back side insulated by a thin PDMS coating, was connected with the first metal interconnectors.

For the microfluidic channel, photolithography defined SU-8 mold (photoresist SU-8 2100, MicroChem; spin-casting at 3000 rpm for 30 sec, soft baking on a hot plate at 65 °C for 3 min and 95 °C for 20 min, UV irradiance for 380 mJ/cm^2, post exposure baking on a hot plate at 65 °C for

3 min and 95 °C for 10 min, development for ~20 min with SU-8 developer, IPA rinsed, and hard baking at 120 °C for 30 min). Spin-casting a fluoropolymer (1 µm, OSCoR 2312 photoresist solution, Orthogonal Inc., 3000 rpm, 50 °C for 1 min) formed a thin antiadhesive layer on the SU-8 mold. An additional spin casting on top of the mold and curing at room temperature for 24 hours yielded a bottom PDMS (Sylgard 184, Dow-Corning; 10:1 weight ratio) substrate. Delamination from the SU-8 mold allowed sample placement on a glass substrate with the feature side facing up. Next, a thin film of a fluoropolymer (1 µm, OSCoR 2312 photoresist solution, Orthogonal Inc.) was spincast on a PDMS substrate and then thermally annealed (50 °C for 1 min) to yield a low energy coating.

After exposure to oxygen plasma generated at low power (8.5 W) radiofrequency (RF) at 500 mTorr (Plasma Cleaner PDC-32G, Harrick Plasma) for 20 sec, the upper part (electronic layer encapsulated by PDMS) and the lower part (thin PDMS layer with microfluidic channel) was aligned and bonded. Finally, the bottom side was covered with a Silbione (Silbione® RT Gel 4717 A&B, Bluestar Silicones) layer and a syringe with a micro-needle injected a blended solution (82:18, volume ratio) of ionic liquid (1-ethyl- 3-methylimidazolium ethyl sulfate [EMIM][$EtSO_4$], ≥ 98.5 %, 07784 Sigma-Aldrich, MSDS (*41*)) and silica gel (high-purity grade, pore size 6 nm, 200-425 mesh particle size, Sigma-Aldrich) into the microfluidic channel.

Mechanical Simulations

The commercial software ABAQUS (ABAQUS Analysis User's Manual 2010, V6.10) was used to study mechanics of the devices and to optimize the design layouts. The objectives are to ensure that the interfacial normal and shear stresses on the skin are below the low somatosensory perception of the device on the human skin (*42*); and the strain in the copper layers is below the

elastic limit such that no plastic yielding occurs (*43*). The PDMS (elastic modulus 500 kPa and Poisson's ratio 0.5), Silbione (elastic modulus 3 kPa and Poisson's ratio 0.5) and ionic liquid were modeled by hexahedron elements (C3D8R) while the stiff copper (elastic modulus 119 GPa and Poisson's ratio 0.34) film was modeled by composite shell elements (S4R). The positions of the chips and the widths of serpentine interconnects were optimized to satisfy the competing requirements from mechanical and electromagnetical designs. For example, narrow interconnects improve the elastic stretchability, but lead to an undesired increase in the electrical resistance. An iterative optimization process was adopted to carefully balance these competing requirements and other mechanical and electromagnetical considerations. The positions of the chips were optimized to avoid entanglement of interconnects and contact between components. The minimum work of adhesive G_{min} required to prevent device delamination from the stretched skin was also shown in fig. S28. For stretching less than 20%, G_{min} is small such that the van der Waals force between the devices and skin is enough to provide this adhesion (16 N/m), i.e., without the need of adhesives.

Electromagnetic Simulations

Finite element method (FEA) was used in the electromagnetic simulations to determine the inductance, quality factor (Q factor) and the scattering parameters S11 of the ECG EES and PPS EES in undeformed and deformed states. The simulations were performed using the commercial software Ansys HFSS (Ansys HFSS 15, Ansys Inc. 2012), where the lumped port was used to obtain the scattering parameters S11 and port impendence Z. An adaptive mesh (tetrahedron elements) together with a spherical surface (1000 mm in radius) as the radiation boundary, was adopted to ensure computational accuracy. The inductance (L) and Q factor (Q) (fig. S2) were obtained from $L = \text{Im}\{Z\}/(2\pi f)$ and $Q = |\text{Im}\{Z\}/\text{Re}\{Z\}|$, where Re{Z}, Im{Z} and f represent the

real and imaginary part of the Z and the frequency, respectively. For 20% stretching of the loop antennas of the ECG EES and PPG EES, the changes of the inductance, Q factor and resonant frequency are less than 5% (figs. S10 and S11). For bending radii >~140 mm for the chest and >~50 mm for the foot, the inductance, Q factor and resonant frequencies are approximately unchanged (figs. S5 and S6).

Simulation for peel test

The commercial software ABAQUS (ABAQUS Analysis 2010, V6.10) was also used to study peel force of conventional adhesive (elastic modulus 5 MPa and Poisson's ratio 0.5), and EES adhesive with/without the ionic liquid layer (fig. S18), and with different patterns of holes (fig. S20). The adhesion energy of the interface between the phantom skin and EES/conventional adhesives obtained from experiments were 16 N/m and 175 N/m, respectively. The cohesive strengths σ_0 were estimated as 20 kPa and 50 kPa for the EES adhesive and conventional adhesive, respectively, from Fig. 2E. The peeling velocity was 0.53 mm/s, from experiments. The cohesive zone model is shown in fig. S16.

Simulations of electromagnetics associated with magnetic resonance imaging (MRI) imaging

The finite element method was used to determine the magnetic fields. The simulations were performed using a commercial software (Ansys HFSS 15, Ansys Inc. 2012), where adaptive mesh (tetrahedron elements) together with a spherical surface (2000 mm in radius) as the radiation boundary, was adopted to ensure computational accuracy. The in-plane gradient of the magnetic field density underneath the electrodes (Fig. 3, A and E) was obtained from $\left|\nabla_p B\right| = [(\partial B/\partial x)^2 + (\partial B/\partial y)^2]^{1/2}$ and $|\nabla_z B| = |\partial B/\partial z|$ with a working frequency of 128 MHz, where

B is the magnitude of the magnetic field density, and x, y, z, are the orthogonal coordinates of in the electrode plane.

Simulations for thermal load of EES sensors

The commercial software ABAQUS (ABAQUS Analysis 2010, V6.10) was used to study the temperature change of the skin for one-time MRI scan. The oscillating magnetic field density in MRI is B = 20 μT, and the working time for one-time scan of MRI is 0.5 ms. The received power of the ECG EES from the electromagnetic simulation is imported into ABAQUS for thermal analysis. The convective heat transfer coefficient of air is 6 W / ($m^2 \cdot K$) (*44*). Except for Cu, the hexahedron elements (DC3D8) were used, whereas thin Cu layer was modeled by the shell elements (DS4). The minimal mesh size was 1/10 of the thickness (10 μm) of the ionic layer, and the mesh convergence of the simulation was ensured. The thermal conductivity, heat capacity and mass density used in the simulations are 0.35 $W \cdot m^{-1} \cdot K^{-1}$, 2135 $J \cdot kg^{-1} \cdot K^{-1}$ and 1490 $kg \cdot m^{-3}$ for the skin (*43*); 0.15 $W \cdot m^{-1} \cdot K^{-1}$, 1510 $J \cdot kg^{-1} \cdot K^{-1}$ and 1000 $kg \cdot m^{-3}$ for PDMS (*45*); 0.15 $W \cdot m^{-1} \cdot K^{-1}$, 2200 $J \cdot kg^{-1} \cdot K^{-1}$ and 1100 $kg \cdot m^{-3}$ for ionic liquid (*44*); 0.15 $W \cdot m^{-1} \cdot K^{-1}$, 1460 $J \cdot kg^{-1} \cdot K^{-1}$ and 970 $kg \cdot m^{-3}$ for Silbione (*42*); and 386 $W \cdot m^{-1} \cdot K^{-1}$, 383 $J \cdot kg^{-1} \cdot K^{-1}$ and 8954 $kg \cdot m^{-3}$ for Cu (*46*), respectively.

Simulations for electromagnetics associated with the specific absorbed radiation (SAR) and maximum permissible exposure (MPE)

The finite element method (Ansys HFSS 15, Ansys Inc. 2012) is used to determine whether the NICU platform is operating within the specific absorbed radiation (SAR) and maximum permissible exposure (MPE) requirements outlined by the Federal Communications Commission (FCC) CFR Part 1.1310. The neonates with 24- and 40-week gestational ages are modeled as the

ellipsoids with major (half) axes 30, 50 and 115 mm, and 55, 90 and 210 mm, respectively, which are estimated according to the size of the neonate with different gestational ages (*47*). The impendence of the circuit part is assumed to be 50 Ω, in series with the antenna. The distance between the neonate and the antennas of ECG EES and PPG EES is 250 μm, which is the distance when the ECG EES/PPG EES is mounted on the neonate. The density of the neonate is 1000 kg/m^3. The maximum values of SAR for the ECG EES mounted on the neonates with 24- and 40-week gestational ages are 0.005 W/kg (fig. S43) and 0.002 W/kg (fig. S44), respectively. The maximum values of SAR for the PPG EES mounted on the neonates with 24- and 40-week gestational ages are both 0.001 W/kg (fig. S45 and S46). These values are significantly less than the SAR limit for general population/uncontrolled exposure of 0.08 W/kg as averaged over the whole body (*48*). Additionally, the maximum computed equivalent power density of electromagnetic fields for ECG EES and PPG EES are 0.2 mW/cm^2 (fig. S47) and 0.07 mW/cm^2 (fig. S48), respectively, which are below the MPE limit of ~4.9 mW/cm^2 (*48*).

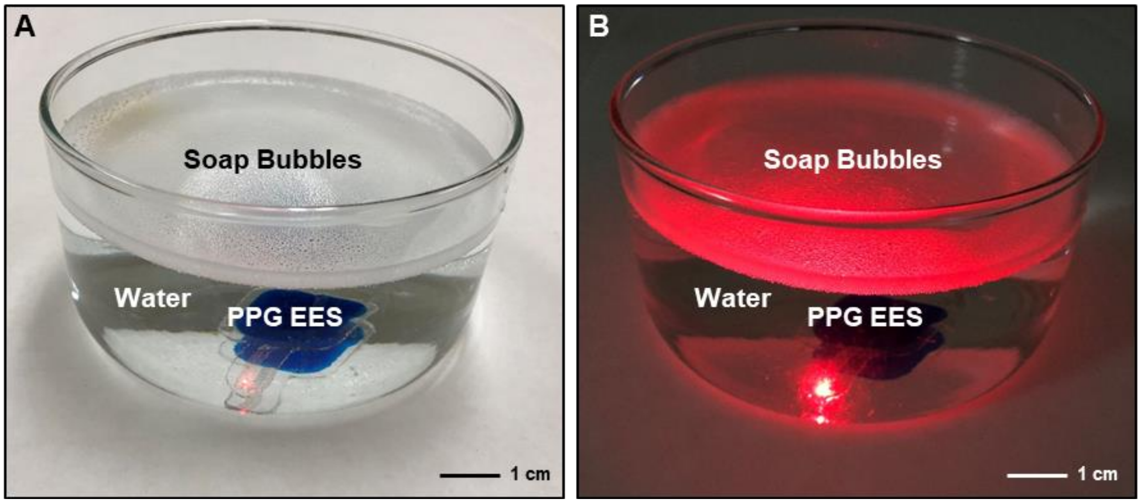

Fig. S1. Demonstration of operation of a PPG EES while immersed in soapy water. A PPG EES was immersed in soapy water **(A)** with and **(B)** without external lighting. The soapy water is to show the compatibility of the EES in highly humid environment.

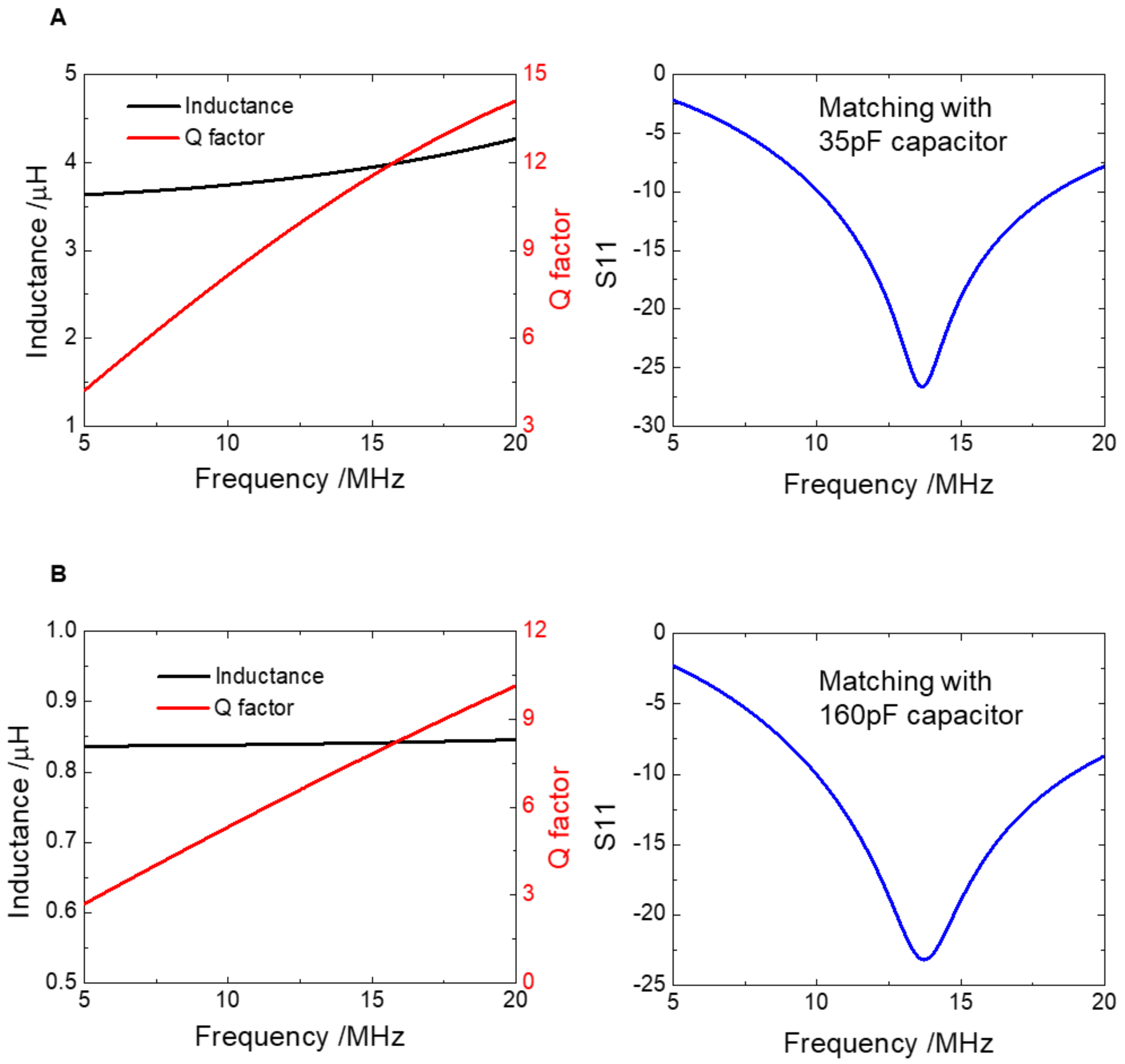

Fig. S2. Electromagnetic characteristics of the EES sensors. Inductance, Q factors, and S11 of **(A)** ECG EES and **(B)** PPG EES. The plots of S11 show matching condition to have resonant frequency at 13.56 MHz.

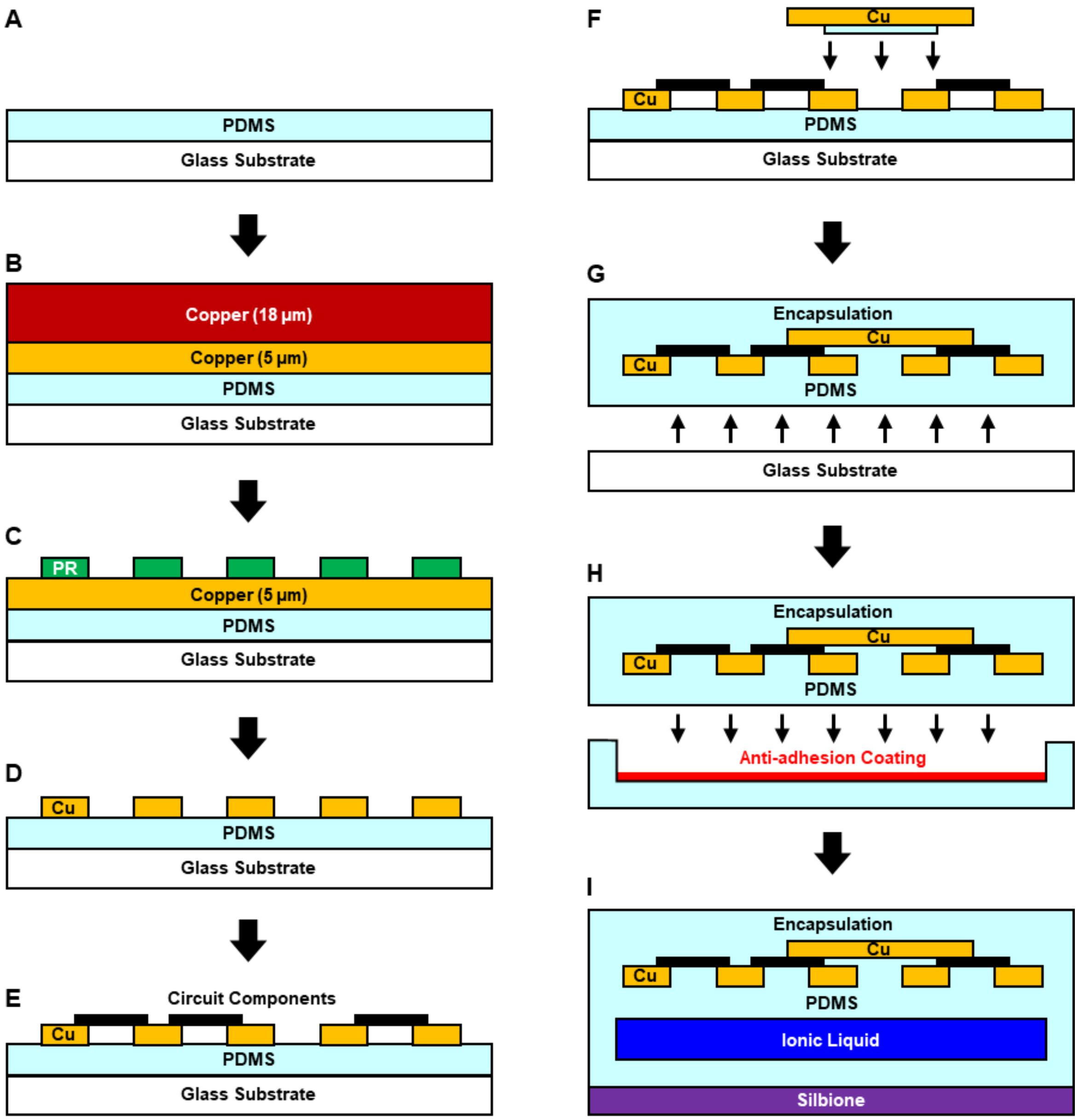

Fig. S3. Schematic illustration of the process for fabricating the ECG EES and PPG EES. (A) PDMS-coated glass slide substrate **(B)** laminated with a double layered copper foil, 18/5 µm thick, with the 5 µm thick side facing down. **(C)** After removal of the 18 µm thick Cu side, photoresist was spin-cast to **(D)** lithographically pattern the NFC coil and interconnects in the Cu layer. **(E)** Electrical circuit components were assembled onto the Cu circuit. **(F)** A second metal layer for the bridge and red/IR LEDs were connected to the first metal interconnects with the back side insulated by a thin PDMS coating. **(G)** The entire area of the ECG EES and PPG EES was encapsulated by PDMS layer, detached from the glass slide and then **(H)** attached to the bottom PDMS substrate with a spin-coated anti-adhesive layer inside the microfluidic channel. **(I)** The bottom side was covered with a Silbione layer. A syringe with a micro-needle injected a blended solution of ionic liquid and silica gel into the microfluidic space.

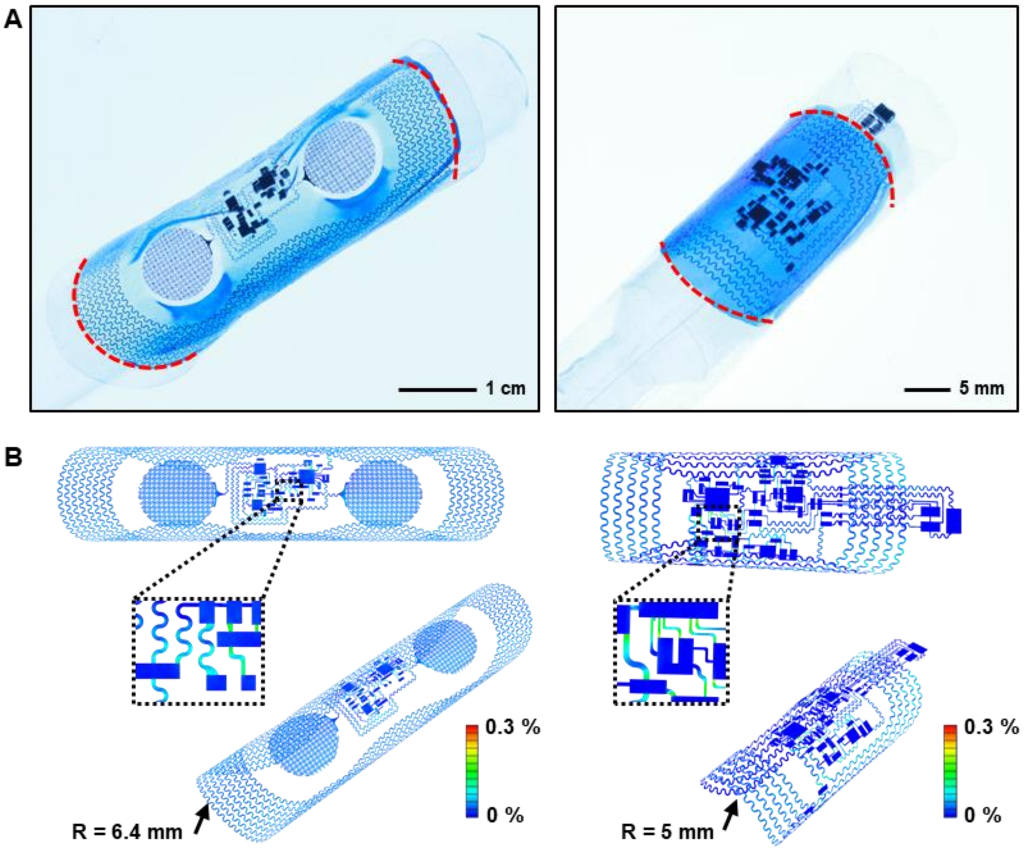

Fig. S4. Computational analysis of mechanics associated with bending the ECG EES and PPG EES. (A) Photographic images of ECG EES and PPG EES bent around a plastic tube. **(B)** Finite element analysis results of the strain in the copper layer of the ECG EES and PPG EES on microfluid channel bending to a radius of ~6.4 mm and ~5 mm, respectively.

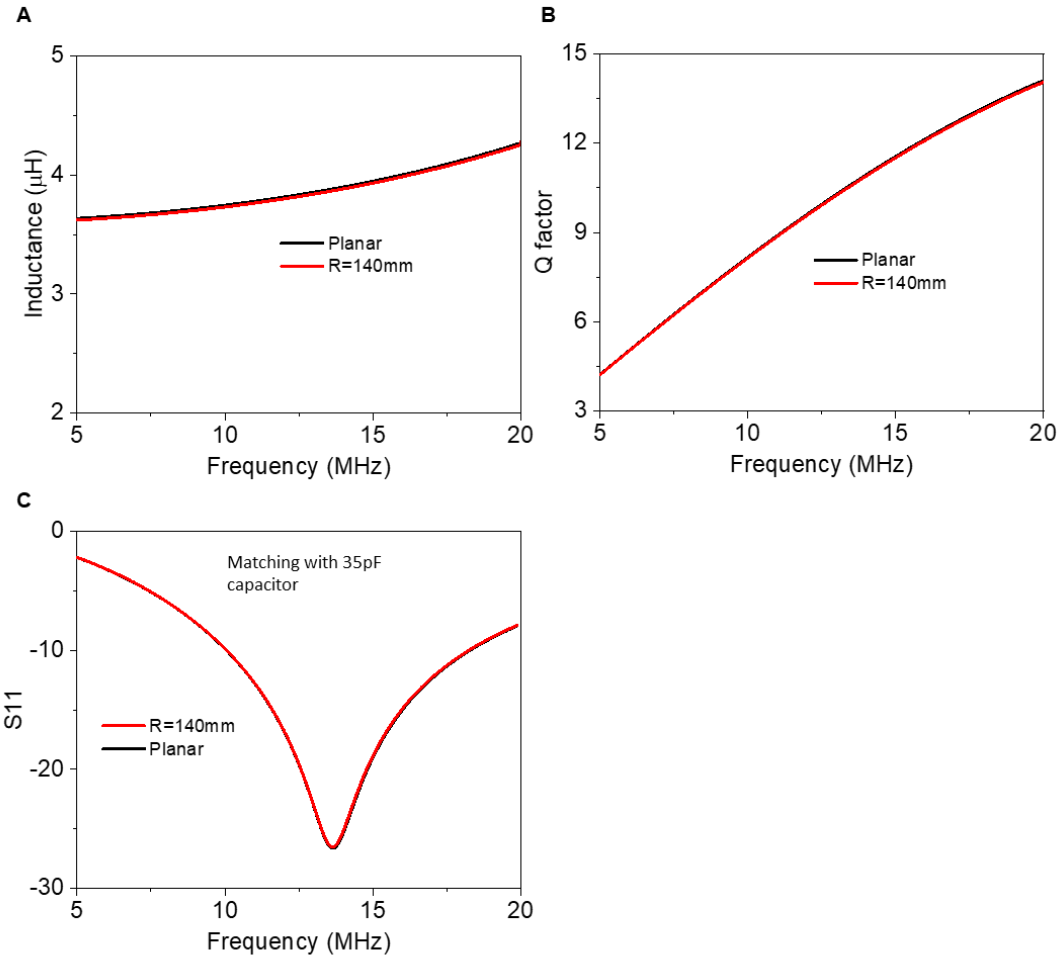

Fig. S5. Electromagnetic characteristics of an ECG EES while bent. Changes of **(A)** inductance, **(B)** Q factor, and **(C)** S11 of the ECG EES when bent. Plots are showing negligible changes in electromagnetic properties of the ECG EES when bent compared to planar case.

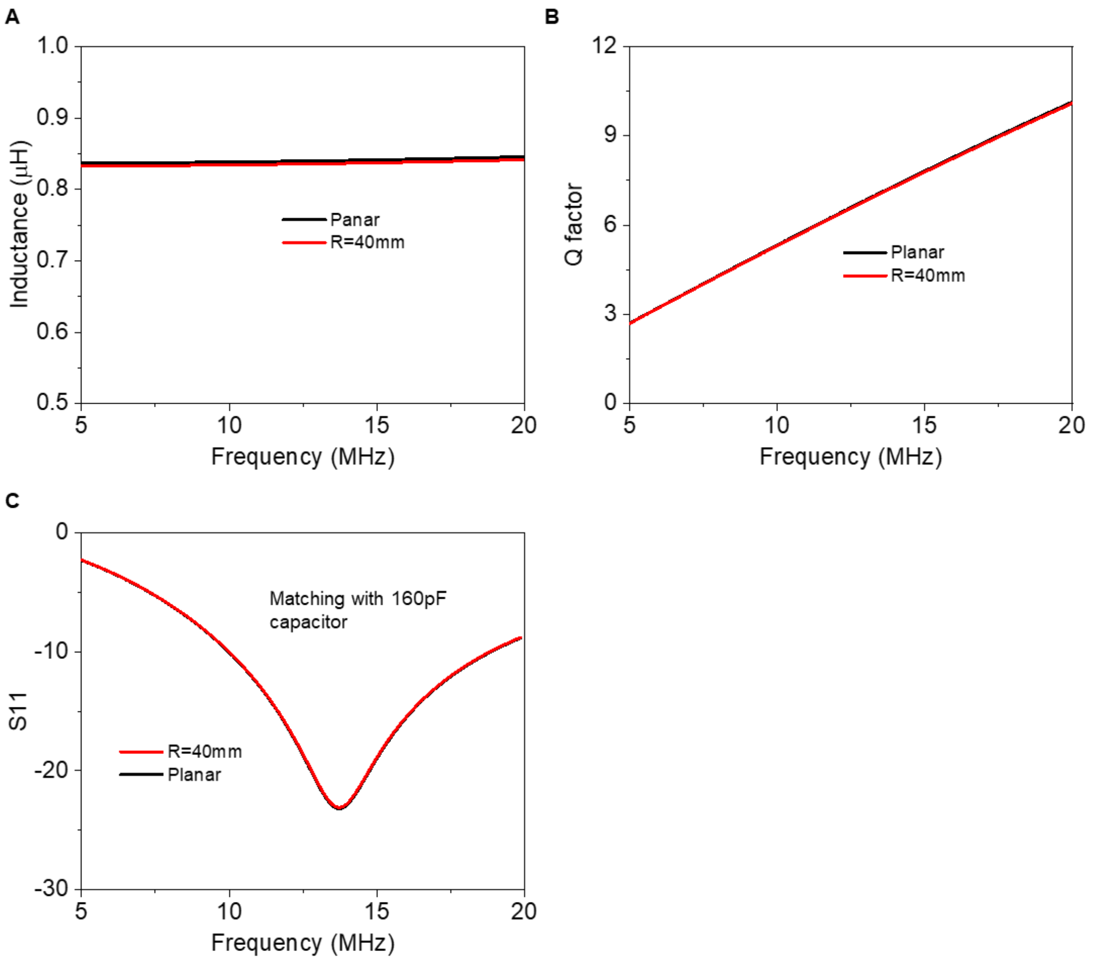

Fig. S6. Electromagnetic characteristics of a PPG EES while bent. Changes of **(A)** inductance, **(B)** Q factor, and **(C)** S11 of the PPG EES when bent. Plots are showing negligible changes in electromagnetic properties of the PPG EES when bent compared to planar case.

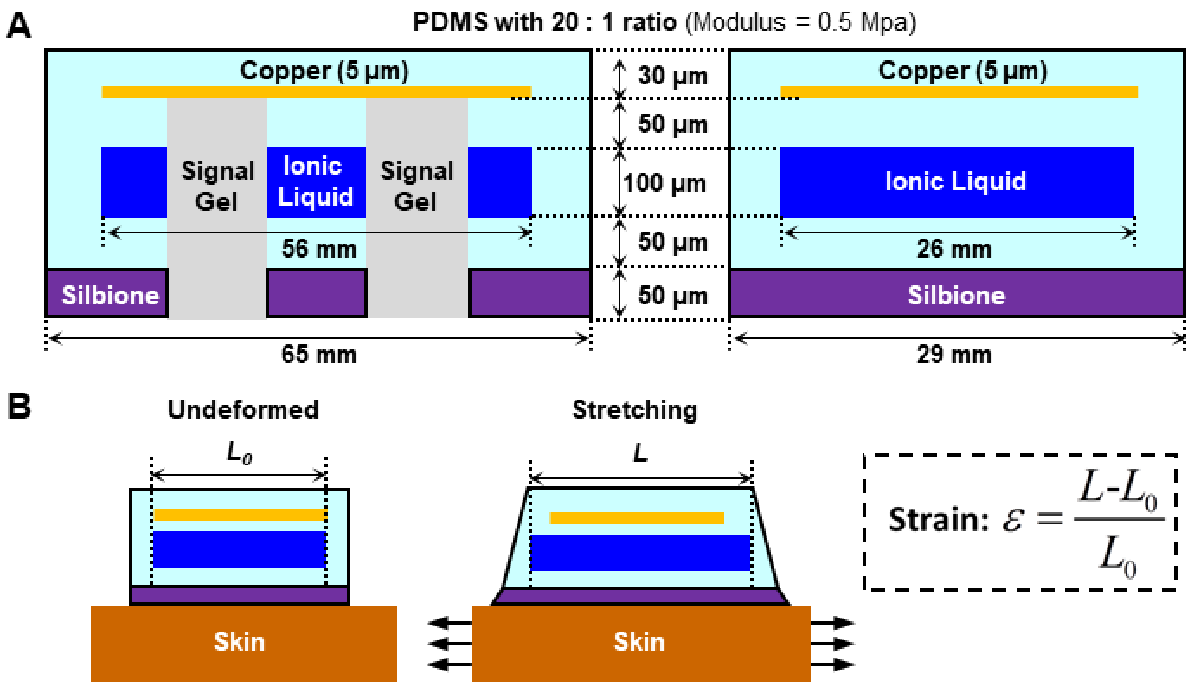

Fig. S7. Mechanics of ECG EES and PPG EES with embedded microfluidic channel. A schematic illustration of **(A)** the cross-section of the ECG EES and PPG EES and **(B)** the undeformed and uniaxially-stretched ECG EES and PPG EES with an underlying microfluid channel.

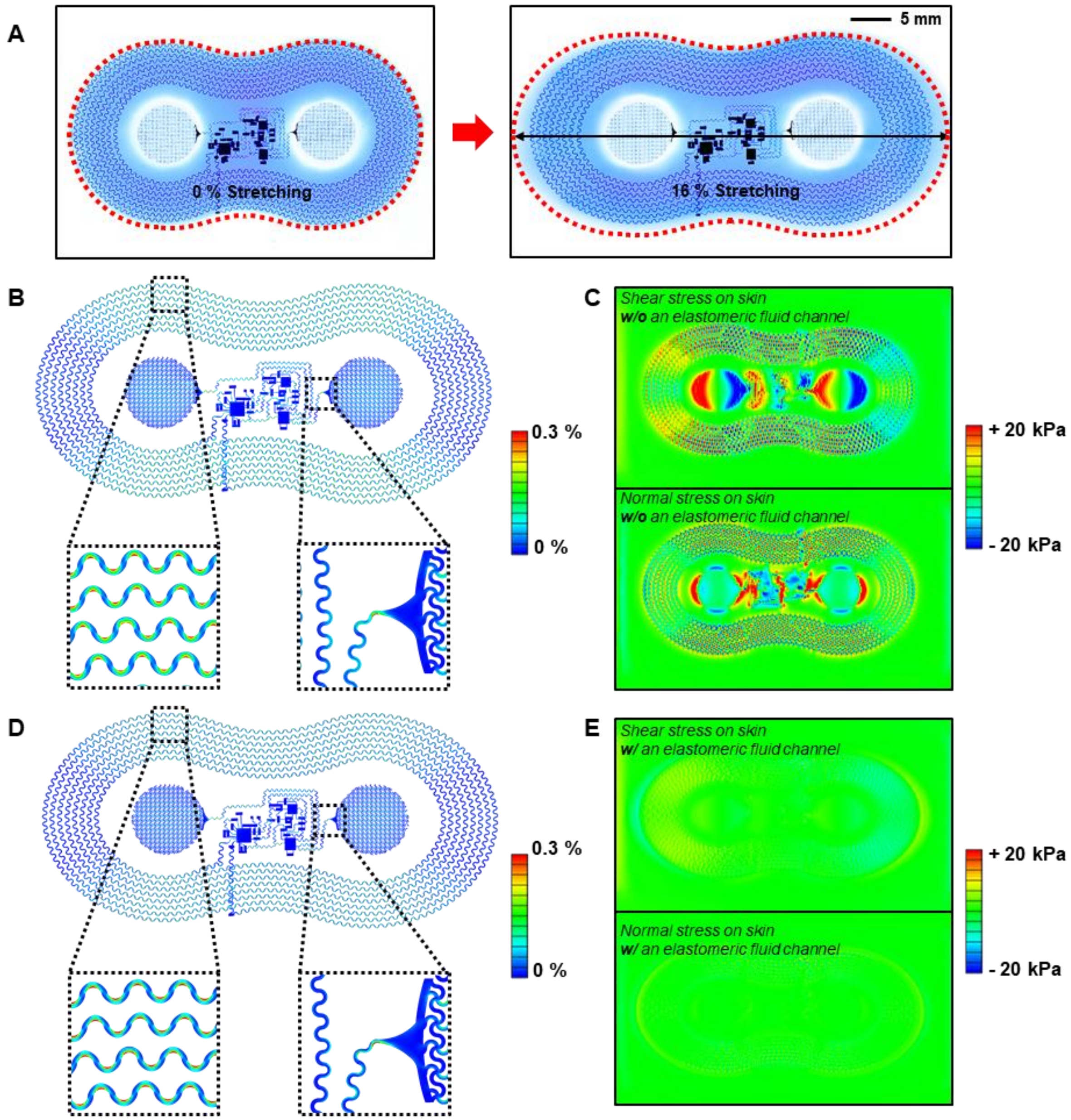

Fig. S8. Mechanics of stretching an ECG EES in a uniaxial fashion. (A) Photographic images of an undeformed and a uniaxially-stretched (~16 % defined as in fig. S7) ECG EES on microfluid channel. **(B)** The strain in the copper layer for an ECG EES without microfluid channel during 8% stretching deformation, and **(C)** the corresponding shear/normal stresses on the skin. **(D)** The strain in the copper layer for an ECG EES with microfluid channel during 16 % stretching deformation, and **(E)** the corresponding shear/normal stresses on the skin. For ~16 % stretching of ECG EES on an microfluid channel, both the shear and normal stresses on the skin are less than ~20 kPa, the threshold of skin sensation.

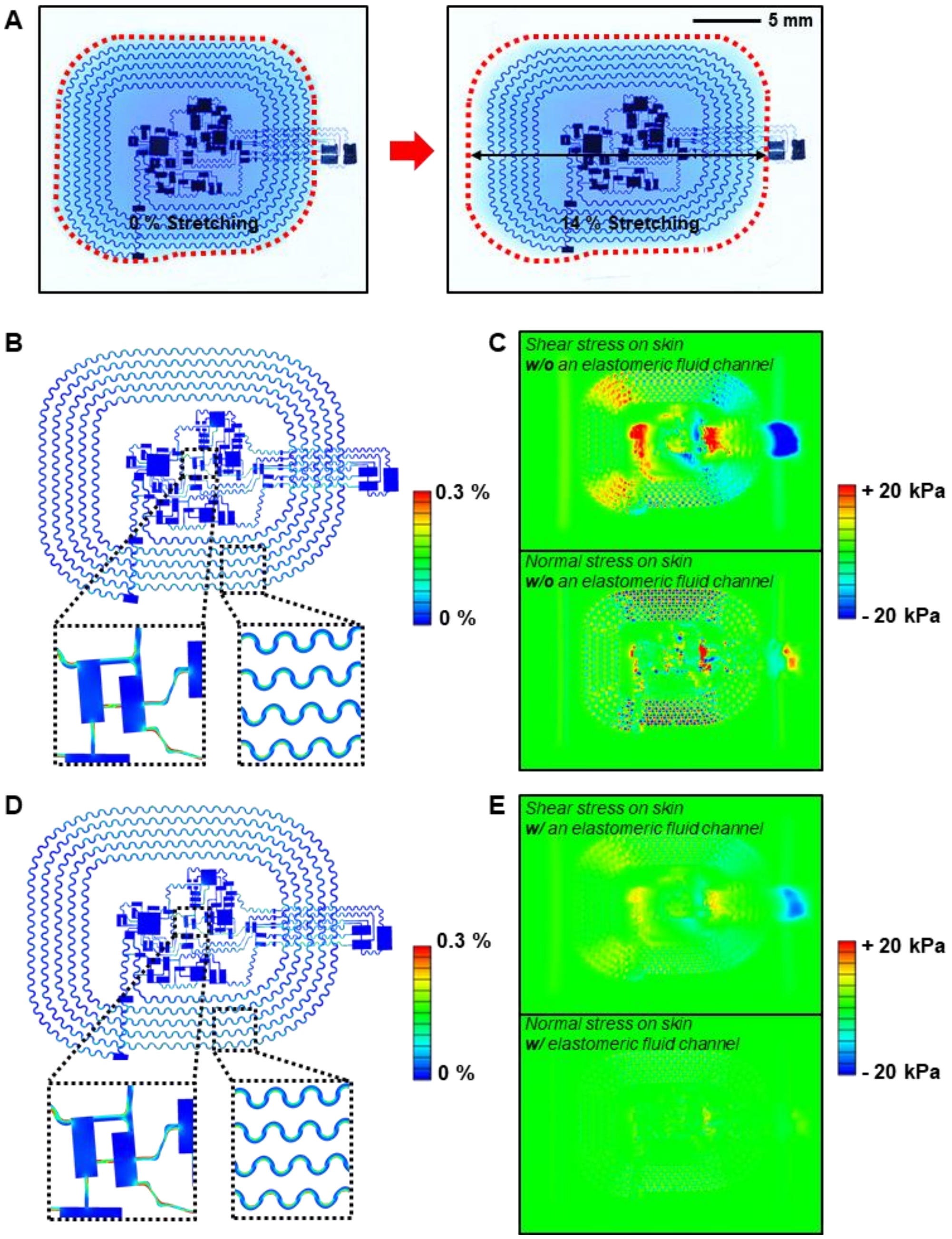

Fig. S9. Mechanics of stretching a PPG EES in a uniaxial fashion. (A) Photographic images of an undeformed and a uniaxially-stretched (~13 % defined as in fig. S7) PPG EES on microfluid channel. **(B)** The strain in the copper layer for an PPG EES without microfluid channel during 7% stretching deformation, and **(C)** the corresponding shear/normal stresses on the skin. (**D**) The strain in the copper layer for an PPG EES with microfluid channel during 13% stretching deformation, and **(E)** the corresponding shear/normal stresses on the skin. For ~13 % stretching of PPG EES on an microfluid channel, both the shear and normal stresses on the skin are less than ~20 kPa, the threshold of skin sensation.

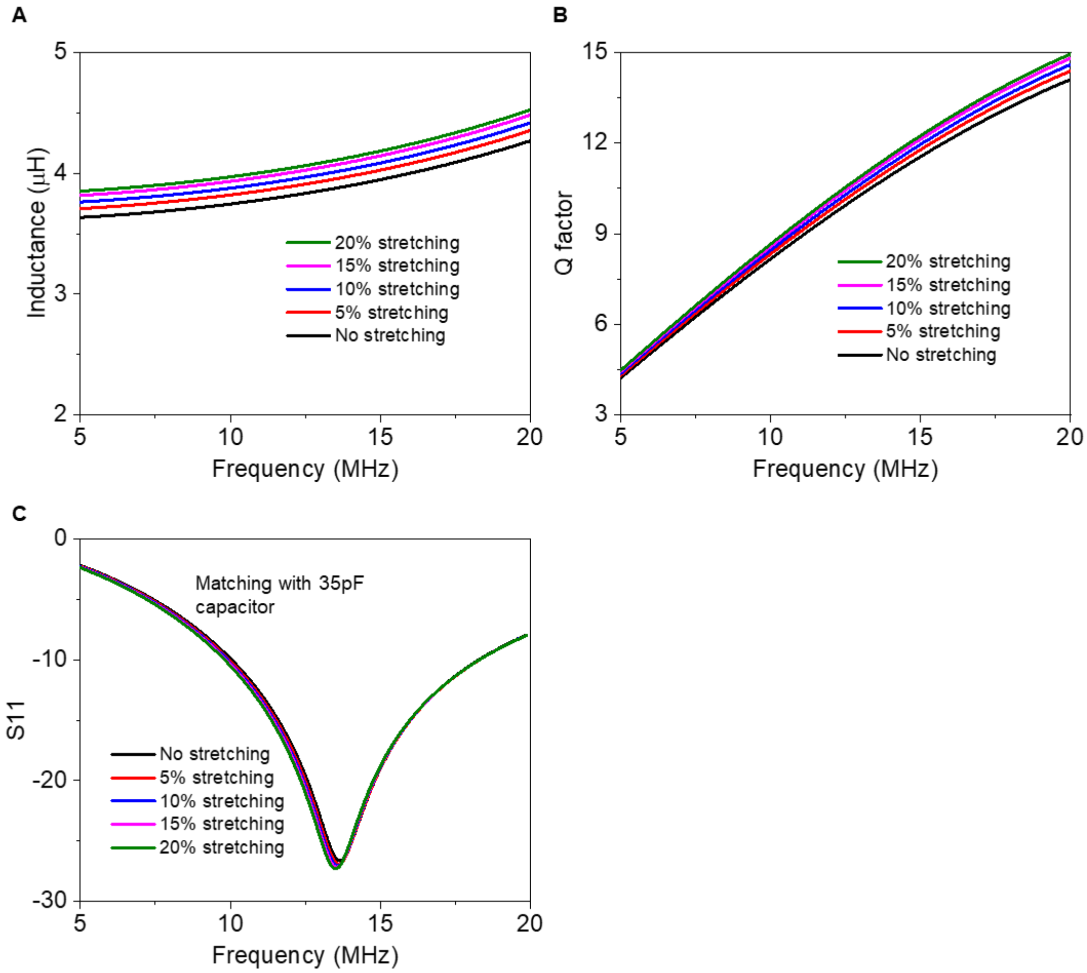

Fig. S10. Electromagnetic characteristics of an ECG EES while stretched. Changes of **(A)** inductance, **(B)** Q factor, and **(C)** S11 of the ECG EES when stretched. Plots are showing changes in electromagnetic properties of the ECG EES when stretched by the percentage labeled with different colors inside each plot.

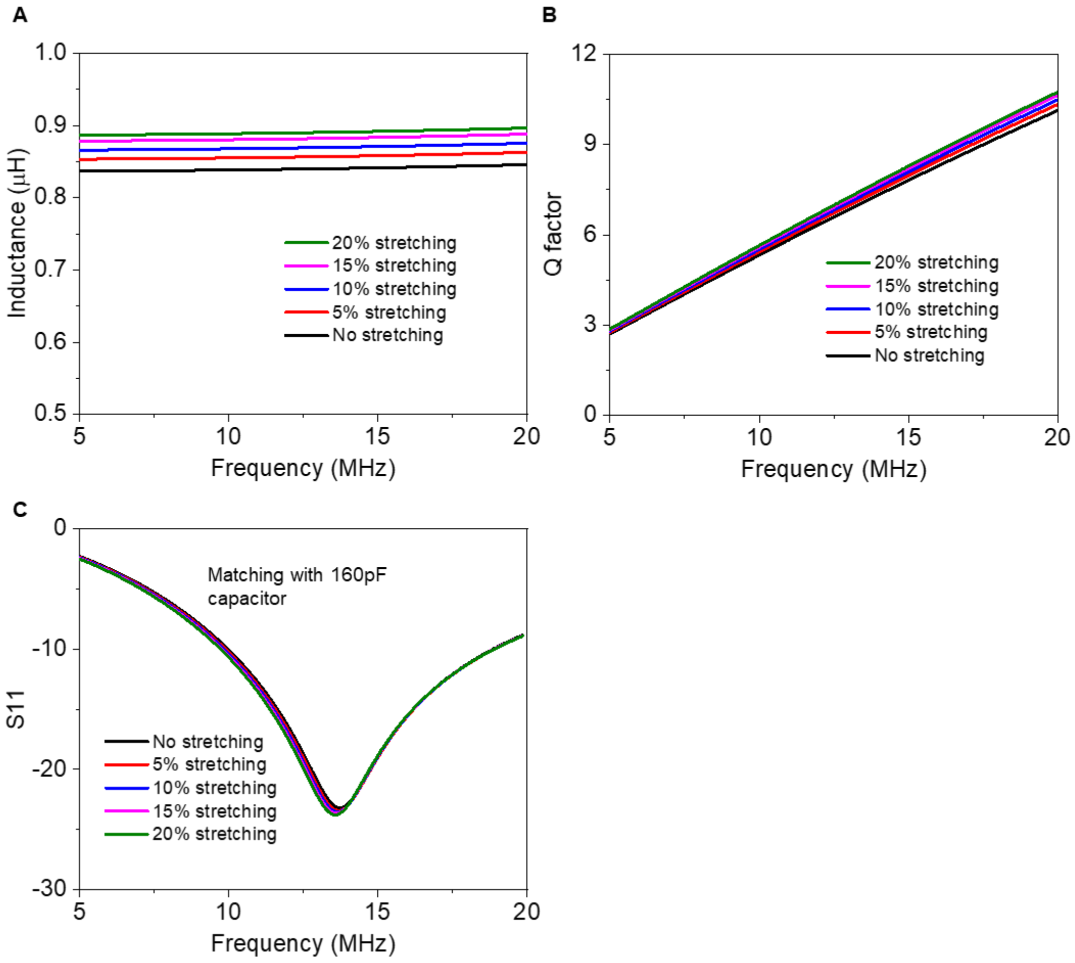

Fig. S11. Electromagnetic characteristics of a PPG EES while stretched. Changes of **(A)** inductance, **(B)** Q factor, and **(C)** S11 of the PPG EES when stretched. Plots are showing changes in electromagnetic properties of the PPG EES when stretched by the percentage labeled with different colors inside each plot.

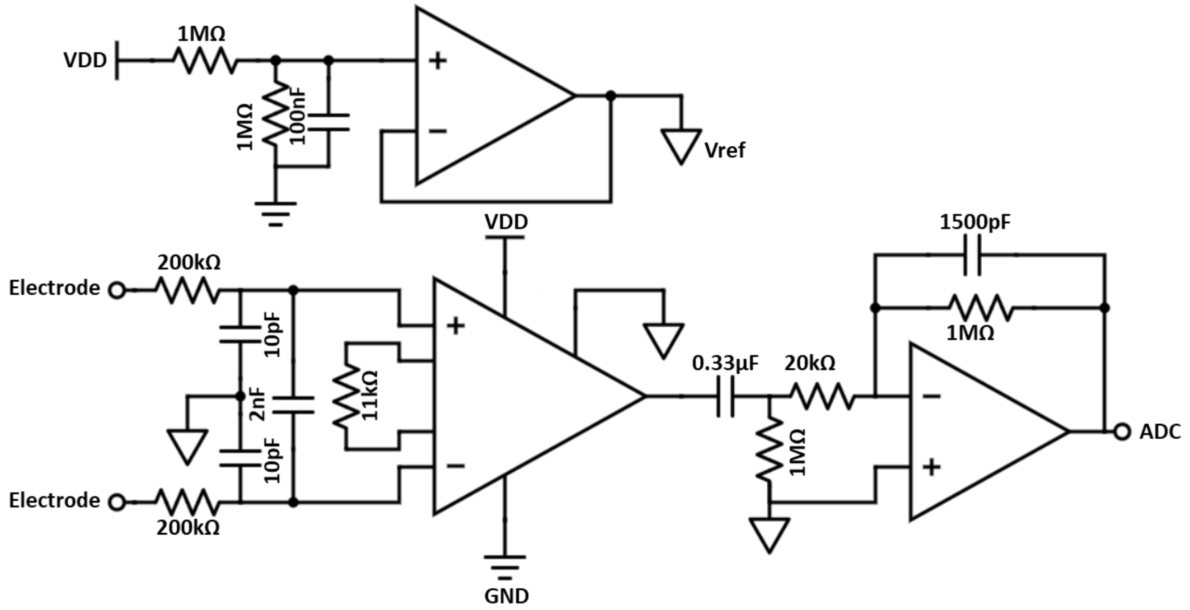

Fig. S12. Circuit diagram of an ECG EES. The ECG EES includes radio frequency interference (RFI) filter, an instrumentation amplifier, a band pass filter, and amplifier. The reference voltage (Vref) is the half of supply voltage (VDD) for voltage offset. The raw ECG signal passes through an RFI filter (f_c = 200 Hz) to suppress electromagnetic interference from the primary RF source. Subsequent amplification occurs via the instrumentation amplifier with a common mode-rejection ratio of 100 dB and input impedance of 100 GΩ (gain = 10 V/V). A passive resistor-capacitor (RC) high-pass filter (f_c = 0.5 Hz) eliminates the direct current (DC) offset. An inverting amplifier with a low-pass filter further amplifies the signal (gain = 50 V/V) and prevents aliasing (f_c = 100 Hz).

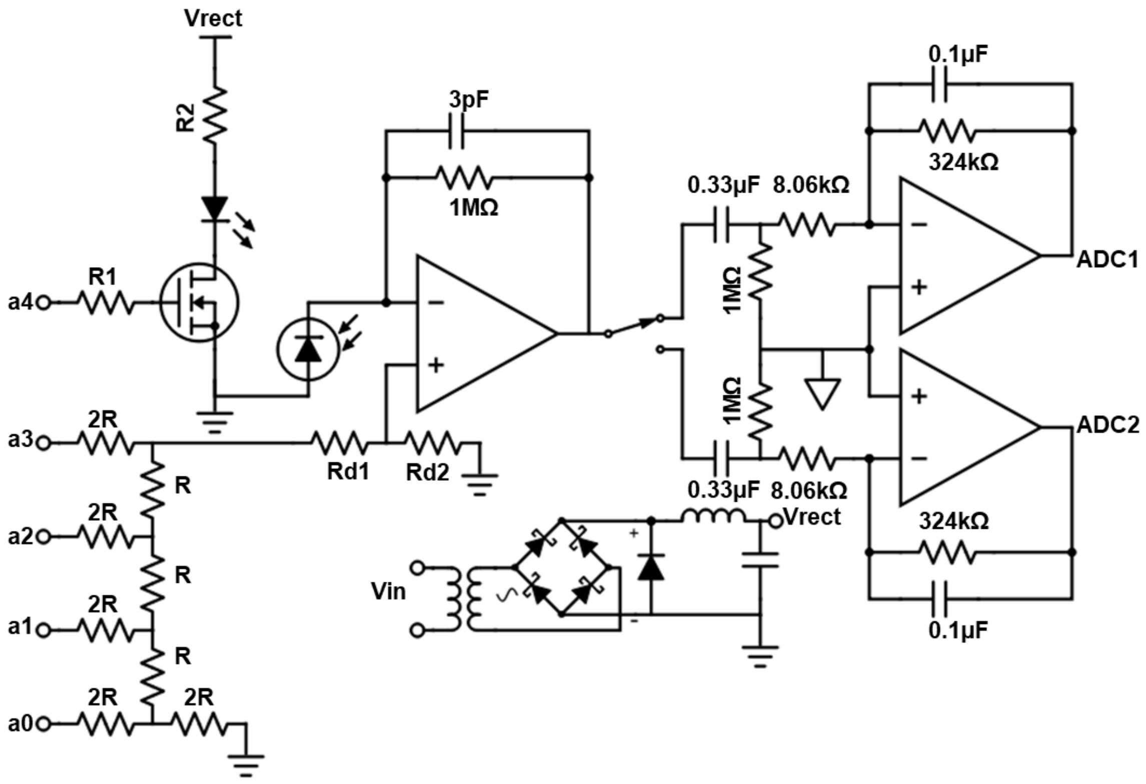

Fig. S13. Circuit diagram of a PPG EES. The PPG EES includes LED drivers, transimpedance amplifier, voltage rectifier with a buck converter, and amplifier including high and low pass filter. Each LED operates in a pulsed mode with 50 % duty cycle, out of phase with one another, via current supplied through a LED driver. The power supply originates from a separate full-wave rectifier and a buck converter, coupled to a single RF harvesting antenna to bypass limitations associated with the internal rectifier on the NFC. The photodiode captures backscattered light associated with operation of each LED. The output passes through a transimpedance amplifier (transimpedance gain = 1 V/µA) followed by a passive high-pass filter (f_c = 0.5 Hz) to eliminate DC offset and an inverting amplifier (gain = 40 V/V) with a low-pass filter configuration (f_c = 5 Hz) to avoid aliasing. The baseline can be controlled by general purpose input/output automatically based on the output signal.

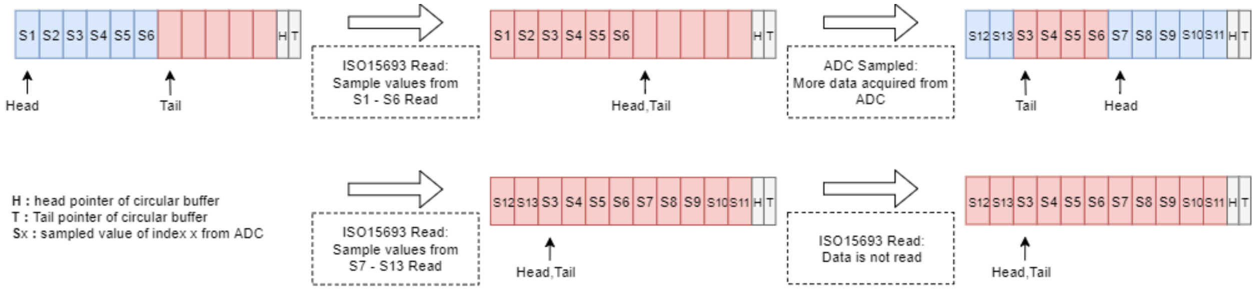

Fig. S14. Flow chart of the circular buffer for incoming data. Data communication in ISO15693 standards between transponder and reader with a circular buffer designed to properly index samples.

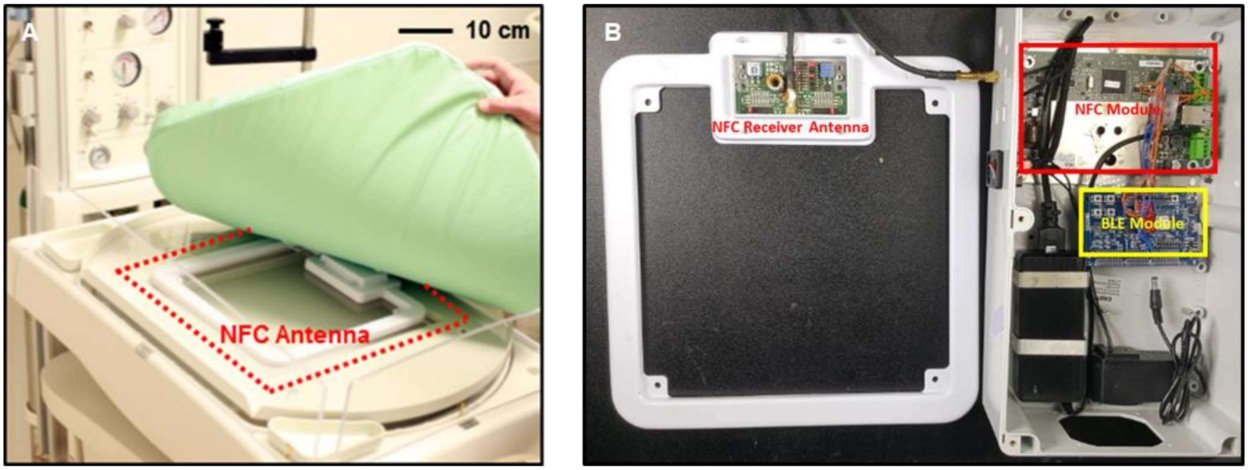

Fig. S15. Images of the measurement setup and equipment. (A) Measurement setup in a neonatal intensive care unit (NICU) setting with an NFC antenna placed unobstructively underneath the mattress. **(B)** Picture of the NFC/ Bluetooth Low Energy (BLE) reader system, which consists of an NFC receiver antenna, NFC reader module, and BLE module.

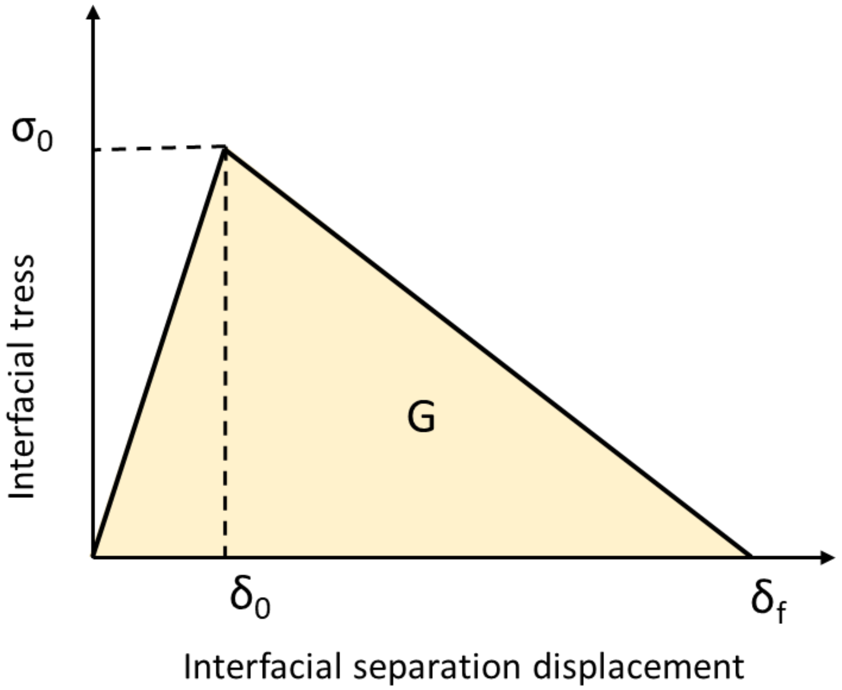

Fig. S16. Cohesive-zone modeling of the EES adhesive/skin interface. The cohesive strength σ_0 represents the initial delamination, δ_f represents the complete delamination, and $G = \sigma_0 \delta_f /2$ is the adhesion energy.

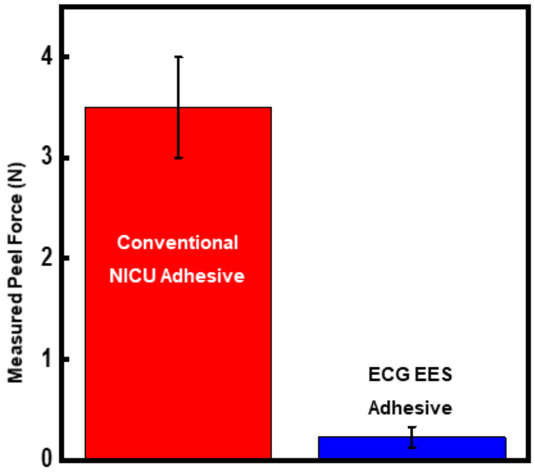

Fig. S17. Comparison of peel force from a conventional NICU adhesive and an ECG EES adhesive. The sample size was ~2 cm x 4 cm. After cleaning the surface of the skin with an alcohol pad and allowing it to dry in open air for one minute, we applied the adhesives and allowed them to remain in place for 3 minutes before performing peel tests.

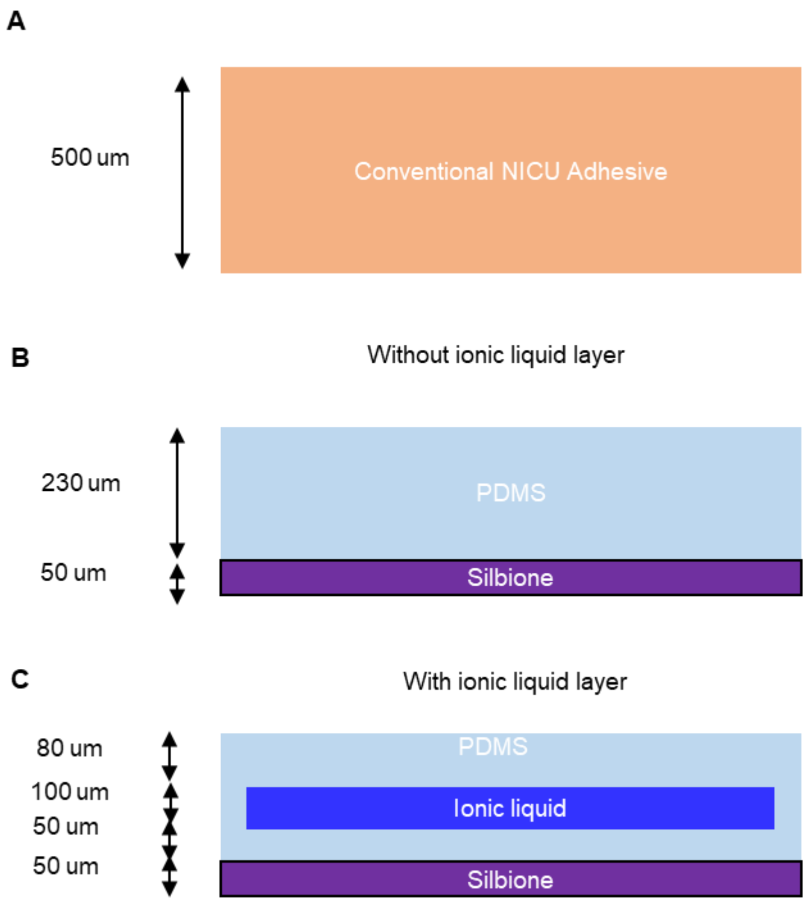

Fig. S18. Dimensions of adhesives assumed in computational studies of peeling force. (A) Cross-sectional view of the conventional NICU adhesive, and the EES adhesive with **(B)** and without **(C)** the ionic liquid layer, i.e. the microfluidic channel. Their in-plane sizes are all 4 cm x 2 cm.

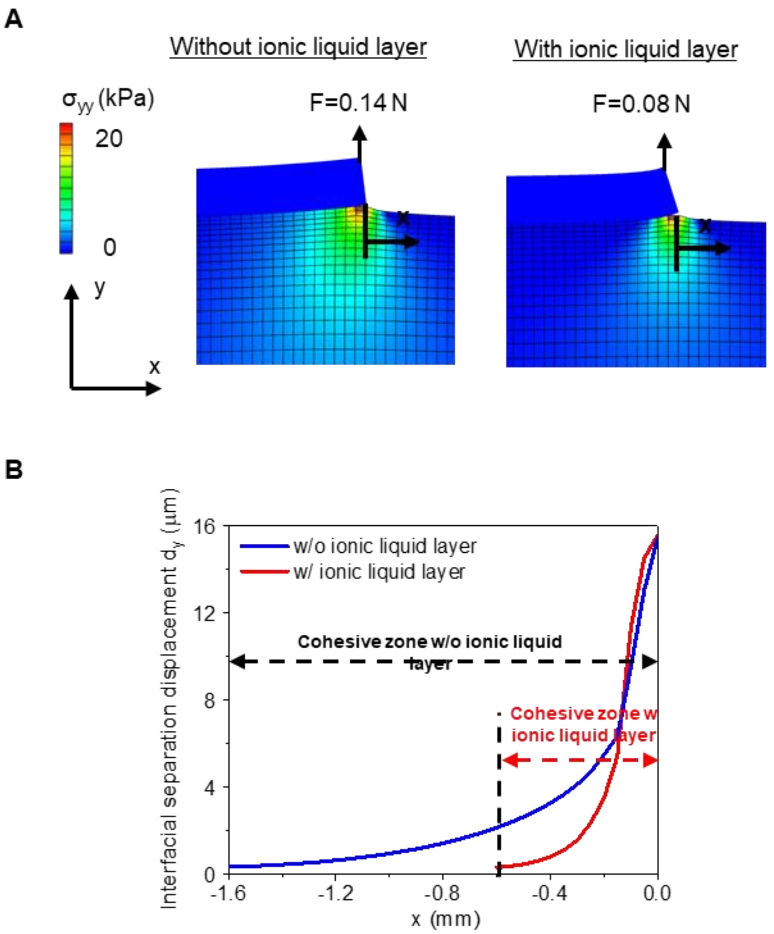

Fig. S19. Computational results for delamination of adhesives from the skin. (A) FEA results, and associated mesh, for the deformation of the skin and the EES adhesive with and without the ionic liquid layer (microfluidic channel) at the instant of initial delamination. The contour plots show the distribution of normal stress (σ_{yy}). **(B)** The corresponding separation of the interface between the skin and EES adhesive.

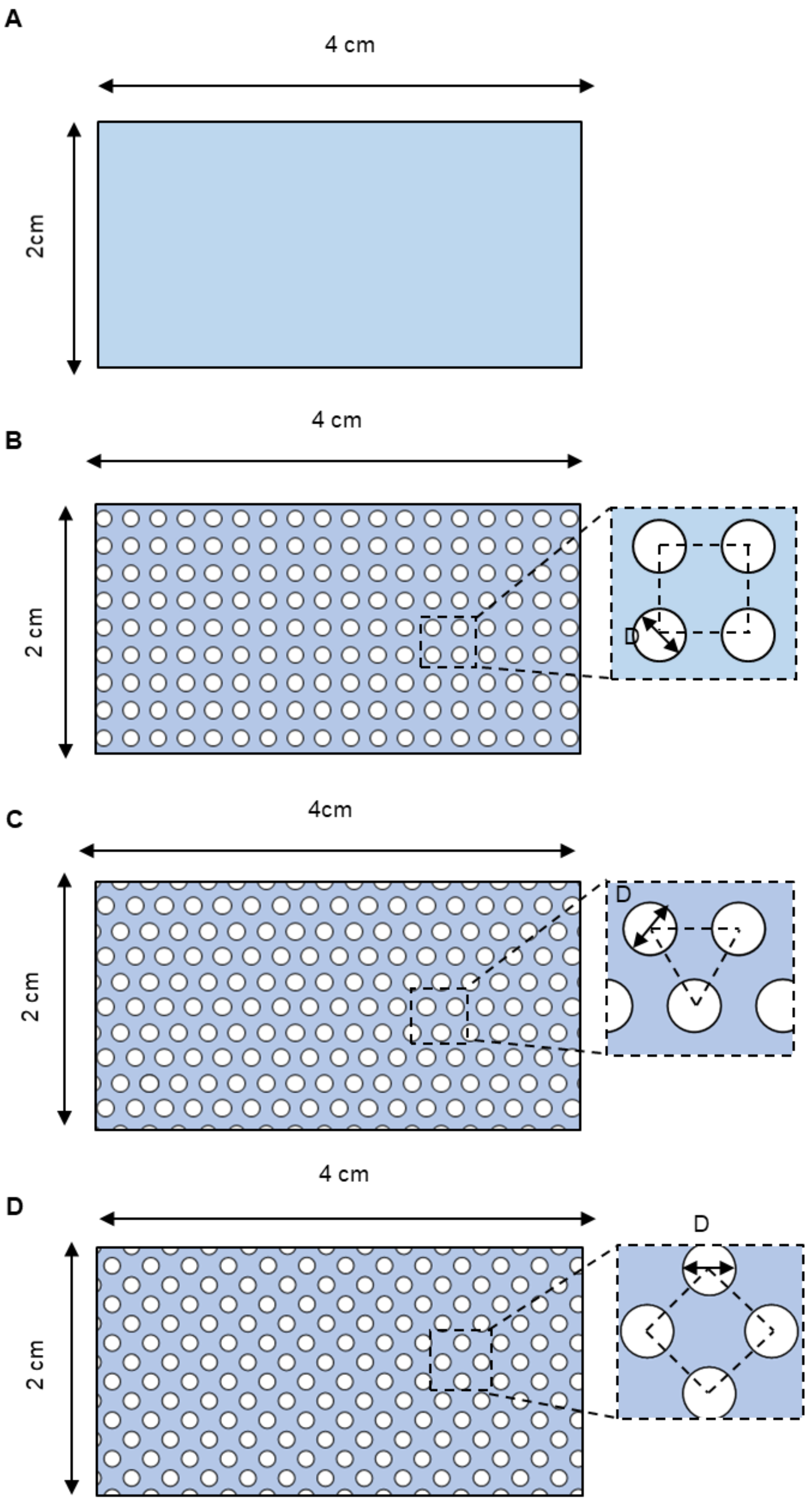

Fig. S20. Diagrams of adhesives in four different perforation patterns. (A) The EES adhesive without holes. The EES adhesive with holes of diameter *D;* **(B)** regular square pattern; **(C)** regular triangular pattern; and **(D)** square pattern with 45 ^{o} rotation.

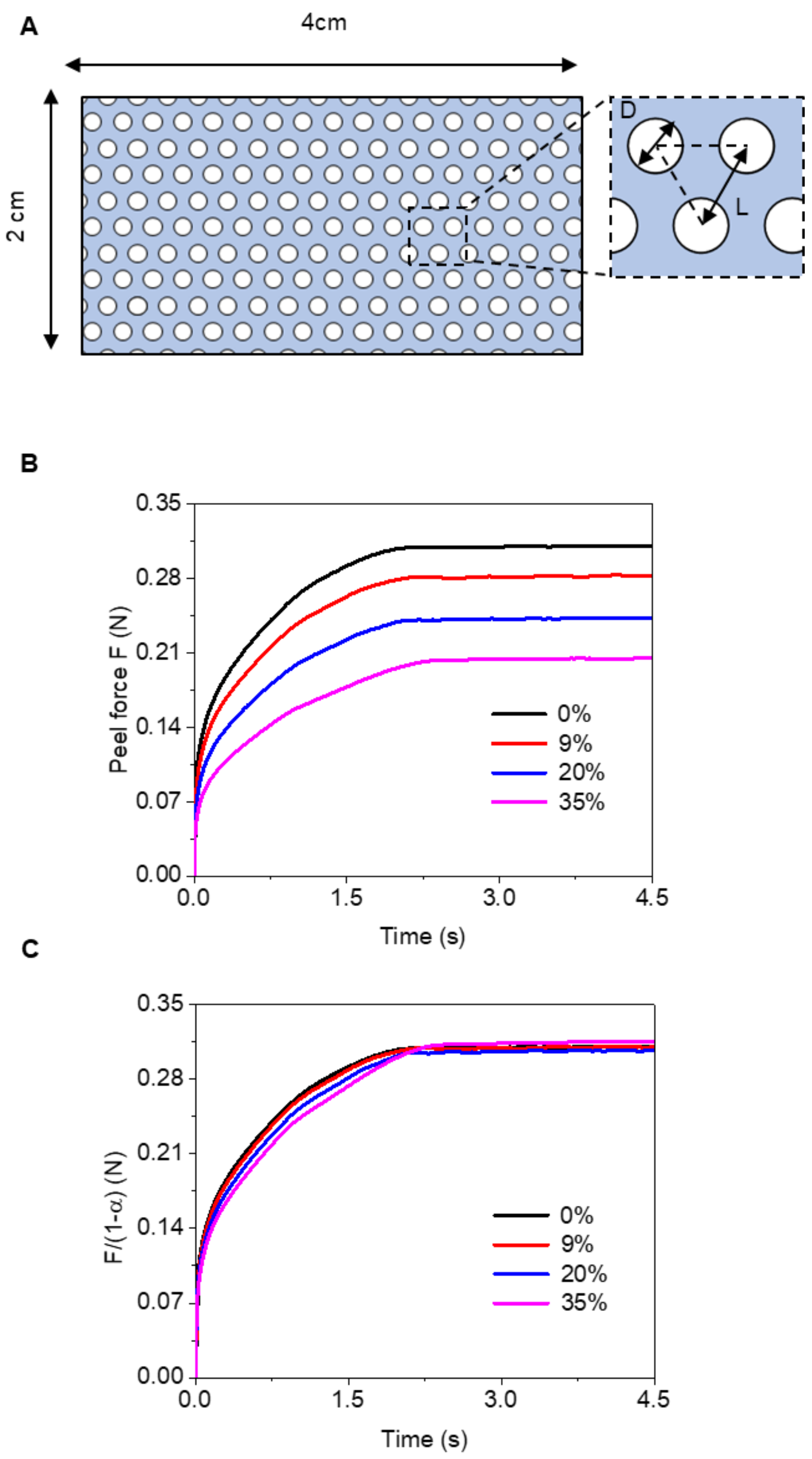

Fig. S21. Effect of a triangular perforation pattern in the EES adhesive. (A) The EES adhesive with regular triangular holes of small diameter D = 200 µm. **(B)** The peel force versus time for different hole area fractions α. **(C)** The normalized peel force F/(1- α) versus time.

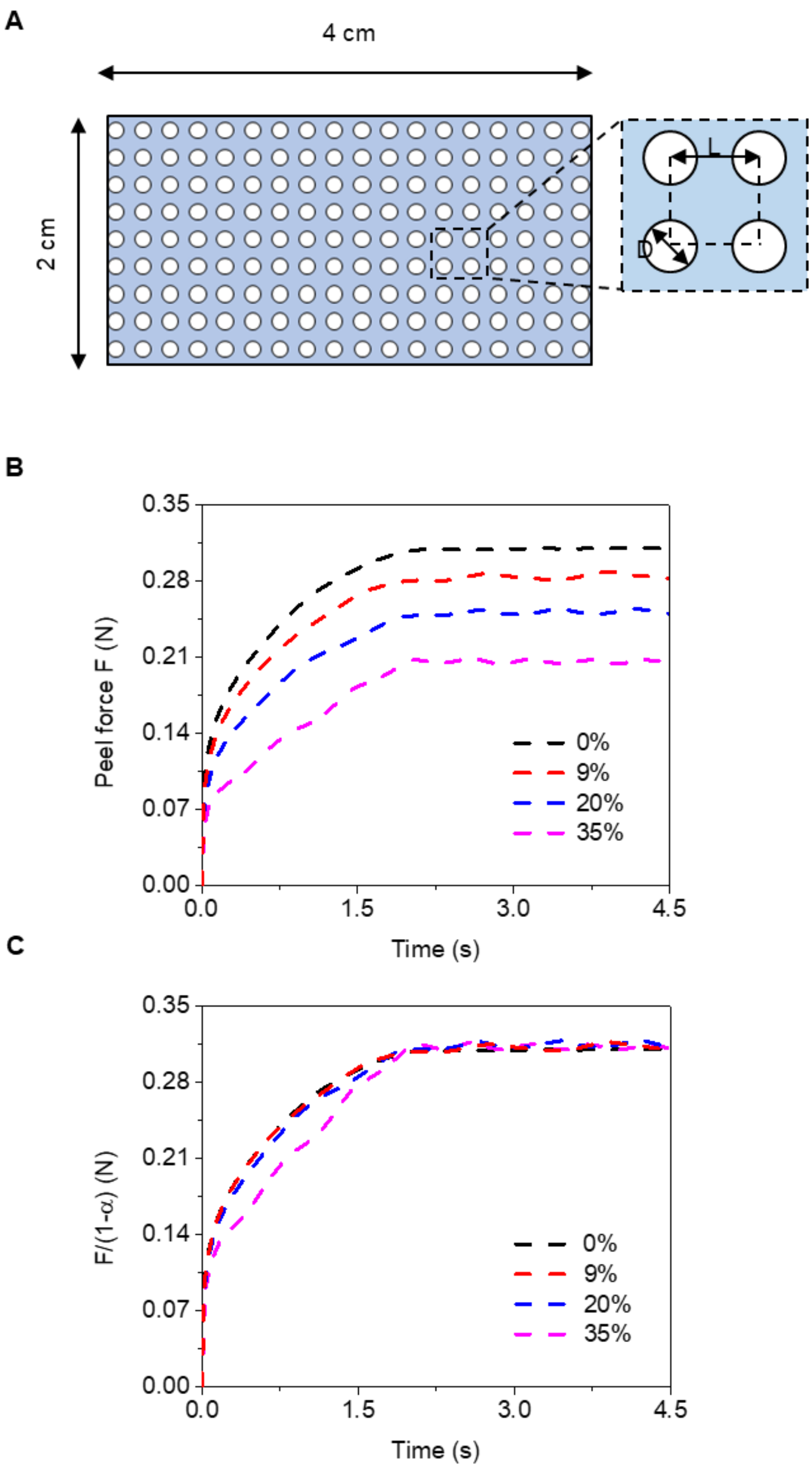

Fig. S22. Effect of a regular square perforation pattern in the EES adhesive. (A) The EES adhesive with square holes of small diameter D = 200 μm. **(B)** The peel force versus time for different hole area fractions α. **(C)** The normalized peel force F/(1- α) versus time.

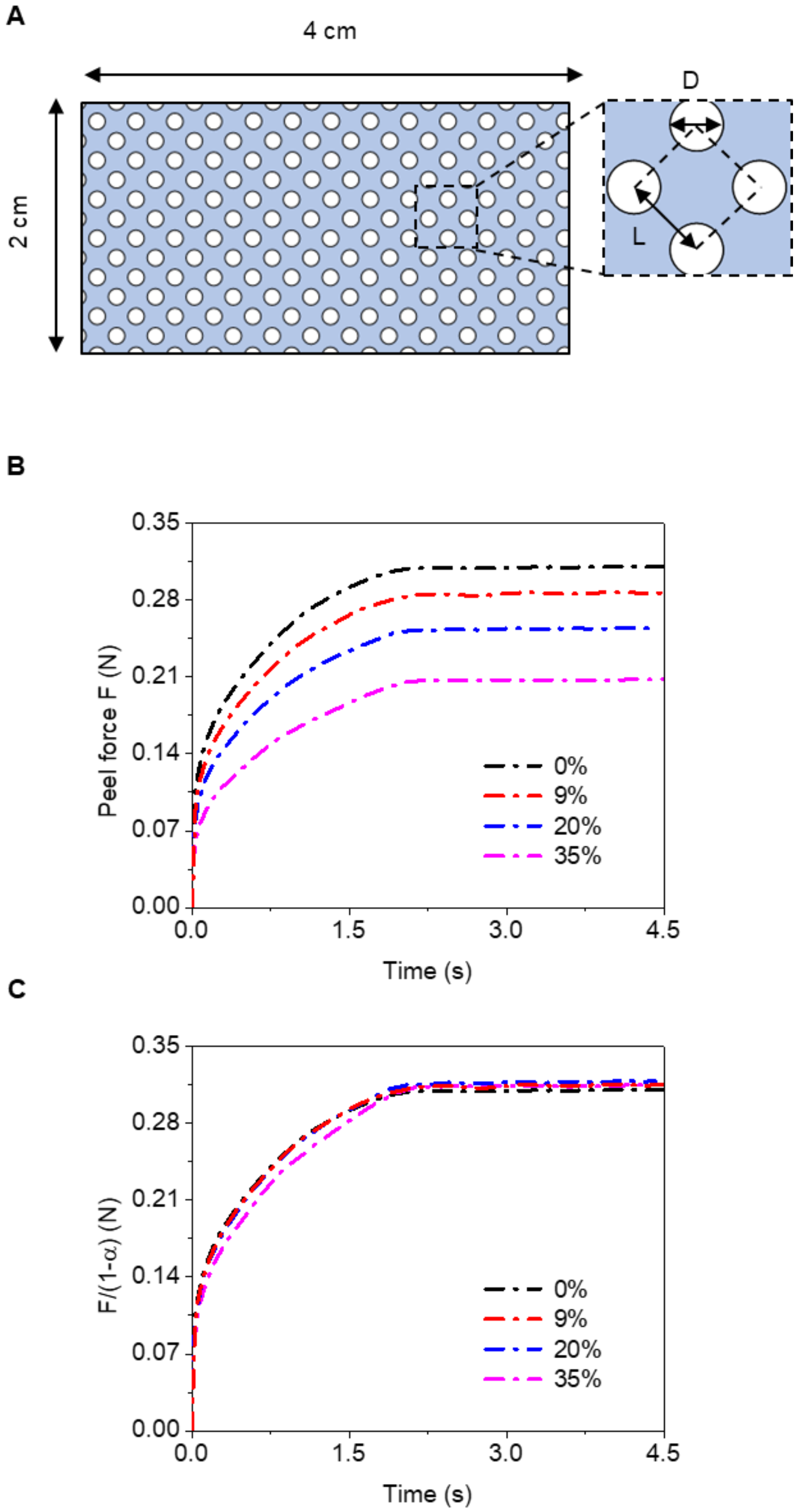

Fig. S23. Effect of a 45 °-rotated perforation pattern in the EES adhesive. (A) The EES adhesive with 45 °-rotated square holes of small diameter D = 200 μm. **(B)** The peel force versus time for different hole area fractions α. **(C)** The normalized peel force F/(1- α) versus time.

A

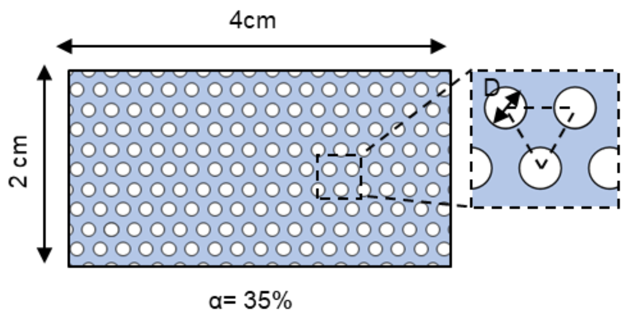

B

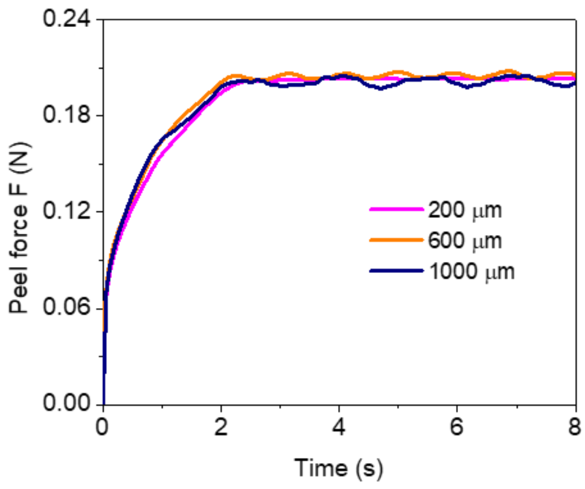

Fig. S24. Effect of a triangular perforation pattern in the EES adhesive, with holes having different diameters. (A) The EES adhesive with holes in a regular triangular pattern, with diameter *D*. The hole area fraction is α = 35 %. (B) The peel force versus time for the holes with different diameters D = 200 µm, 600 µm, and 1000 µm.

A

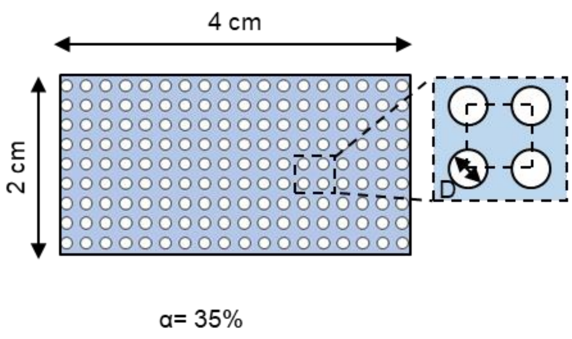

B

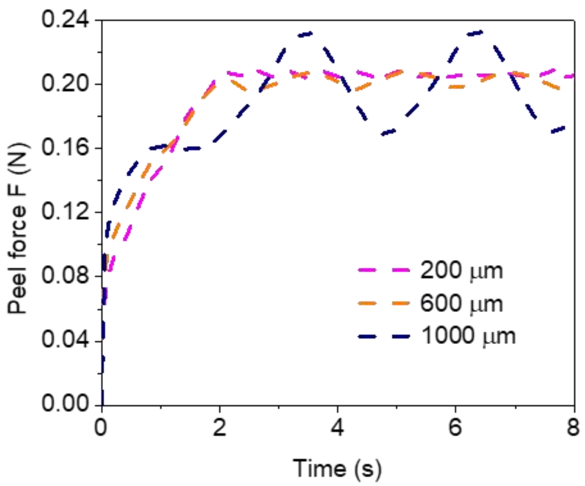

Fig. S25. Effect of a square perforation pattern in the EES adhesive, with holes having different diameters. (A) The EES adhesive with holes in a square pattern, with diameter *D*. The hole area fraction is α = 35 %. **(B)** The peel force versus time for the holes with different diameters D = 200 µm, 600 µm, and 1000 µm.

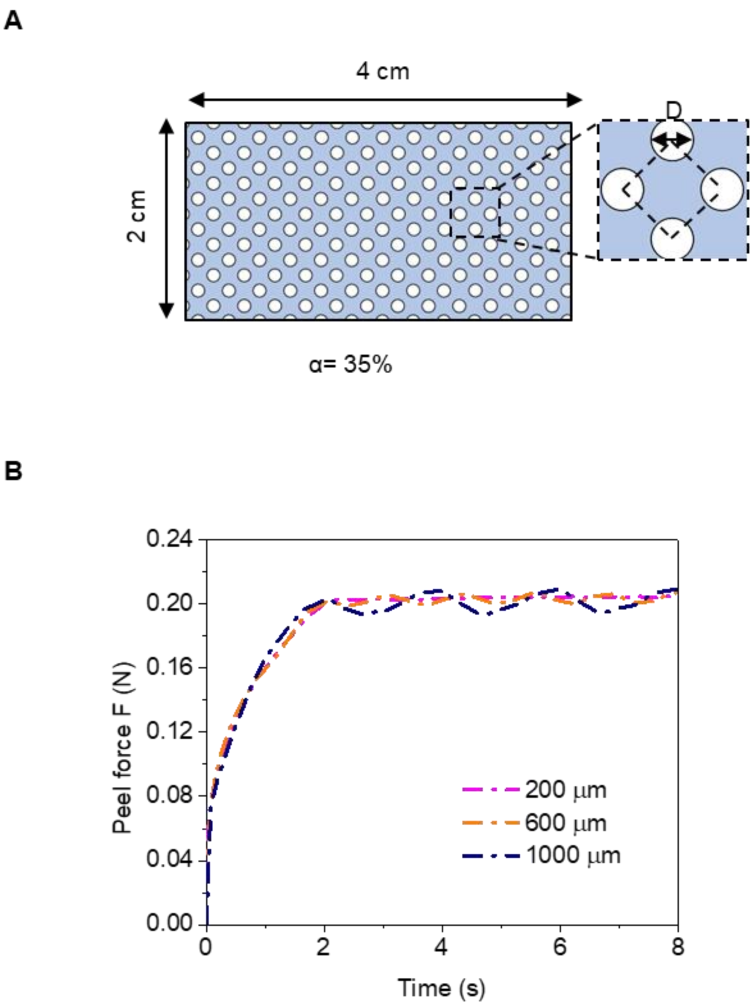

Fig. S26. Effect of a 45 º-rotated perforation pattern in the EES adhesive, with holes having different diameters. (A) The EES adhesive with holes in a square pattern with 45 º rotation, with diameter *D*. The hole area fraction is α = 35 %. **(B)** The peel force versus time for the holes with different diameters D = 200 µm, 600 µm, and 1000 µm.

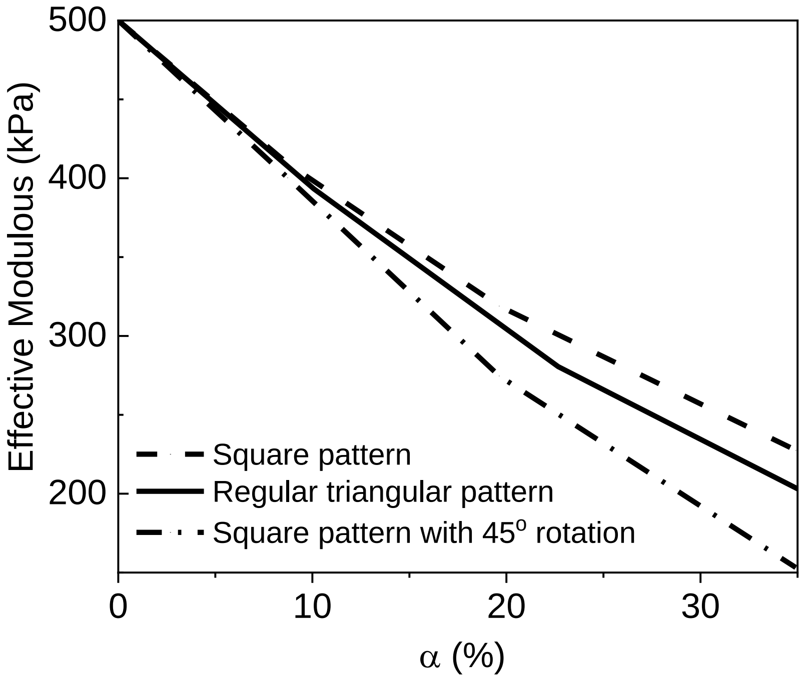

Fig. S27. Effective modulus as a function of hole area fraction, α, for holes with different patterns. The result shows square pattern with 45 rotation renders the minimum effective modulus when compared with hole area fraction.

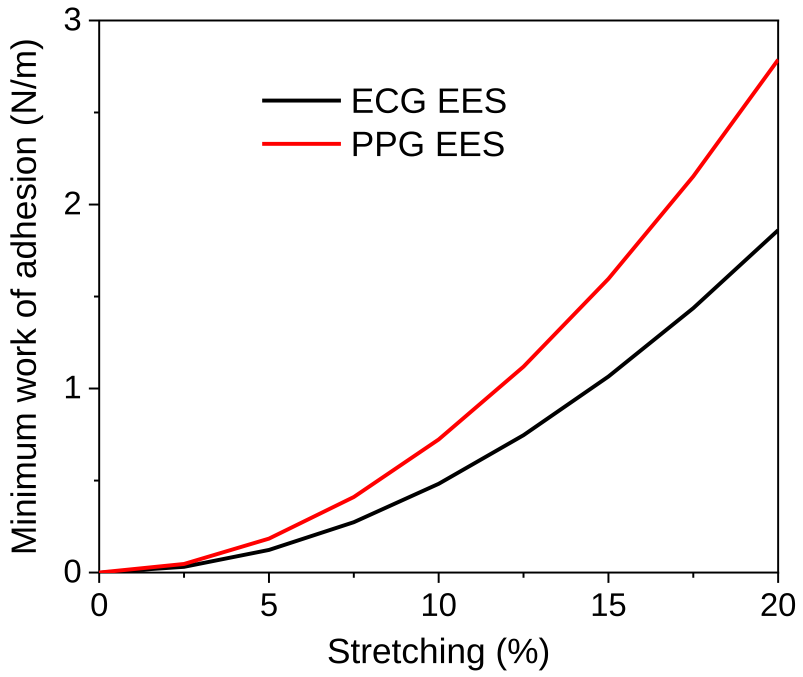

Fig. S28. Plot of minimum work of adhesion versus stretching of the ECG EES and the PPG EES. Minimum work of adhesion strength of the ECG EES (black) and the PPG EES (red) when stretched up to 20 %.

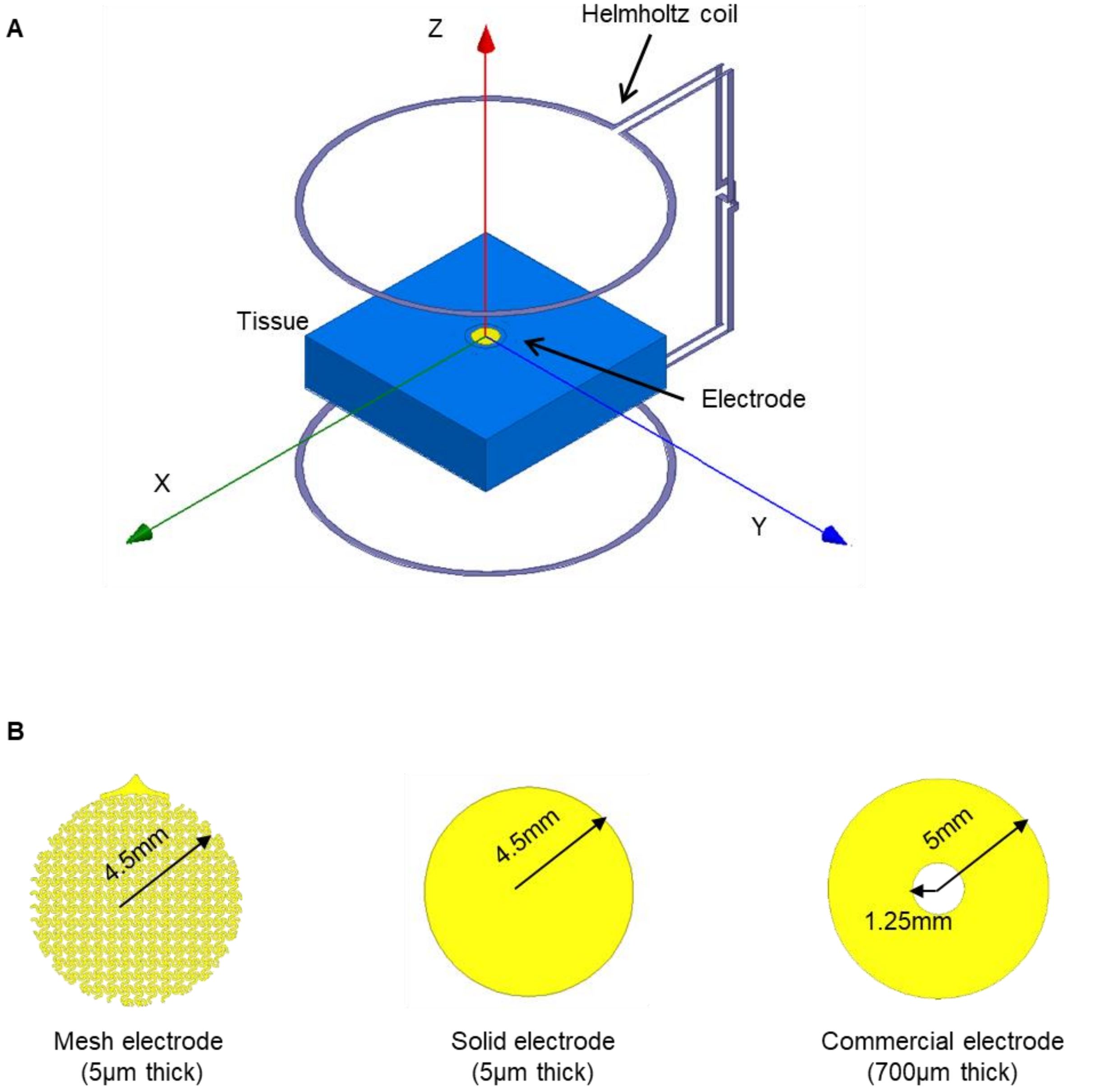

Fig. S29. Modeling of electromagnetic characteristics of the electrodes. (A) Schematic illustration of the electromagnetic simulation model composed of an electrode on the skin and a Helmholtz coil. **(B)** Electrodes with different structures including mesh (geometry for the ECG EES electrodes), solid (i.e. no mesh) and commercial NICU electrodes.

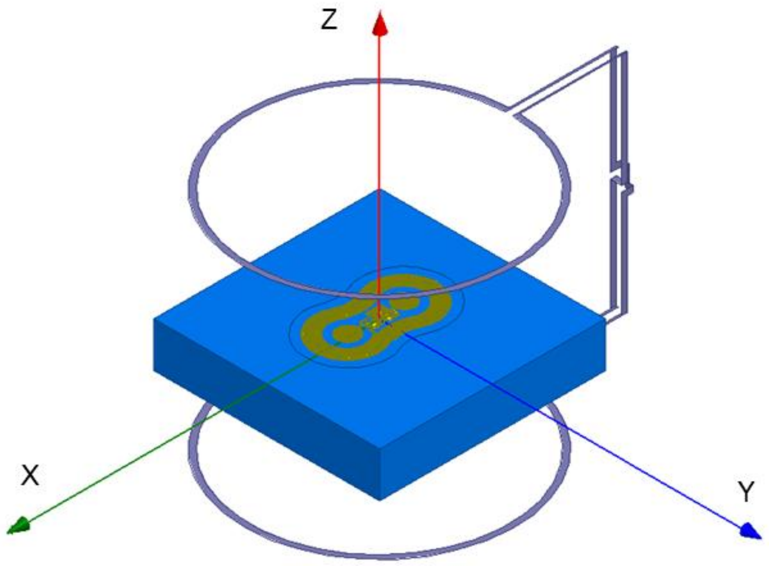

Fig. S30. Modeling of electromagnetic characteristics of the ECG EES. Schematic illustration of the electromagnetic simulation model composed of the ECG EES on the skin and a Helmholtz coil.

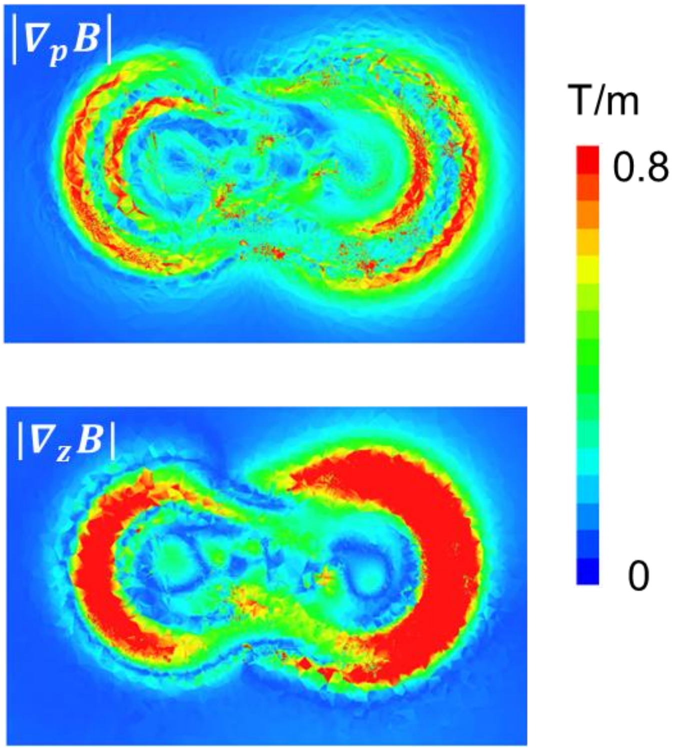

Fig. S31. Computation result for the distributions of the magnetic field density associated with the ECG EES at a frequency of 150 MHz. (Top) In-plane and (bottom) out-of-plane gradients of the magnetic field density induced by the ECG EES.

A

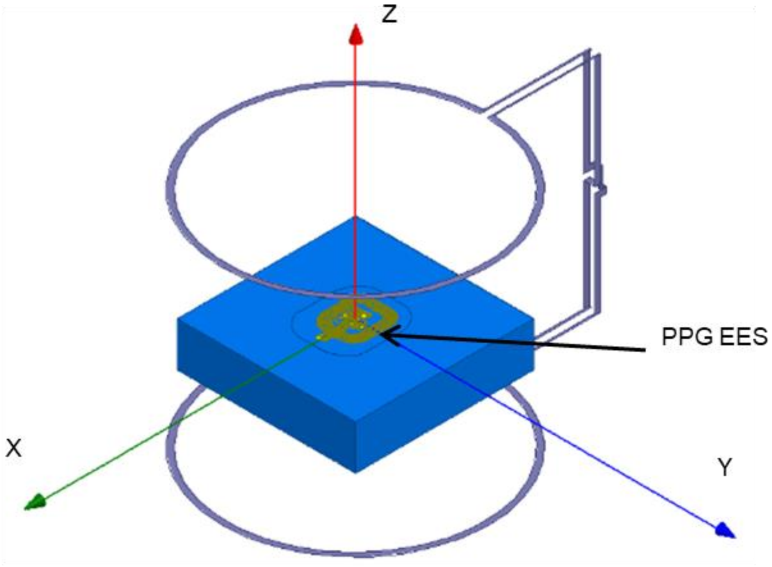

B

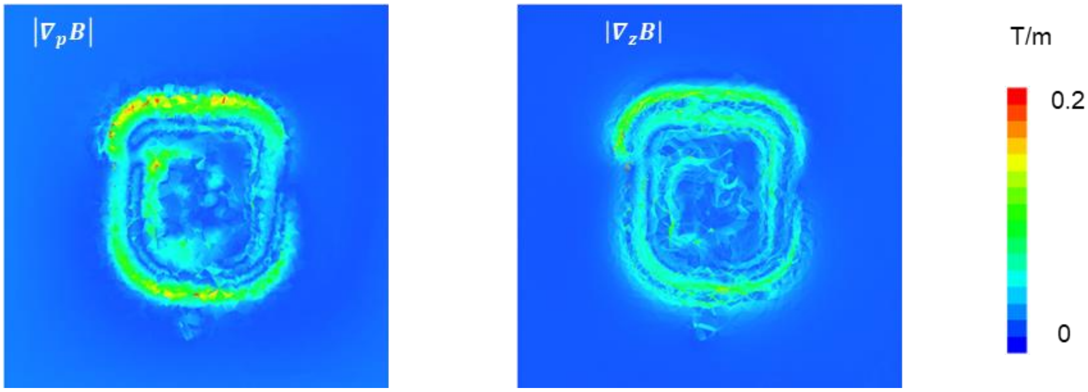

Fig. S32. Modeling of the electromagnetic characteristics of the PPG EES. (A) Schematic illustration of the electromagnetic simulation model composed of the ECG EES on the skin and a Helmholtz coil. **(B)** The in-plane and out-of-plane gradients of magnetic field density induced by a complete PPG EES at 128 MHz.

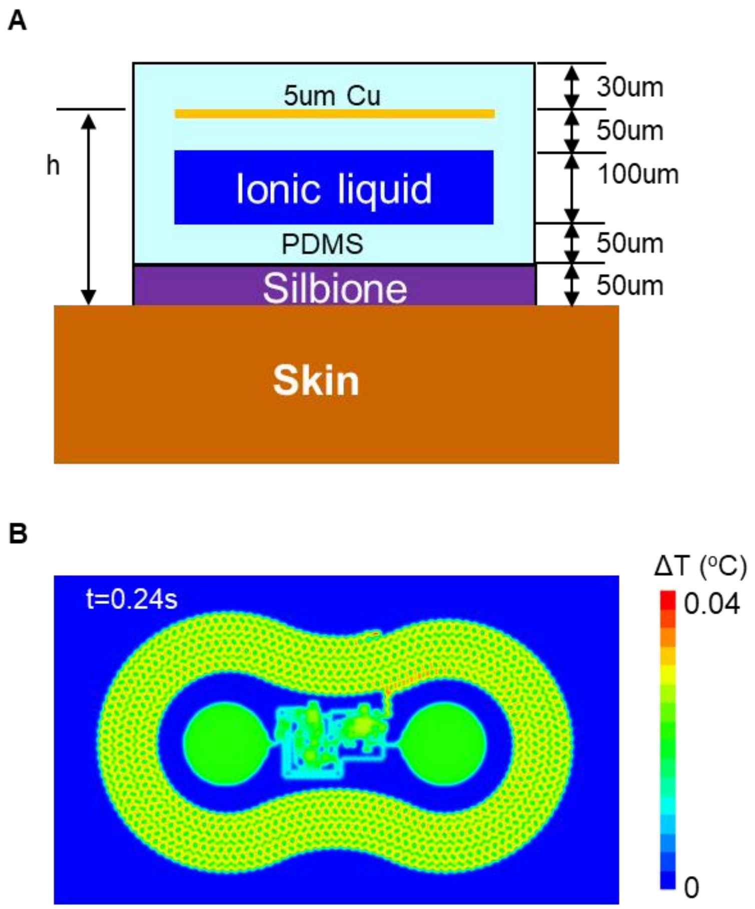

Fig. S33. Modeling of the thermal loads of the ECG EES during an MRI scan. (A) Schematic illustration of the cross-section of the ECG EES. **(B)** The distribution of the temperature change on the skin at 0.24s, which corresponds to the time of the maximum temperature change on the skin for an MRI scan.

A

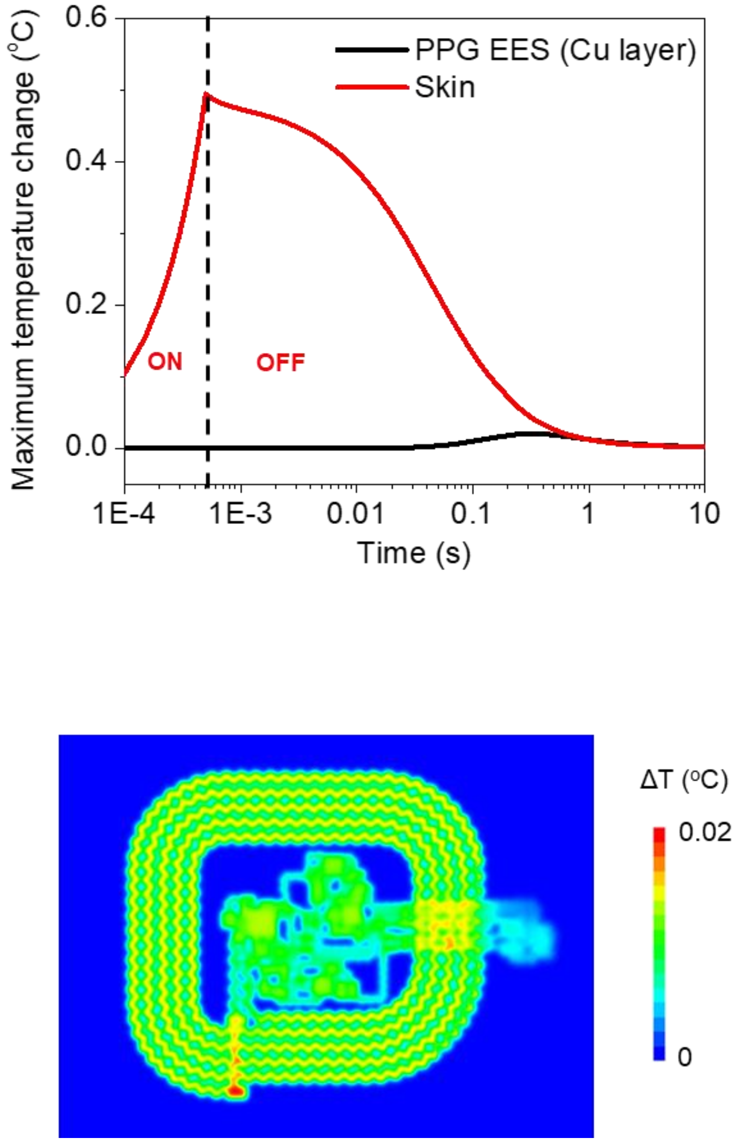

B

Fig. S34. Modeling of the thermal loads of the PPG EES during an MRI scan. (A) The maximum temperature change of the PPG EES and skin versus time for an MRI scan. **(B)** The distribution of the temperature change on the skin at 0.25 s, which corresponds to the time of the maximum temperature change on the skin for an MRI scan.

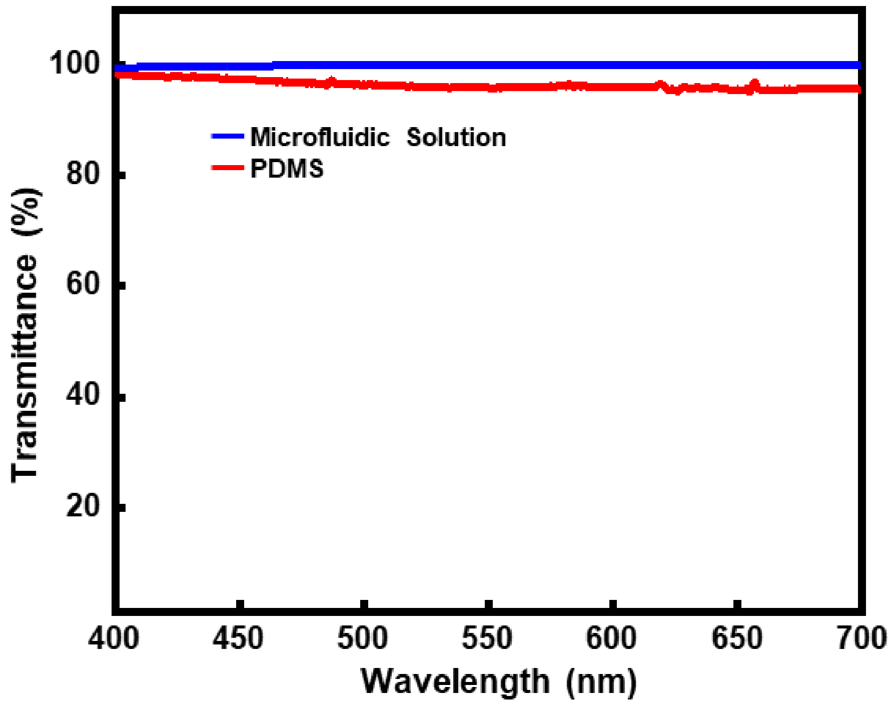

Fig. S35. Plot of the transmittance versus wavelength of the ECG EES. Transparency of the ECG EES with microfluidic solution (blue) is compared with that of PDMS (red).

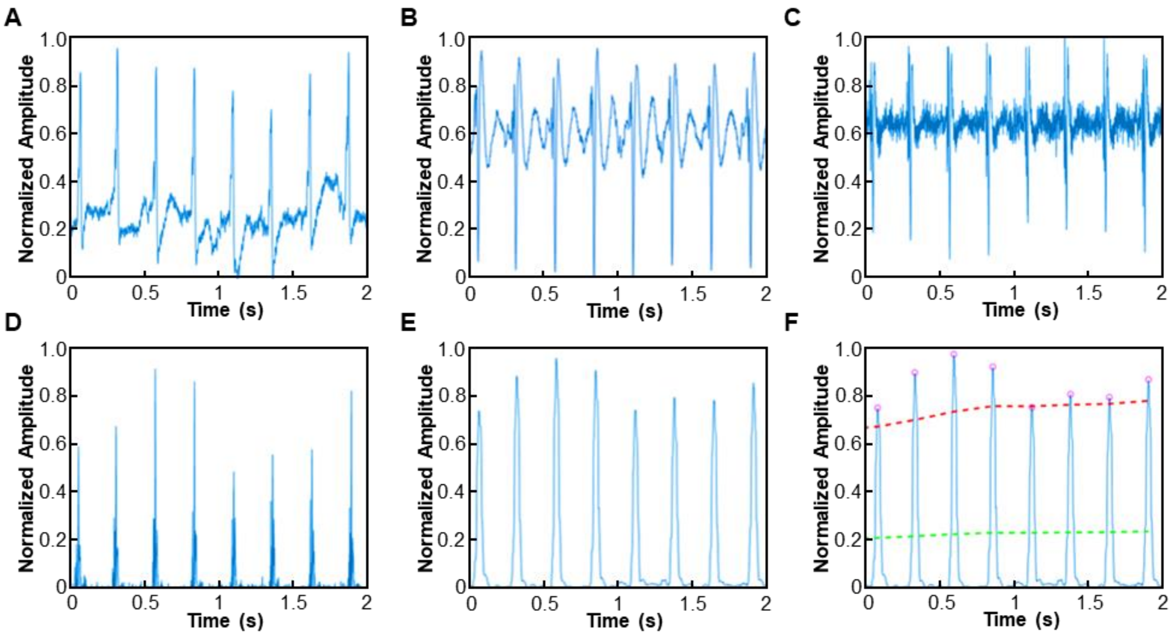

Fig. S36. Streamlined Pan-Tompkins algorithm for peak detection from ECG signals. (A) Raw signal. **(B)** Band-pass filtered signal. **(C)** Differentiation of the signal. **(D)** Squaring the signal. **(E)** Moving average applied to the signal. **(F)** Detected peak with automatically adjusted threshold level.

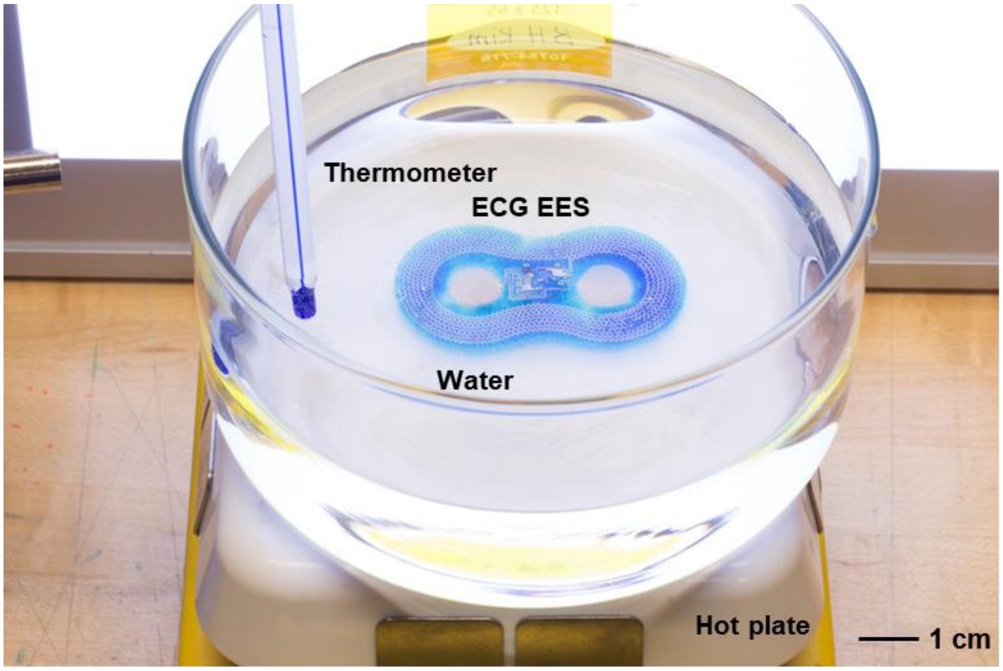

Fig. S37. Calibration of the EES sensors. Experimental setup for calibrating the internal temperature sensor of the ECG EES against a thermometer. The process involves operation under water.

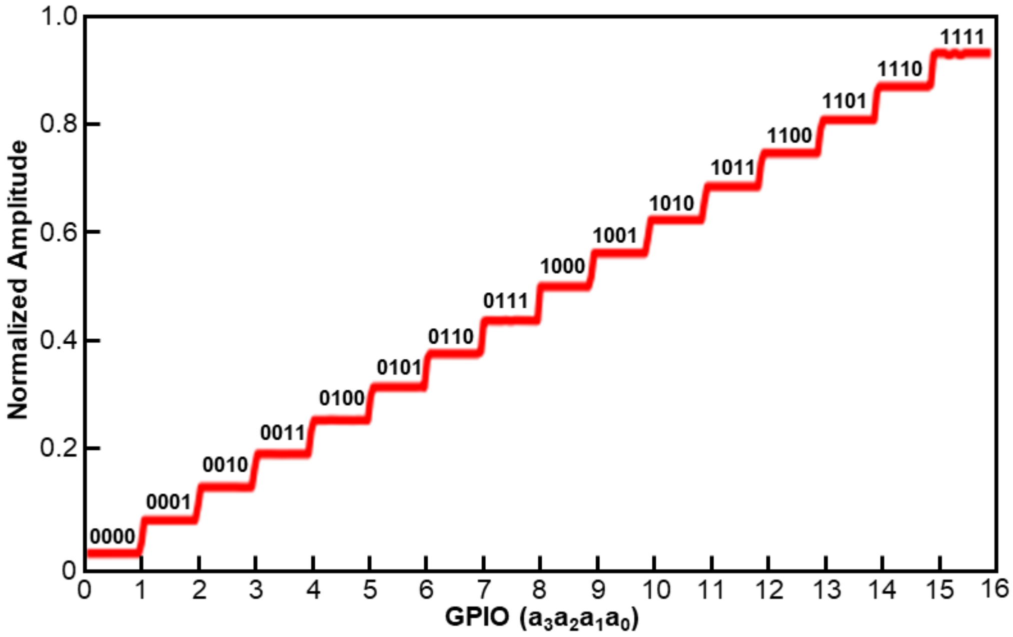

Fig. S38. An exemplary plot showing V+ levels based on general purpose input and output (GPIO) combinations for dynamic baseline control with four channels. Four channel GPIOs implement 16 different levels with equal step size.

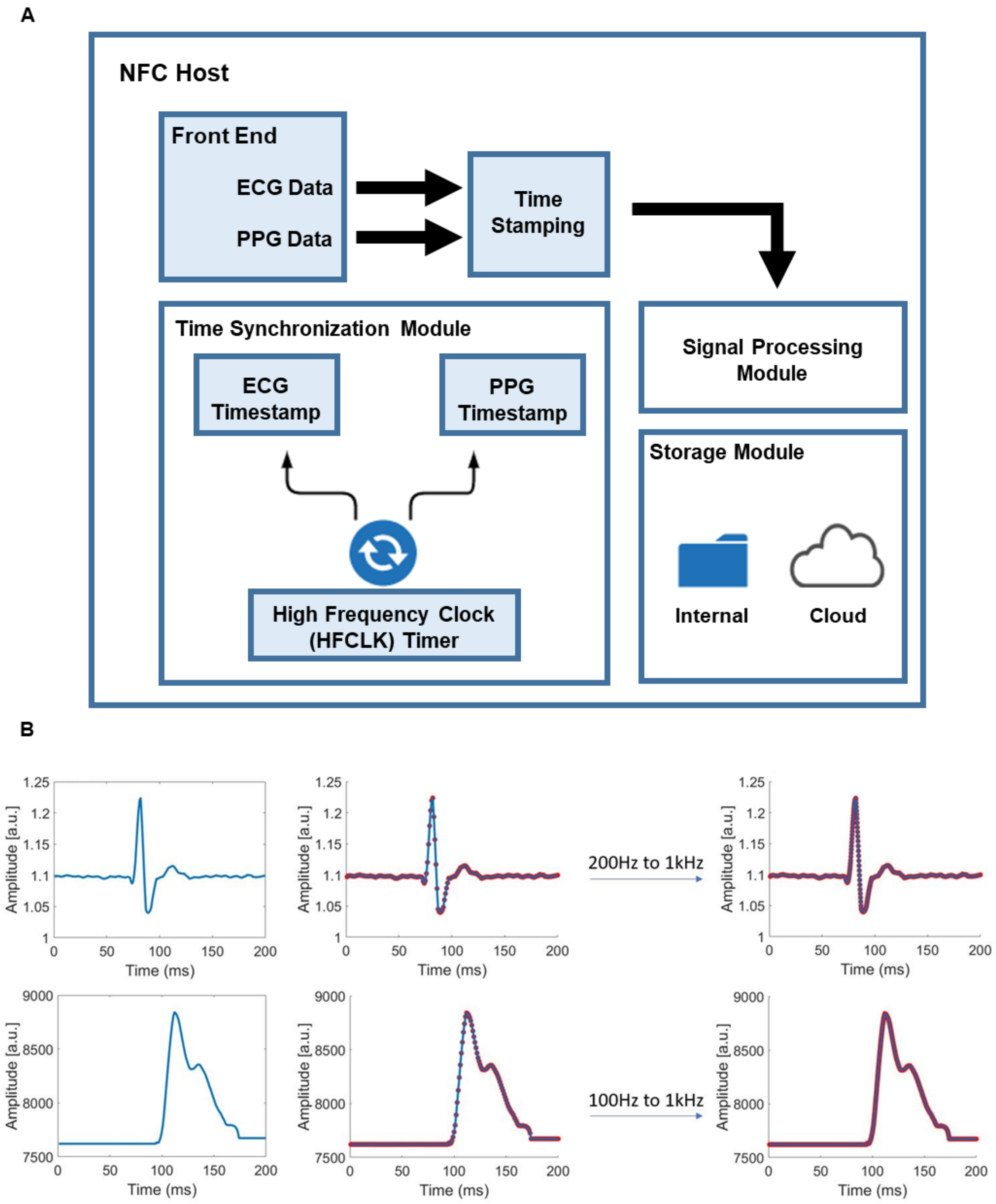

Fig. S39. High level overview of the time synchronization and interpolation scheme. (A) Block diagram of the NFC interface involving synchronization based on the high-frequency clock of reader module, signal processing module explained previously, and storage module in both local and cloud storage. **(B)** Cubic spline interpolation applied to sampled ECG and PPG data.

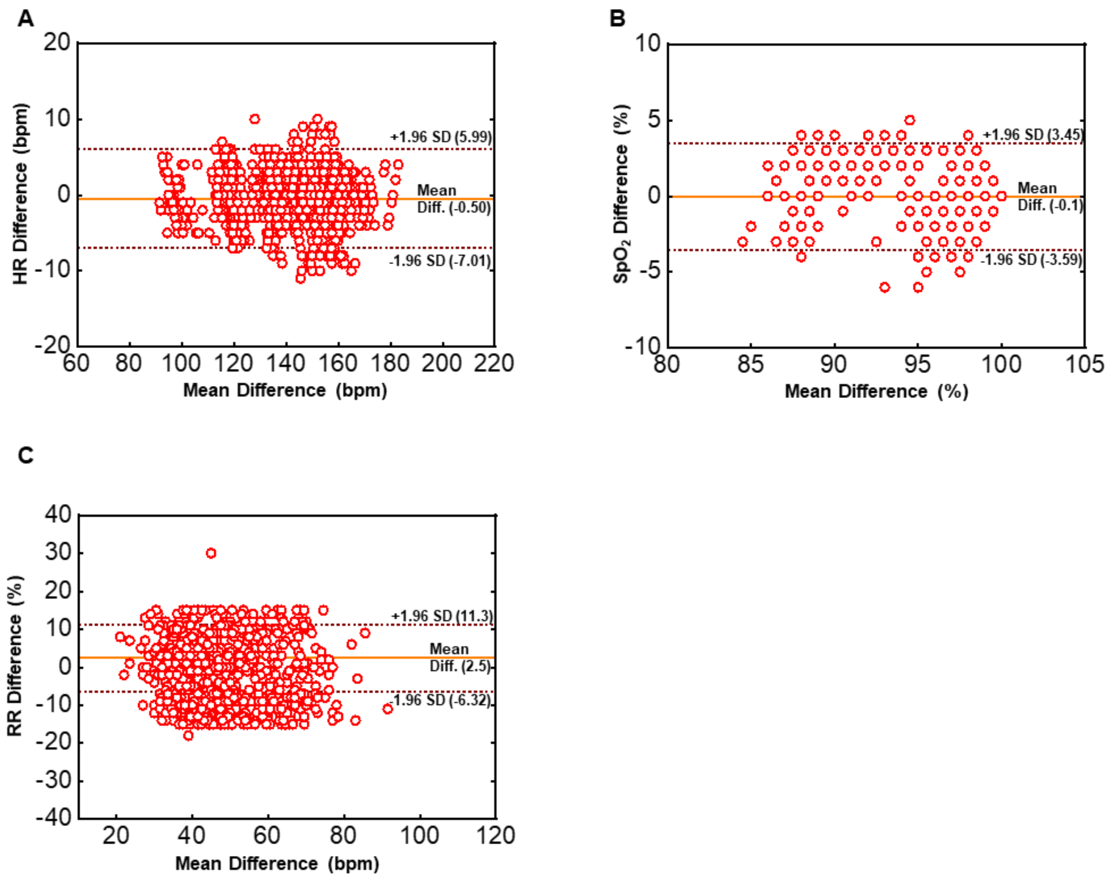

Fig. S40. Bland Altman plots of data collected using the ECG EES and PPG EES and clinical gold standards. Data collected using devices similar to the ECG EES and PPG EES platforms described in the main text, but with on-board power supply, for 18 neonates admitted to the NICU. The mean absolute differences for **(A)** heart rate, **(B)** blood oxygenation, and **(C)** respiratory rate are 0.5 beats/min (standard deviation (SD): 3.3 beats/min), 0.06% (SD: 1.8%), and 2.5 respirations/minute (SD: 4.5 breaths/minute), respectively. No adverse events were noted for any of the subjects.

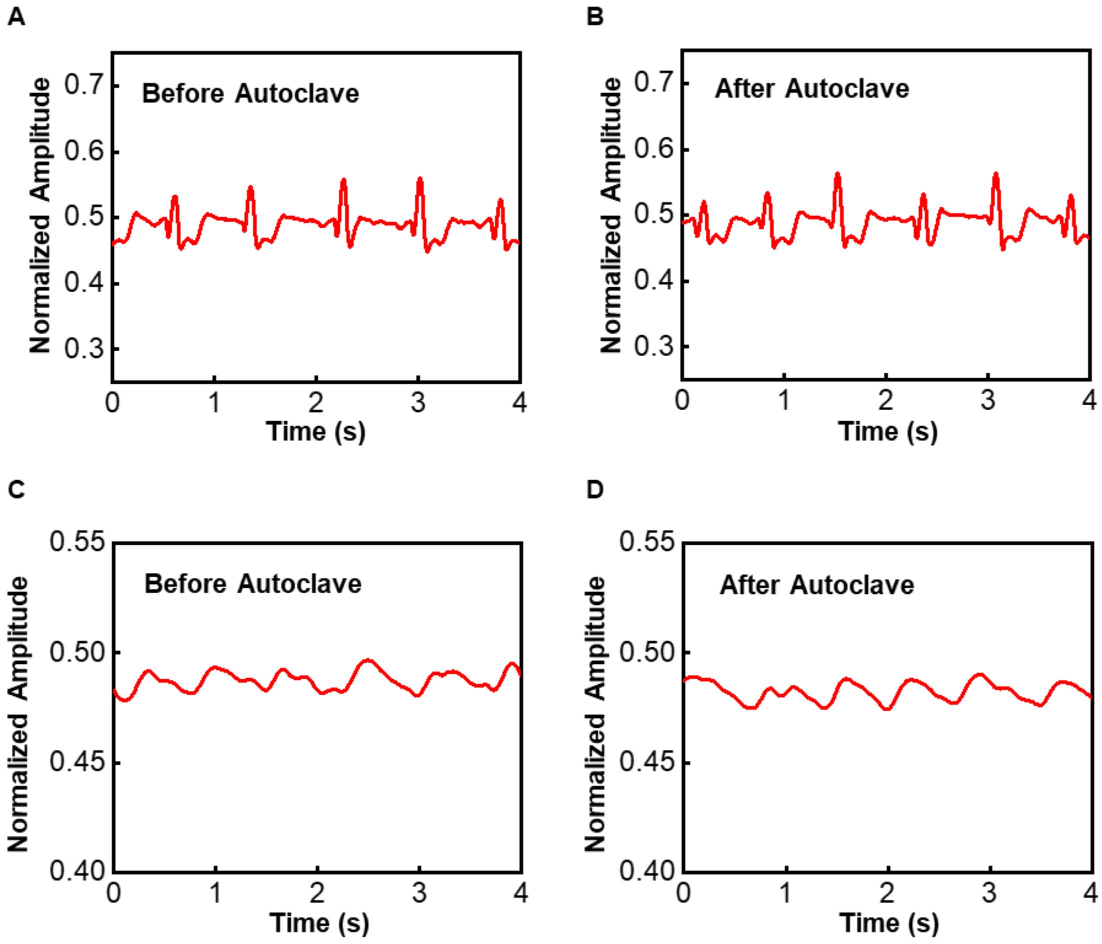

Fig. S41. Demonstration of compatibility of the ECG EES and the PPG EES with autoclave sterilization. (A) ECG signal collected with an ECG EES before autoclave sterilization, **(B)** ECG signal collected with the same device after autoclave sterilization, **(C)** PPG signal collected with a PPG EES before autoclave sterilization and **(D)** PPG signal collected with the same device after autoclave sterilization.

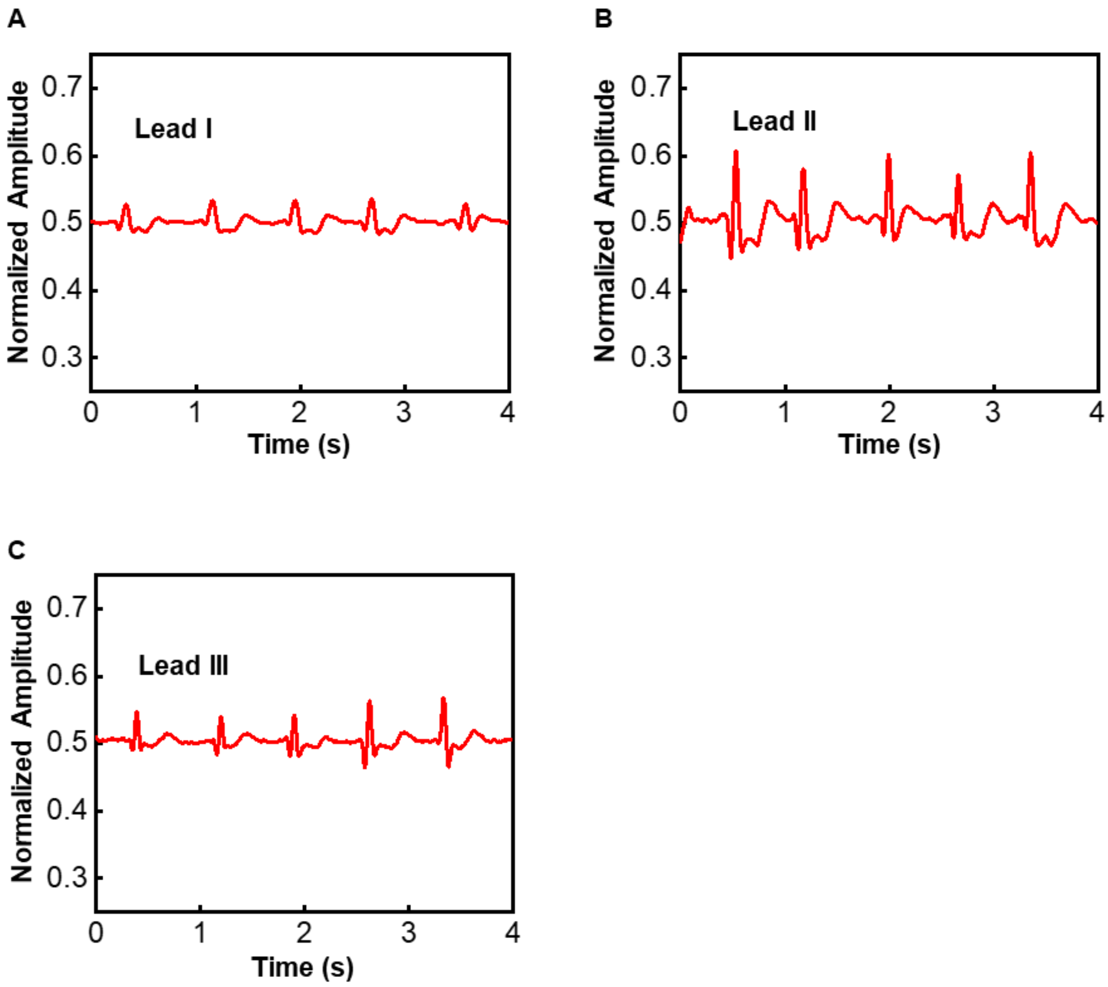

Fig. S42. ECG signal collected with three limb leads configuration. (A) Lead I, **(B)** Lead II, **(C)** Lead III.

A

B

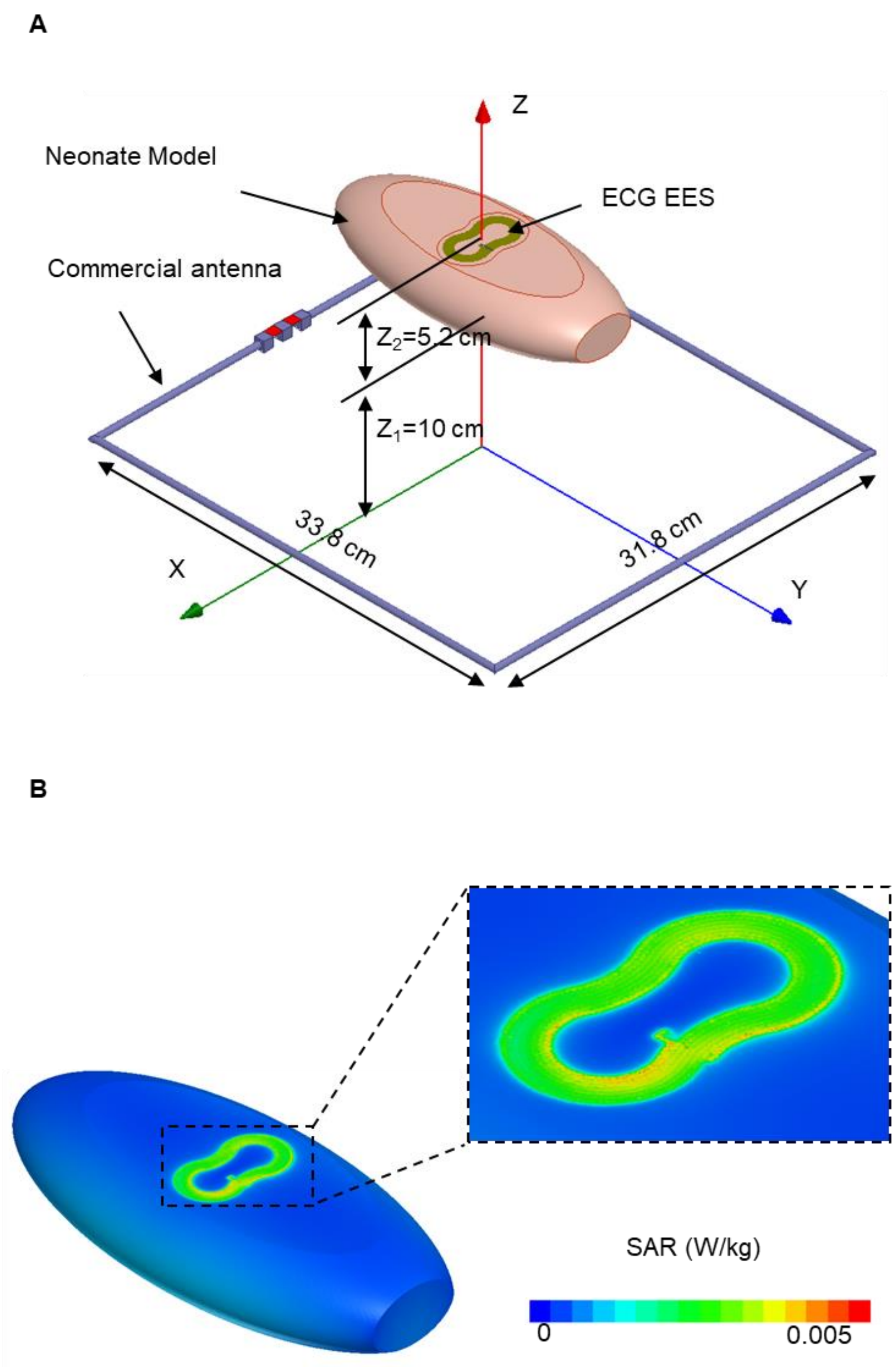

Fig. S43. SAR modeling of the ECG EES for a neonate modeled at 24-week gestational age. The schematic illustration **(A)** of the wireless system for the ECG EES mounted on the neonate with 24-week gestational age, and the SAR distribution **(B)** on the surface of the neonate. The neonate is modeled as an ellipsoid with major (half) axes 30, 50, and 115 mm. Z_1 and Z_2 are the distances between the commercial antenna and the neonate, and the thickness of neonate, respectively. The distance between the neonate and the antenna of the ECG EES is 250 μm.

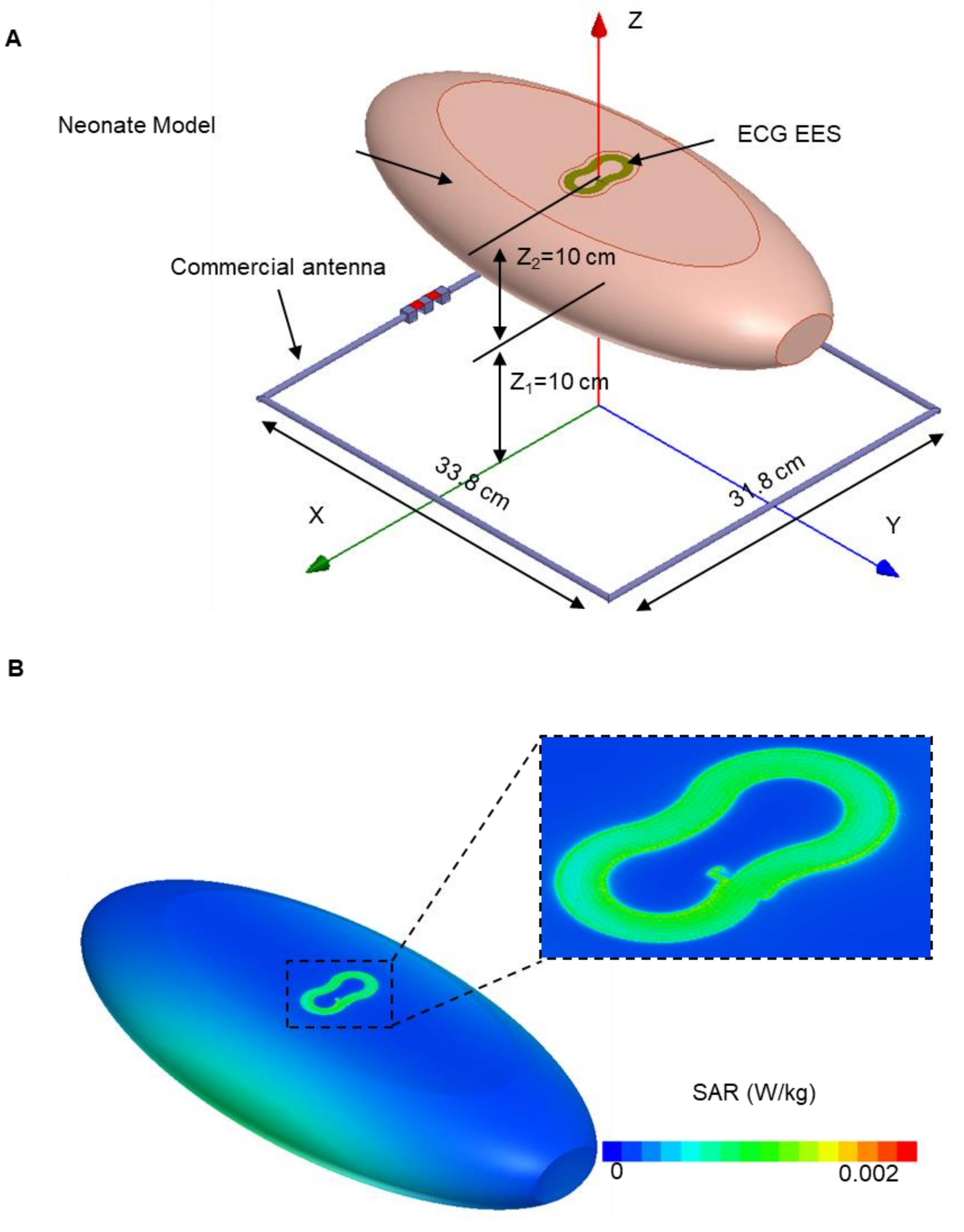

Fig. S44. SAR modeling of the ECG EES for a neonate modeled at 40-week gestational age. The schematic illustration **(A)** of the wireless system for the ECG EES mounted on the neonate with 40-week gestational age, and the SAR distribution **(B)** on the surface of the neonate. The neonate is modeled as an ellipsoid with major (half) axes 55, 90, and 210 mm. Z_1 and Z_2 are the distances between the commercial antenna and the neonate, and the thickness of neonate, respectively. The distance between the neonate and the antenna of the ECG EES is 250 µm.

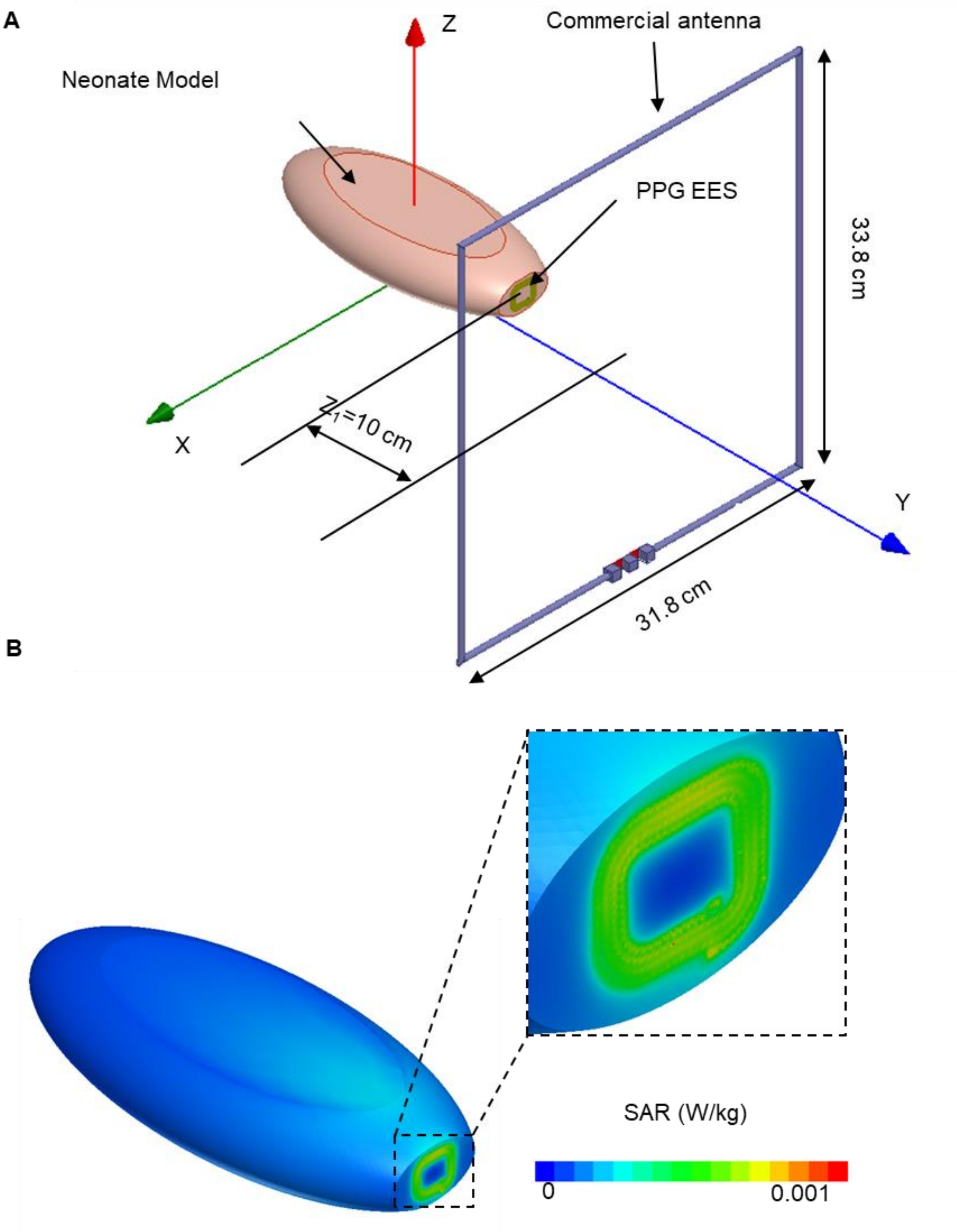

Fig. S45. SAR modeling of the PPG EES for a neonate modeled at 24-week gestational age. The schematic illustration **(A)** of the wireless system for the PPG EES mounted on the neonate with 24-week gestational age, and the SAR distribution **(B)** on the surface of the neonate. The neonate is modeled as an ellipsoid with major (half) axes 30, 50, and 115 mm. Z_1 is the distance between the commercial antenna and the neonate. The distance between the neonate and the antenna of the ECG EES is 250 µm.

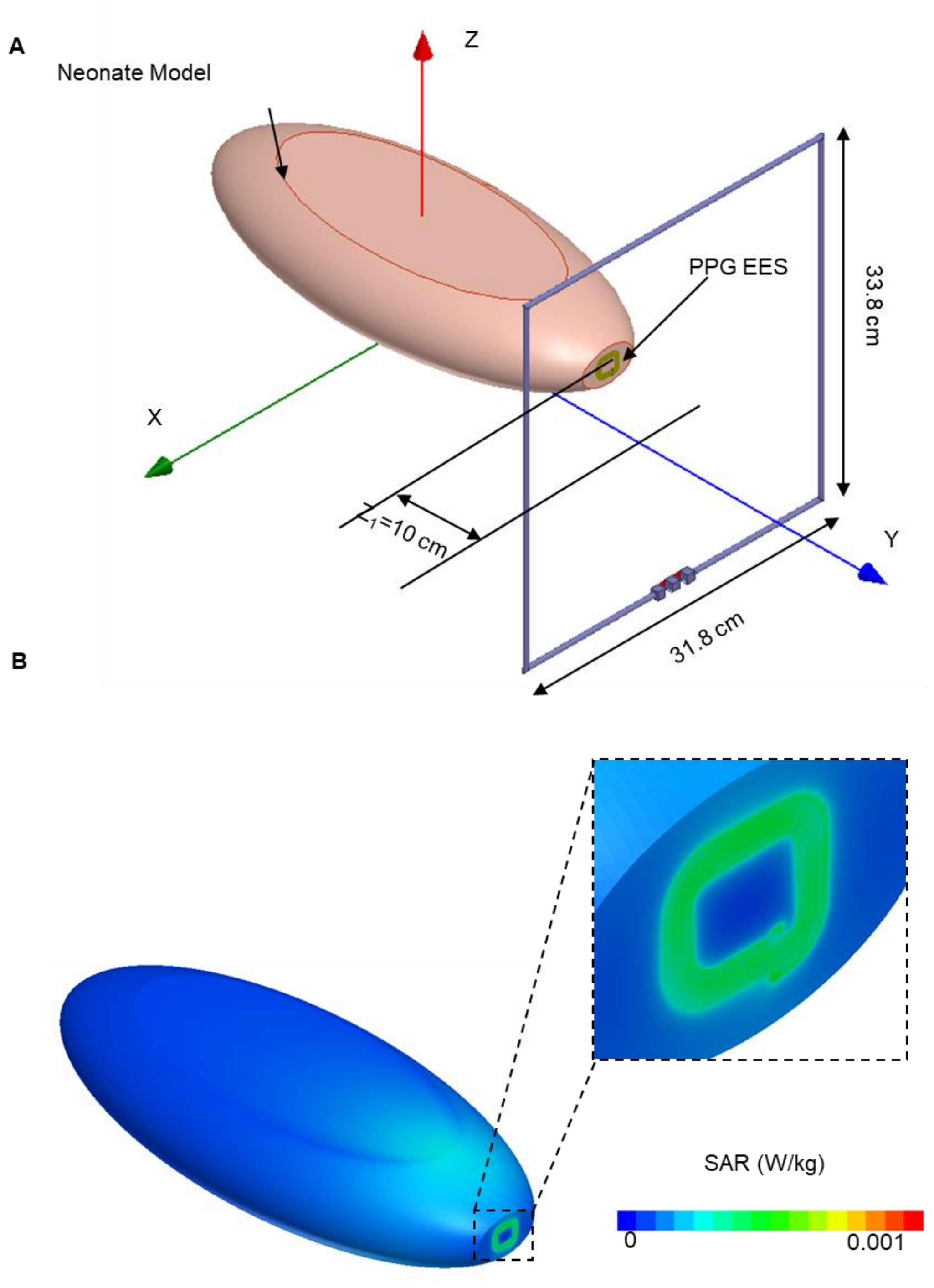

Fig. S46. SAR modeling of the PPG EES for a neonate modeled at 40-week gestational age. The schematic illustration **(A)** of the wireless system for the PPG EES mounted on the neonate with 40-week gestational age, and the SAR distribution **(B)** on the surface of the neonate. The neonate is modeled as an ellipsoid with major (half) axes 55, 90, and 210 mm. Z_1 is the distance between the commercial antenna and the neonate. The distance between the neonate and the antenna of the ECG EES is 250 µm.

A

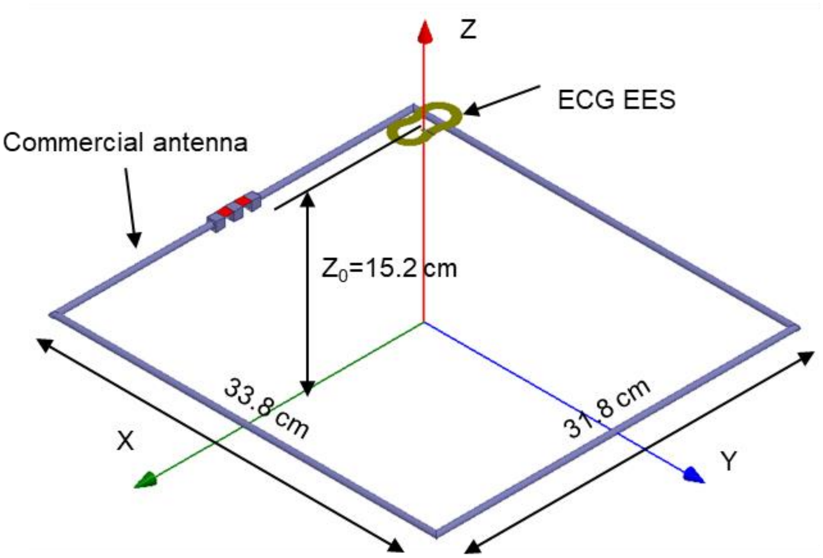

B

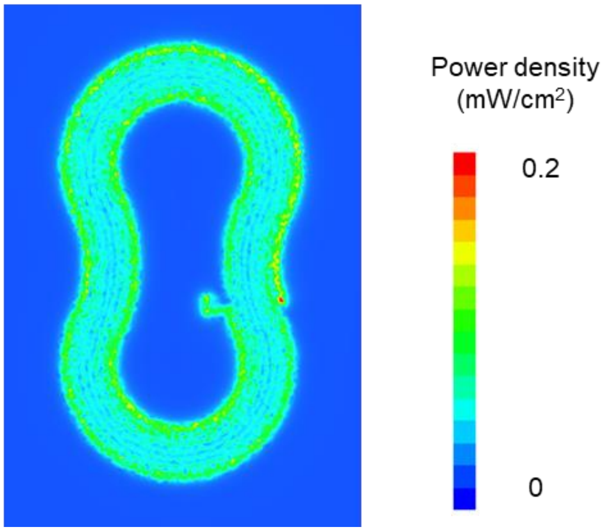

Fig. S47. MPE modeling of the ECG EES. The schematic illustration **(A)** of the wireless system with the ECG EES and the commercial coil, and the power density distribution **(B)** in the plane 250 µm underneath of the antenna of the ECG EES. Z_0 is the distance between the commercial antenna and the ECG EES.

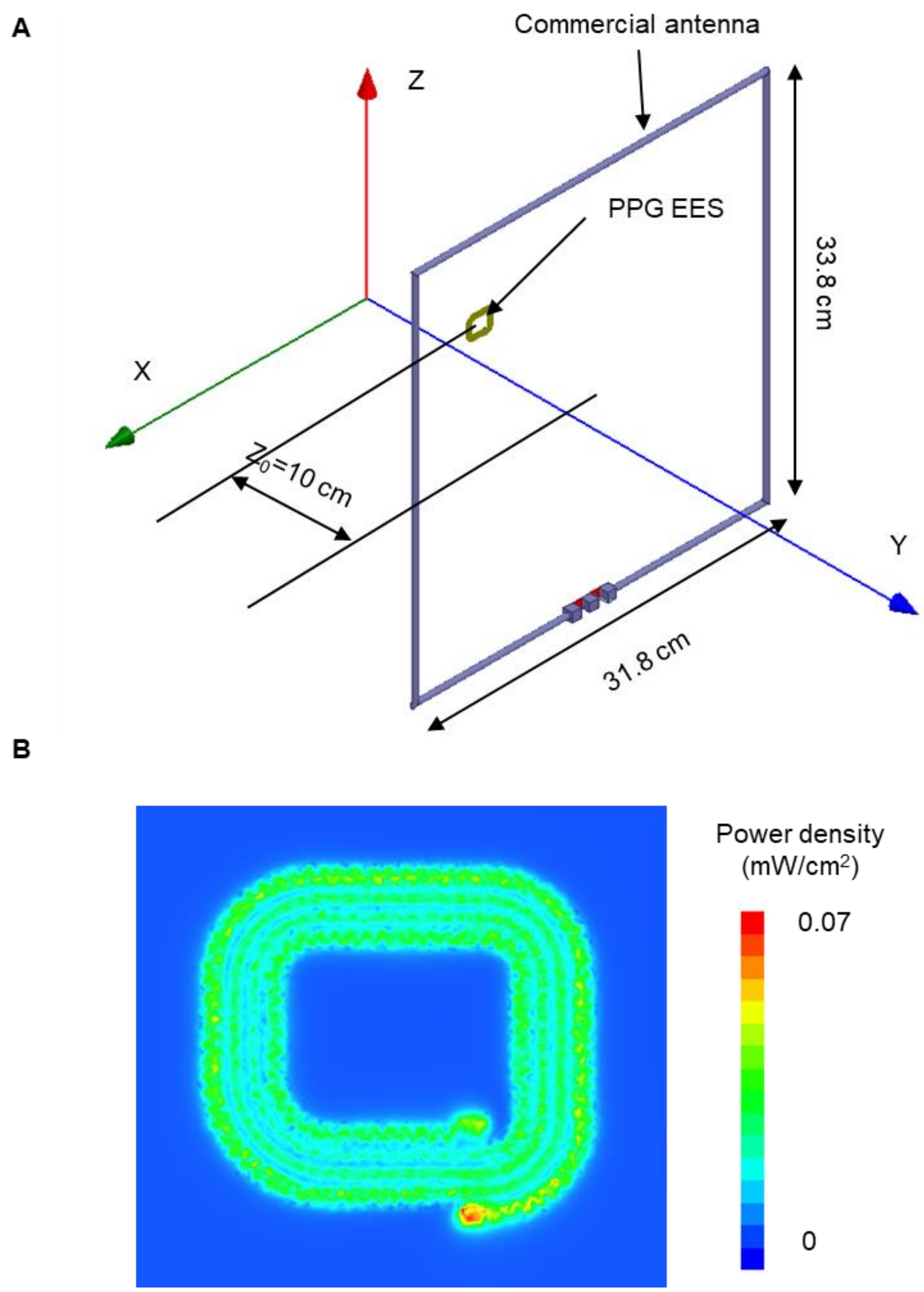

Fig. S48. MPE modeling of the PPG EES. The schematic illustration **(A)** of the wireless system with the PPG EES and the commercial coil, and the power density distribution **(B)** in the plane 250 µm underneath of the antenna of the PPG EES. Z_0 is the distance between the commercial antenna and the PPG EES.

Clinical Validation of Wireless, Battery-Free ECG EES and PPG EES System in the Neonatal Intensive Care Unit		
**Subject**	**Gestational Age (Weeks)**	**Ethnicity**
1	28	Hispanic
2	29	Caucasian
3	40	Asian

Clinical Validation of Wireless, Battery-Embedded ECG EES and PPG EES System in the Neonatal Intensive Care Unit		
**Subject**	**Gestational Age (Weeks)**	**Ethnicity**
1	41	Hispanic
2	34	South Asian
3	40	Caucasian
4	39	African American
5	36	African American
6	35	Caucasian
7	41	Caucasian
8	38	South Asian
9	37	Caucasian
10	38	Caucasian
11	34	Caucasian
12	34	Caucasian
13	40	Caucasian
14	33	Caucasian
15	33	Caucasian
16	28	Hispanic
17	36	African American
18	39	African American

Table S1. Characteristics of neonates who participated in this study. The table shows gestational age and ethnicity of neonates admitted in the NICU and tested with the battery-free, dual EES system using both battery-free modality (n = 3, top) as well as battery-powered version (n = 18, below).

<table>
<tr><th>Component</th><th colspan="2">Cost Determination</th></tr>
<tr><td>Passive Components</td><td colspan="2">• $0.30 per sensor given about 30 passive components per device at $0.01 per passive component</td></tr>
<tr><td>Active Components</td><td colspan="2">• $3.79 per RF unit
• $3.19 for the instrumentation amplifier (only ECG EES)
• $4.7 for photodiode / LEDs / rectifier / buck converter (only PPG EES)
• $1.25 for the voltage feedback amplifier</td></tr>
<tr><td rowspan="3">Silicon Encapsulation
(PDMS, Silbione)</td><td>PDMS</td><td>$ 127.64 for 100 devices
(10 g / device)
($ 63.82 / 0.5 kg)</td></tr>
<tr><td>Silbione
High Tack Silicone
Gel. A-4717-1</td><td>$ 88.10 for 100 devices
(5 g / device)
($ 39.95 / 8 oz)</td></tr>
<tr><td colspan="2">Subtotal : $ 215.74 for 100 devices / $2.16 per device</td></tr>
<tr><td>Ionic Liquid</td><td colspan="2">• $ 2.58 per devices (0.7 g of ionic fluid per device)</td></tr>
<tr><td>Dual Copper Sheet</td><td colspan="2">• $ 4.60 per device ($ 947 / 50 Sheets)</td></tr>
<tr><td rowspan="3">Fabrication Costs
(Photolithography, E-beam, RIE)</td><td>Cleanroom ($ 18 / hr)</td><td>$ 180 for 100 devices
(10 devices / hr)</td></tr>
<tr><td>Harrick Plasma Cleaner
($ 19 / hr)</td><td>$ 475 for 100 devices
(4 devices / hr)</td></tr>
<tr><td colspan="2">Subtotal : $6.55 per device</td></tr>
<tr><td>Total</td><td colspan="2">$17.96 (ECG EES) / $19.47 (PPG EES) per device</td></tr>
</table>

Table S2. **Table of the estimated costs of the ECG EES and PPG EES.** The estimation renders $17.96 and $19.47 for the ECG EES and PPG EES, respectively.

Table S3 (Provided as a separate Excel file).
Validation of vital signs from the EES platform compared against the gold standard (Adult Subjects).

Table S4 (Provided as a separate Excel file).
Validation of vital signs from the EES platform compared against the gold standard (Neonate Subjects).

References

1. W. Harrison, D. Goodman, Epidemiologic trends in neonatal intensive care, 2007-2012. *JAMA Pediatr.* **169**, 855–862 (2015). doi:10.1001/jamapediatrics.2015.1305 Medline

2. P. H. Cartlidge, P. E. Fox, N. Rutter, The scars of newborn intensive care. *Early Hum. Dev.* **21**, 1–10 (1990). doi:10.1016/0378-3782(90)90105-R Medline

3. C. Lund, Medical adhesives in the NICU. *Newborn Infant Nurs. Rev.* **14**, 160–165 (2014). doi:10.1053/j.nainr.2014.10.001

4. A. C. Tottman, J. M. Alsweiler, F. H. Bloomfield, J. E. Harding, Presence and pattern of scarring in children born very preterm. *Arch. Dis. Child Fetal Neonatal Ed.* **103**, F277–F279 (2018). doi:10.1136/archdischild-2016-311999

5. S. Bouwstra, W. Chen, L. Feijs, S. B. Oetomo, in *2009 Sixth International Workshop on Wearable and Implantable Body Sensor Networks* (IEEE, 2009), pp. 162–167. doi:10.1109/BSN.2009.40

6. O. Sharma, S. N. Lewis, U. Telang, L. D'Almeida, L. E. S. Lewis, Design of a Bluetooth Enabled Health Monitoring System for Infants Using Wearable Technology. *J. Adv. Res. Dyn. Contrl. Syst.* **15**, 887–894 (2017). http://jardcs.org/papers/v9/sp/20181075.pdf

7. K. M. McLane, K. Bookout, S. McCord, J. McCain, L. S. Jefferson, The 2003 National Pediatric Pressure Ulcer and Skin Breakdown Prevalence Survey: A multisite study. *J. Wound Ostomy Continence Nurs.* **31**, 168–178 (2004). doi:10.1097/00152192-200407000-00004 Medline

8. D. H. Kim, N. Lu, R. Ma, Y.-S. Kim, R.-H. Kim, S. Wang, J. Wu, S. M. Won, H. Tao, A. Islam, K. J. Yu, T. I. Kim, R. Chowdhury, M. Ying, L. Xu, M. Li, H.-J. Chung, H. Keum, M. McCormick, P. Liu, Y.-W. Zhang, F. G. Omenetto, Y. Huang, T. Coleman, J. A. Rogers, Epidermal electronics. *Science* **333**, 838–843 (2011). doi:10.1126/science.1206157 Medline

9. K. Harris, A. Elias, H.-J. Chung, Flexible electronics under strain: A review of mechanical characterization and durability enhancement strategies. *J. Mater. Sci.* **51**, 2771–2805 (2016). doi:10.1007/s10853-015-9643-3

10. W. Gao, S. Emaminejad, H. Y. Y. Nyein, S. Challa, K. Chen, A. Peck, H. M. Fahad, H. Ota, H. Shiraki, D. Kiriya, D.-H. Lien, G. A. Brooks, R. W. Davis, A. Javey, Fully integrated wearable sensor arrays for multiplexed in situ perspiration analysis. *Nature* **529**, 509–514 (2016). doi:10.1038/nature16521 Medline

11. J. A. Walsh 3rd, E. J. Topol, S. R. Steinhubl, Novel wireless devices for cardiac monitoring. *Circulation* **130**, 573–581 (2014). doi:10.1161/CIRCULATIONAHA.114.009024 Medline

12. J. P. J. Halcox, K. Wareham, A. Cardew, M. Gilmore, J. P. Barry, C. Phillips, M. B. Gravenor, Assessment of remote heart rhythm sampling using the AliveCor heart monitor to screen for atrial fibrillation: The REHEARSE-AF study. *Circulation* **136**, 1784–1794 (2017). doi:10.1161/CIRCULATIONAHA.117.030583 Medline

13. K.-I. Jang, K. Li, H. U. Chung, S. Xu, H. N. Jung, Y. Yang, J. W. Kwak, H. H. Jung, J. Song, C. Yang, A. Wang, Z. Liu, J. Y. Lee, B. H. Kim, J.-H. Kim, J. Lee, Y. Yu, B. J. Kim, H. Jang, K. J. Yu, J. Kim, J. W. Lee, J.-W. Jeong, Y. M. Song, Y. Huang, Y. Zhang, J. A. Rogers, Self-assembled three dimensional network designs for soft electronics. *Nat. Commun.* **8**, 15894 (2017). doi:10.1038/ncomms15894 Medline

14. Y. Ma, M. Pharr, L. Wang, J. Kim, Y. Liu, Y. Xue, R. Ning, X. Wang, H. U. Chung, X. Feng, J. A. Rogers, Y. Huang, Soft Elastomers with Ionic Liquid-Filled Cavities as Strain Isolating Substrates for Wearable Electronics. *Small* **13**, 1602954 (2017). doi:10.1002/smll.201602954 Medline

15. V. Coskun, B. Ozdenizci, K. Ok, The survey on near field communication. *Sensors* **15**, 13348–13405 (2015). doi:10.3390/s150613348 Medline

16. S. Majumder, T. Mondal, M. J. Deen, Wearable sensors for remote health monitoring. *Sensors* **17**, 130 (2017). doi:10.3390/s17010130 Medline

17. W. Dang, L. Manjakkal, W. T. Navaraj, L. Lorenzelli, V. Vinciguerra, R. Dahiya, Stretchable wireless system for sweat pH monitoring. *Biosens. Bioelectron.* **107**, 192–202 (2018). doi:10.1016/j.bios.2018.02.025 Medline

18. M. Visscher, T. Taylor, Pressure ulcers in the hospitalized neonate: Rates and risk factors. *Sci. Rep.* **4**, 7429 (2014). doi:10.1038/srep07429 Medline

19. C. H. Lund, J. A. Tucker, in *Neonatal Skin: Structure and Function*, S.B. Hoath, H. I. Maibach, Eds. (Dekker, ed. 2, 2003), pp. 299–324.

20. N. J. Evans, N. Rutter, Development of the epidermis in the newborn. *Biol. Neonate* **49**, 74–80 (1986). doi:10.1159/000242513 Medline

21. N. Rutter, The immature skin. *Br. Med. Bull.* **44**, 957–970 (1988). doi:10.1093/oxfordjournals.bmb.a072303 Medline

22. A. N. Gent, G. R. Hamed, Peel mechanics. *J. Adhes.* **7**, 91–95 (1975). doi:10.1080/00218467508075041

23. C. D. Smyser, H. Kidokoro, T. E. Inder, Magnetic resonance imaging of the brain at term equivalent age in extremely premature neonates: To scan or not to scan? *J. Paediatr. Child Health* **48**, 794–800 (2012). doi:10.1111/j.1440-1754.2012.02535.x Medline

24. L. Melbourne, T. Chang, J. Murnick, I. Zaniletti, P. Glass, A. N. Massaro, Clinical impact of term-equivalent magnetic resonance imaging in extremely low-birth-weight infants at a regional NICU. *J. Perinatol.* **36**, 985–989 (2016). doi:10.1038/jp.2016.116 Medline

25. U.S. Food and Drug Administation, *Establishing Safety and Compatibility of Passive Implants in the Magnetic Resonance (MR) Environment* (2014); www.fda.gov/downloads/MedicalDevices/DeviceRegulationandGuidance/GuidanceDocuments/UCM107708.pdf.

26. K. Puch-Kapst, R. Juran, B. Stoever, R. R. Wauer, Radiation exposure in 212 very low and extremely low birth weight infants. *Pediatrics* **124**, 1556–1564 (2009). doi:10.1542/peds.2008-1028 Medline

27. J. Pan, W. J. Tompkins, A real-time QRS detection algorithm. *IEEE Trans. Biomed. Eng.* **32**, 230–236 (1985). doi:10.1109/TBME.1985.325532 Medline

28. A. A. Kamal, J. B. Harness, G. Irving, A. J. Mearns, Skin photoplethysmography—A review. *Comput. Methods Programs Biomed.* **28**, 257–269 (1989). doi:10.1016/0169-2607(89)90159-4 Medline

29. J. S. Kim, Y. J. Chee, J. W. Park, J. W. Choi, K. S. Park, A new approach for non-intrusive monitoring of blood pressure on a toilet seat. *Physiol. Meas.* **27**, 203–211 (2006). doi:10.1088/0967-3334/27/2/010 Medline

30. L. A. Geddes, M. H. Voelz, C. F. Babbs, J. D. Bourland, W. A. Tacker, Pulse transit time as an indicator of arterial blood pressure. *Psychophysiology* **18**, 71–74 (1981). doi:10.1111/j.1469-8986.1981.tb01545.x Medline

31. J. M. Fanaroff, A. A. Fanaroff, Blood pressure disorders in the neonate: Hypotension and hypertension. *Semin. Fetal Neonatal Med.* **11**, 174–181 (2006). doi:10.1016/j.siny.2006.01.002

32. J. O’Shea, E. M. Dempsey, A comparison of blood pressure measurements in newborns. *Am. J. Perinatol.* **26**, 113–116 (2009). doi:10.1055/s-0028-1091391 Medline

33. J. S. Murray, C. Noonan, S. Quigley, M. A. Curley, Medical device-related hospital-acquired pressure ulcers in children: An integrative review. *J. Pediatr. Nurs.* **28**, 585–595 (2013). doi:10.1016/j.pedn.2013.05.004 Medline

34. M. C. Baserga, A. Puri, A. Sola, The use of topical nitroglycerin ointment to treat peripheral tissue ischemia secondary to arterial line complications in neonates. *J. Perinatol.* **22**, 416–419 (2002). doi:10.1038/sj.jp.7210713 Medline

35. L. A. Smith, P. J. Dawes, B. C. Galland, The use of pulse transit time in pediatric sleep studies: A systematic review. *Sleep Med. Rev.* **37**, 4–13 (2018). doi:10.1016/j.smrv.2016.11.006 Medline

36. C. F. Wippermann, D. Schranz, R. G. Huth, Evaluation of the pulse wave arrival time as a marker for blood pressure changes in critically ill infants and children. *J. Clin. Monit.* **11**, 324–328 (1995). doi:10.1007/BF01616991 Medline

37. B. C. Galland, E. Tan, B. J. Taylor, Pulse transit time and blood pressure changes following auditory-evoked subcortical arousal and waking of infants. *Sleep* **30**, 891–897 (2007). doi:10.1093/sleep/30.7.891 Medline

38. C. Ahlstrom, A. Johansson, F. Uhlin, T. Länne, P. Ask, Noninvasive investigation of blood pressure changes using the pulse wave transit time: A novel approach in the monitoring of hemodialysis patients. *J. Artif. Organs* **8**, 192–197 (2005). doi:10.1007/s10047-005-0301-4 Medline

39. W. Chen, T. Kobayashi, S. Ichikawa, Y. Takeuchi, T. Togawa, Continuous estimation of systolic blood pressure using the pulse arrival time and intermittent calibration. *Med. Biol. Eng. Comput.* **38**, 569–574 (2000). doi:10.1007/BF02345755 Medline

40. L. Sinclair, J. Crisp, J. Sinn, Variability in incubator humidity practices in the management of preterm infants. *J. Paediatr. Child Health* **45**, 535–540 (2009). doi:10.1111/j.1440-1754.2009.01555.x Medline

41. Sigma-Aldrich, Safety Data Sheet, 1-ethyl-3-methylimidazolium ethyl sulfate; www.sigmaaldrich.com/MSDS/MSDS/DisplayMSDSPage.do?country=US&language=en&productNumber=07784&brand=SIAL&PageToGoToURL=https%3A%2F%2Fwww.sigmaaldrich.com%2Fcatalog%2Fproduct%2Fsial%2F07784%3Flang%3Den.

42. Y. Zhang, R. C. Webb, H. Luo, Y. Xue, J. Kurniawan, N. H. Cho, S. Krishnan, Y. Li, Y. Huang, J. A. Rogers, Theoretical and experimental studies of epidermal heat flux sensors for measurements of core body temperature. *Adv. Healthc. Mater.* **5**, 119–127 (2016). doi:10.1002/adhm.201500110 Medline

43. L. Gao, Y. Zhang, V. Malyarchuk, L. Jia, K.-I. Jang, R. C. Webb, H. Fu, Y. Shi, G. Zhou, L. Shi, D. Shah, X. Huang, B. Xu, C. Yu, Y. Huang, J. A. Rogers, Epidermal photonic devices for quantitative imaging of temperature and thermal transport characteristics of the skin. *Nat. Commun.* **5**, 4938 (2014). doi:10.1038/ncomms5938 Medline

44. M. E. Van Valkenburg, R. L. Vaughn, M. Williams, J. S. Wilkes, Thermochemistry of ionic liquid heat-transfer fluids. *Thermochim. Acta* **425**, 181–188 (2005). doi:10.1016/j.tca.2004.11.013

45. A. Agrawal, O. Adetiba, H. Kim, H. Chen, J. G. Jacot, R. Verduzco, Stimuli-responsive liquid crystal elastomers for dynamic cell culture. *J. Mater. Res.* **30**, 453–462 (2015). doi:10.1557/jmr.2014.392

46. T. He, L. Cao, A problem of generalized magneto-thermoelastic thin slim strip subjected to a moving heat source. *Math. Comput. Model.* **49**, 1710–1720 (2009). doi:10.1016/j.mcm.2008.12.004

47. T. Kiserud, G. Piaggio, G. Carroli, M. Widmer, J. Carvalho, L. Neerup Jensen, D. Giordano, J. G. Cecatti, H. Abdel Aleem, S. A. Talegawkar, A. Benachi, A. Diemert, A. Tshefu Kitoto, J. Thinkhamrop, P. Lumbiganon, A. Tabor, A. Kriplani, R. Gonzalez Perez, K.

Hecher, M. A. Hanson, A. M. Gülmezoglu, L. D. Platt, The World Health Organization fetal growth charts: A multinational longitudinal study of ultrasound biometric measurements and estimated fetal weight. *PLOS Med.* **14**, e1002220 (2017). doi:10.1371/journal.pmed.1002220 Medline

48. Federal Communications Commission, Code of Federal Regulations Title 47 Part 15—Radio Frequency Devices; www.ecfr.gov/cgi-bin/text-idx?SID=0ea2993ba8323afa8443bc82dc4c5716&mc=true&node=pt47.1.15&rgn=div5.
